# Environmental unpredictability shapes glucocorticoid regulation across populations of tree swallows

**DOI:** 10.1038/s41598-020-70161-4

**Published:** 2020-08-13

**Authors:** Cedric Zimmer, Conor C. Taff, Daniel R. Ardia, Alexandra P. Rose, David A. Aborn, L. Scott Johnson, Maren N. Vitousek

**Affiliations:** 1 Department of Ecology and Evolutionary Biology, Cornell University, Ithaca, NY 14853 USA; 2 Cornell Lab of Ornithology, Ithaca, NY 14850 USA; 3 Department of Biology, Franklin and Marshall College, Lancaster, PA 17604 USA; 4 Institute of Arctic and Alpine Research, University of Colorado, Boulder, CO 80303 USA; 5 Biology, Geology and Environmental Science, The University of Tennessee Chattanooga, Chattanooga, TN 37403 USA; 6 Department of Biological Sciences, Towson University, Towson, MD 21252 USA

**Keywords:** Behavioural ecology, Ecophysiology, Evolutionary ecology

## Abstract

The ability to respond appropriately to challenges is an important contributor to fitness. Variation in the regulation of glucocorticoid hormones, which mediate the phenotypic response to challenges, can therefore influence the ability to persist in a given environment. We compared stress responsiveness in four populations of tree swallows (*Tachycineta bicolor*) breeding under different environmental conditions to evaluate support for different selective pressures in driving the evolution of glucocorticoid regulation. In accordance with the environmental unpredictability hypothesis, stronger stress responses were seen in more unpredictable environments. Contrary to the reproductive value hypothesis, the stress response was not lower in populations engaging in more valuable reproductive attempts. Populations with stronger stress responses also had stronger negative feedback, which supports a “mitigating” rather than a “magnifying” effect of negative feedback on stress responses. These results suggest that combining a robust stress response with strong negative feedback may be important for persisting in unpredictable or rapidly changing environments.

## Introduction

Global environmental changes are altering the habitats of many species^1^. For species with wide geographic ranges, intra-specific variation in life-history strategies resulting from historical selection might predispose some populations to be more or less susceptible to increasing environmental changes^2–4^. Environmental variation favours individuals that differentially allocate time and energy to reproduction and self-maintenance in order to maximize lifetime fitness^5–7^. Thus, characterizing differences in the regulation of this trade-off across environments is critical for understanding the mechanisms that have shaped phenotypic responses and that may allow successful adaptation and population persistence under rapid global changes. While the ultimate reasons for variation in life-history traits across environments and latitudes have been well studied, we have a limited understanding of the proximate mechanisms that underlie this variation^8,9^. Because environmental factors may influence the evolution of life-history traits by acting on physiological systems that integrate external conditions, hormones have been proposed to play a crucial role as mediators of life-history trade-offs^5,9,10^.

The hypothalamic–pituitary–adrenal (HPA) axis is a fundamental component of the endocrine system that forms an interface between an animal and its environment^11–13^. The HPA axis coordinates the response to energetic and other challenges mainly by regulating the production and release of glucocorticoids^14,15^. In non-stressed individuals, glucocorticoids are usually maintained at low levels to regulate energy balance and mediate foraging and other locomotor activities^14,16^. When facing unpredictable challenges, circulating glucocorticoids increase dramatically, promoting a suite of processes that facilitate responding to and recovering from these challenges^14,15^. When sustained, this stress response can trigger an emergency life-history stage in which breeding activities are usually reduced, and energy is redirected toward survival^15^. Thus, glucocorticoids—particularly in the presence of a stressor—have been widely predicted to mediate life history trade-offs between current and future reproduction.

While models of the stress response make clear predictions about redirecting effort, empirical studies have proved equivocal in linking environmental conditions to appropriate responses. One limitation of much empirical work to date is that it has focused on the relationship between glucocorticoids and fitness in a single context (i.e., a single population, year, or environment)^12,17^. The factors that shape these relationships are best understood by measuring glucocorticoids across different environmental and life-history contexts^18–20^. Because the costs and benefits of mounting a robust glucocorticoid stress response are likely to differ across environments and species, populations are predicted to differ in how they regulate glucocorticoids^19,21^. For instance, a comparative study in birds found a positive association between latitude and the glucocorticoid response to a standardized restraint stressor (acute challenge that activates the HPA axis resulting in stress-induced glucocorticoids increase) during breeding^22^. However, willow warblers (*Phylloscopus trochilus*) breeding in northern Sweden—where the breeding season is shorter and environmental conditions less predictable—show lower stress-induced glucocorticoid levels than those breeding in southern Sweden, where conditions are more predictable^23^. Breuner and colleagues^24^ compared males from three populations of white-crowned sparrows (*Zonotrichia leucophrys*) breeding at different latitudes from California to Alaska. They found that males had similar stress-induced glucocorticoid levels, but differed in their corticosteroid-binding globulin and intracellular receptor affinity, suggesting that HPA axis regulation varied across the populations. Thus, while the stress response appears to vary with latitude, additional research is needed to determine which of the many potential selective pressures that covary with latitude are driving this variation.

One factor thought to be particularly important in shaping the costs and benefits of stress responsiveness during breeding is how valuable each breeding attempt is to lifetime reproductive effort (the “brood value” or “reproductive value” hypothesis^13,22^). The reproductive value hypothesis predicts that the stress response differs based on the proportion of lifetime reproductive effort represented by a single breeding attempt. According to this hypothesis, because high glucocorticoid levels can be deleterious to reproduction by diverting energy away from breeding activities, organisms engaging in more valuable reproductive attempts should have lower stress-induced glucocorticoids to avoid jeopardising the current breeding attempt^22^. The relative value of a single reproductive event depends on both the length of the breeding season, which influences the number of potential reproductive attempts an individual can engage in per season, and its lifespan. A shorter breeding season—which results in less opportunity to engage in multiple successive reproductive attempts or to reinitiate breeding after a failed attempt—increases the relative value of each reproductive attempt^12,13,24^. Therefore, the reproductive value hypothesis predicts that populations living in environments with shorter breeding seasons will show lower stress-induced glucocorticoid levels during breeding^11,22^. Although reproductive value has been supported as a driver of HPA axis variation both within and across species (e.g.^22,25,26^), this relationship is not universal. The degree to which the stress response is downregulated based on reproductive value may depend on whether mounting an HPA response would help individuals to escape from or mitigate the stressor (e.g.^19,27,28^). The impact of high glucocorticoid levels on reproduction may also depend on the strength of negative feedback: work in a temperate-breeding population of tree swallows (*Tachycineta bicolor*) suggests that maintaining a strong stress response, if coupled with strong negative feedback, supports the maintenance of reproductive activity following challenges^29^.

We recently suggested another factor that may impact the benefit of mounting a strong stress response: the frequency with which organisms face major unpredictable challenges that can be mitigated by glucocorticoids (“matched” challenges^19^). While individuals can prepare for predictable changes in environmental harshness using a variety of mechanisms, HPA axis activation may be particularly important for mounting rapid and generalized responses to unpredictable challenges, such as sudden storms or declines in food availability^30^. Thus, the “environmental unpredictability” hypothesis predicts that species or populations that inhabit more unpredictable environments will have higher stress-induced glucocorticoid levels. It is worth emphasizing that this prediction is limited to environmental challenges for which a glucocorticoid response facilitates effective coping (which may differ across both stressor types and life histories). We are aware of only one previous study that has directly quantified and tested the role of environmental variation in predicting variation in stress-induced glucocorticoids (and none that have quantified unpredictability). That study, a phylogenetic comparative analysis of the relative support for these and other factors in shaping glucocorticoid variation across vertebrates, found that reproductive value better predicted large-scale variation in stress-induced glucocorticoids than environmental variability^20^. However, the relative roles of different selective pressures in shaping glucocorticoid variation are expected to vary across species. Disentangling the relative roles of reproductive value and environmental unpredictability in shaping glucocorticoid regulation will require comparing populations of the same species inhabiting different environments. It is also important to note that the environmental unpredictability hypothesis is not mutually exclusive with a role for reproductive value in shaping HPA axis regulation—indeed, a wide variety of other selective pressures may also influence how glucocorticoids are regulated across species.

Central to the hormonal mediation of life history is an understanding of the potential costs of the stress response. The costs of the stress response likely depend not only on maximum glucocorticoid levels but also on the duration of exposure to high levels, which is influenced by the strength of negative feedback. Negative feedback is triggered after activation of the HPA axis and is coordinated by glucocorticoids binding to receptors in the brain inducing a decrease in circulating glucocorticoids^31–33^. Despite increasing evidence that differences in the strength of negative feedback affect aspects of health and performance^34–36^, its functional effects have been largely neglected in free-living organisms (but see^29,37^). We are not aware of any previous studies that have assessed how negative feedback varies across environments. We hypothesised that differences in negative feedback efficacy could either mitigate or magnify the costs of a stress response. Strong negative feedback could serve to mitigate the costs of mounting a robust stress response by inducing a fast decrease in circulating glucocorticoids. This could be particularly important when a strong stress response is required to cope effectively with frequent unpredictable short-term challenges. In this case, strong negative feedback may make it possible to avoid the negative effects of sustained glucocorticoid elevation and hence recover quickly and resume critical activities such as breeding. A recent study within a single population of tree swallows supported the mitigating hypothesis: incubating females that exhibited both a robust stress response and strong negative feedback were less likely to abandon reproductive attempts when facing stressors^29^. Accordingly, individuals breeding in highly variable environments are predicted to show elevated stress responses followed by strong negative feedback. Alternatively, coupling an elevated stress response with weak negative feedback could serve to magnify the effects of the stress response. This could be adaptive if mounting a longer stress response facilitates avoiding or alleviating severe challenges; for example, if a more robust response enhances sensitivity to environmental cues^21,38^.

Here, we compared the support for two sets of predictions about how HPA axis regulation differs across populations and environments in breeding tree swallows. First, we asked whether variation in the glucocorticoid stress response (peak stress-induced glucocorticoids) across populations supports the reproductive value hypothesis or the environmental unpredictability hypothesis. Second, we assessed whether negative feedback varies across populations, and if so, whether the patterns suggested a magnifying or mitigating effect on the stress response. The tree swallow, a common passerine bird that breeds across much of North America, is an ideal species in which to test these predictions as it breeds along an expansive latitudinal as well as elevational gradient within the temperate zone. As such, different populations face different amounts of time available for reproduction as well as differing levels of environmental unpredictability. We compared populations breeding in Tennessee, New York, Wyoming and Alaska. We assessed females’ HPA axis activity by measuring baseline glucocorticoids, stress responses, and negative feedback during two life history stages: incubation and nestling rearing. We measured HPA axis activity at these two stages as glucocorticoid regulation can differ across life history subtages and glucocorticoid trait expression at one stage could influence expression at other stages^39^. These modifications may be due to changes in the relative importance of the forces (e.g., reproductive value vs. environmental unpredictability) shaping these traits as reproductive value typically increases over the breeding period. In order to test whether between-population differences in the stress response are better predicted by differences in the time available for reproduction or environmental unpredictability, we characterised both parameters in each population. We first determined the length of the breeding season in each population. We used the length of the breeding season as a proxy of reproductive value at populational level as we were not able to calculate reproductive value based on life span. Breeding season length is a good proxy for differences in reproductive value across populations breeding at different latitudes^11^. Because increasing reproductive value is generally associated with higher parental investment^40–42^ and thus potentially with higher reproductive success, we also determined breeding effort and success. To characterise differences in environmental predictability, we calculated an index of unpredictability for different weather variables using historical weather data for each site. We predicted that if variation in the magnitude of the glucocorticoid stress response is predominantly shaped by reproductive value, females breeding at the two sites with a relatively short breeding season (Alaska and Wyoming) would mount a lower acute stress response^20,22^. Conversely, if environmental unpredictability plays a greater role, we expected the opposite pattern: females breeding in the more unpredictable environments of Alaska and Wyoming would have higher stress-induced corticosterone levels. Concerning negative feedback, the mitigation hypothesis predicts strong negative feedback in populations with greater stress responses. The magnifying hypothesis, in contrast, predicts weaker feedback in the face of greater stress responses.

## Results

### Environmental unpredictability

**Figure 1:**
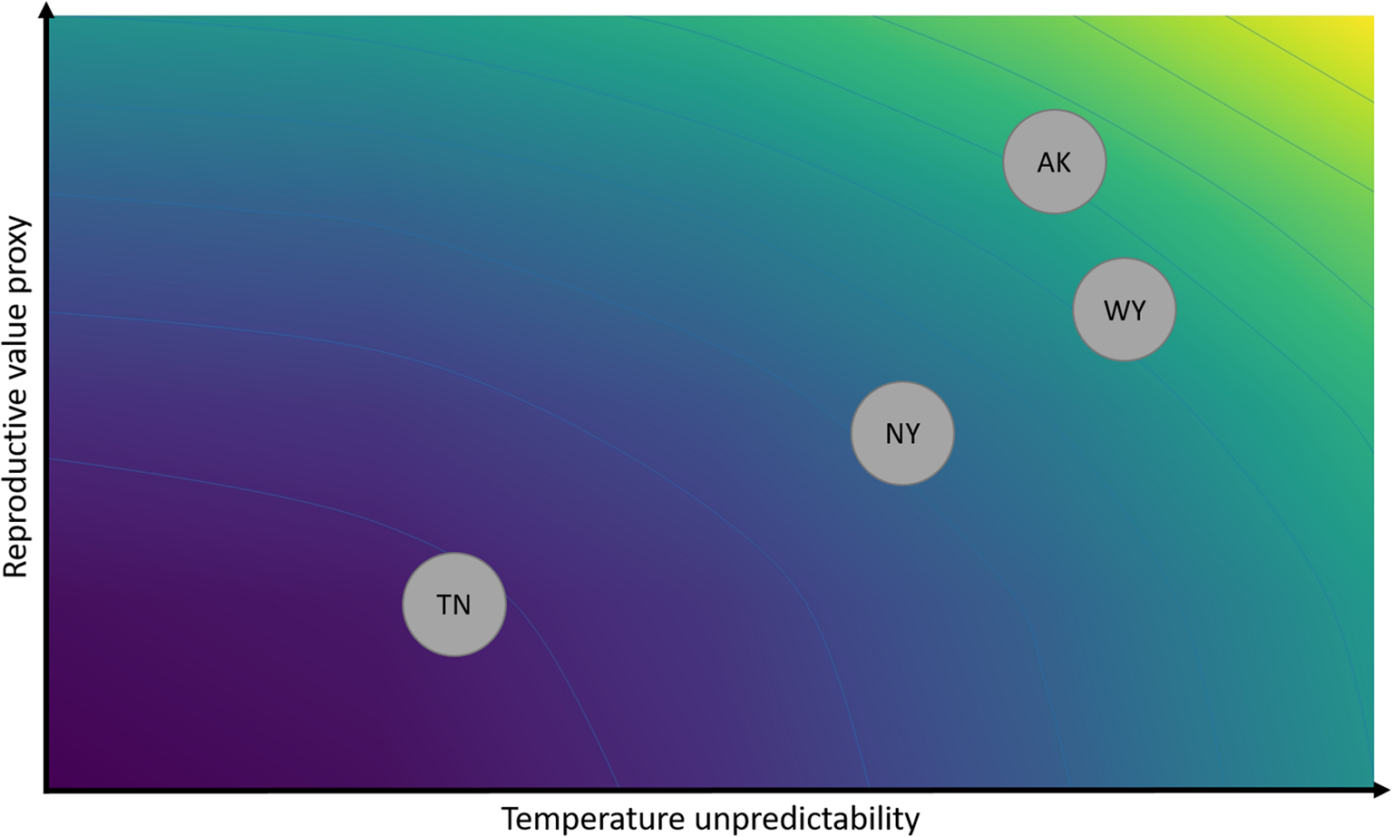
Plot illustrating the position of the four populations [Tennessee (TN), New York (NY), Wyoming (WY) and Alaska (AK)] along gradients of increasing temperature unpredictability (x axis, based on the average of the different temperature unpredictability measures) and a proxy of reproductive value based on breeding season length (y axis). Warmer colour indicates higher environmental unpredictability and higher reproductive value.

Unpredictability indices, calculated from weather data at each site using general additive models^43^, revealed that the unpredictability of temperature during the breeding season was the lowest in Tennessee and the highest in Wyoming and Alaska (Table 1; Fig. 1). Temperature unpredictability in New York was intermediate, but closer to Alaska and Wyoming than to Tennessee (Table 1).

**Table 1:** Unpredictability and average value (range) of daily average temperature, daily average active time (between 0600 and 2000) temperature, daily maximum temperature, and the length of the breeding season (proxy of reproductive bvalue) for the field sites in Tennessee, New York, Wyoming and Alaska. Higher SD_res_ value indicates greater unpredictability.

	Tennessee	New York	Wyoming	Alaska
SD_res_ average daily temperature	0.18	0.33	0.41	0.38
Average daily temperature	18.9 (− 2.1 to 30.4)	12.9 (− 5.7 to 27.9)	9.5 (− 8.7 to 22.3)	11.8 (− 6.9 to 26.3)
SD_res_ average daytime temperature	0.21	0.42	0.53	0.50
Average daytime temperature	20.1 (0.2 to 34.1)	15.4 (− 3.6 to 31.9)	13.3 (− 7.7 to 28.6)	14.7 (− 4.4 to 30.0)
SD_res_ maximum daily temperature	0.21	0.38	0.51	0.47
Maximum daily temperature	24.1 (3.3 to 38.3)	18.8 (− 1.3 to 34.4)	15.9 (− 6.7 to 32.2)	17.8 (− 2.7 to 33.3)
Length of breeding season (days)	99	74	70	66

### Reproductive value

Total length of the breeding season differed between populations (χ^2^_3_ = 8.49, p = 0.037) with a longer breeding season in TN (99 days) and the shortest in AK (66 days). Breeding season lengths in NY (74 days) and WY (70 days) were intermediate, but more similar to AK than TN. Overall, these patterns suggest that reproductive value was the lowest in TN and relatively similar across the other three populations (Fig. 1).

**Figure 2:**
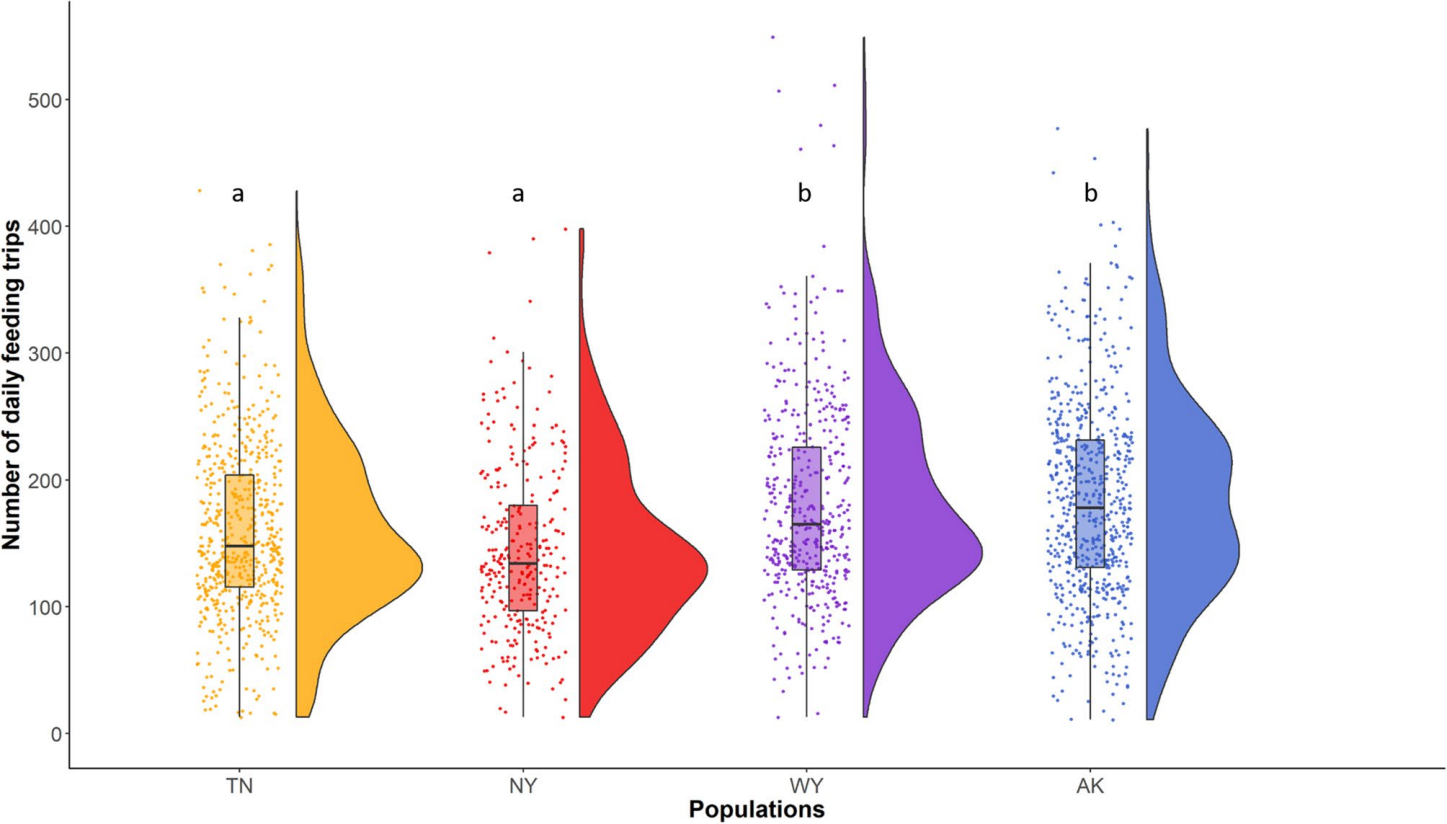
Number of daily feeding trips for females in Tennessee (orange), New York (red), Wyoming (purple), and Alaska (blue), calculated from the total number of raw visits recorded (329,047). Points overlaying the boxplot show raw data and the half-split violin shows the probability density function. Different letters indicate significant differences between populations.

There were no differences in clutch size or brood size at hatching between populations (χ^2^ ≤ 5.90, p ≥ 0.12, , see supplementary material). Because of an extended period of cold, wet weather that occurred during the incubation stages of most females in WY, hatching success (χ^2^_3,250_ = 29.24, p < 0.0001) was lower in WY (50.7%, 38 of 75 nests) than in the other three populations (TN: 82.2%, 60 of 73 nets; NY: 79.5%, 31 of 39 nests; AK: 84.4%, 54 of 64 nests; z ≥ 3.58, p ≤ 0.002). Provisioning effort differed across populations (F_3,155.4_ = 6.00, p = 0.0007). Females in AK and WY provisioned at higher rates than those in NY and TN (effect size = 0.27 ± 0.03, Fig. 2, Fig. S5).

Nestling body mass differed across populations, mirroring the observed differences in female provisioning rates (F_3,128.5_ = 4.02, p = 0.009). On day 12 post-hatching, nestlings were heaviest in AK (21.2 ± 0.2 g) and WY (20.6 ± 0.2 g), and lightest in NY (18.7 ± 0.5 g) and TN (19.6 ± 0.2 g). Among nests in which one or more eggs hatched, the number of nests in which one or more nestlings fledged also differed among populations (χ^2^_3,250_ = 22.49, p < 0.0001); fledging success was lower in NY (35.5%, 11 of 31 nests) than in the other populations (TN: 76.7%, 46 of 60 nests, AK: 80.4%, 41 of 51 nests, WY: 80.0%, 28 of 35 nests; t ≥ 3.30, p ≤ 0.005).

### Corticosterone regulation

**Figure 3:**
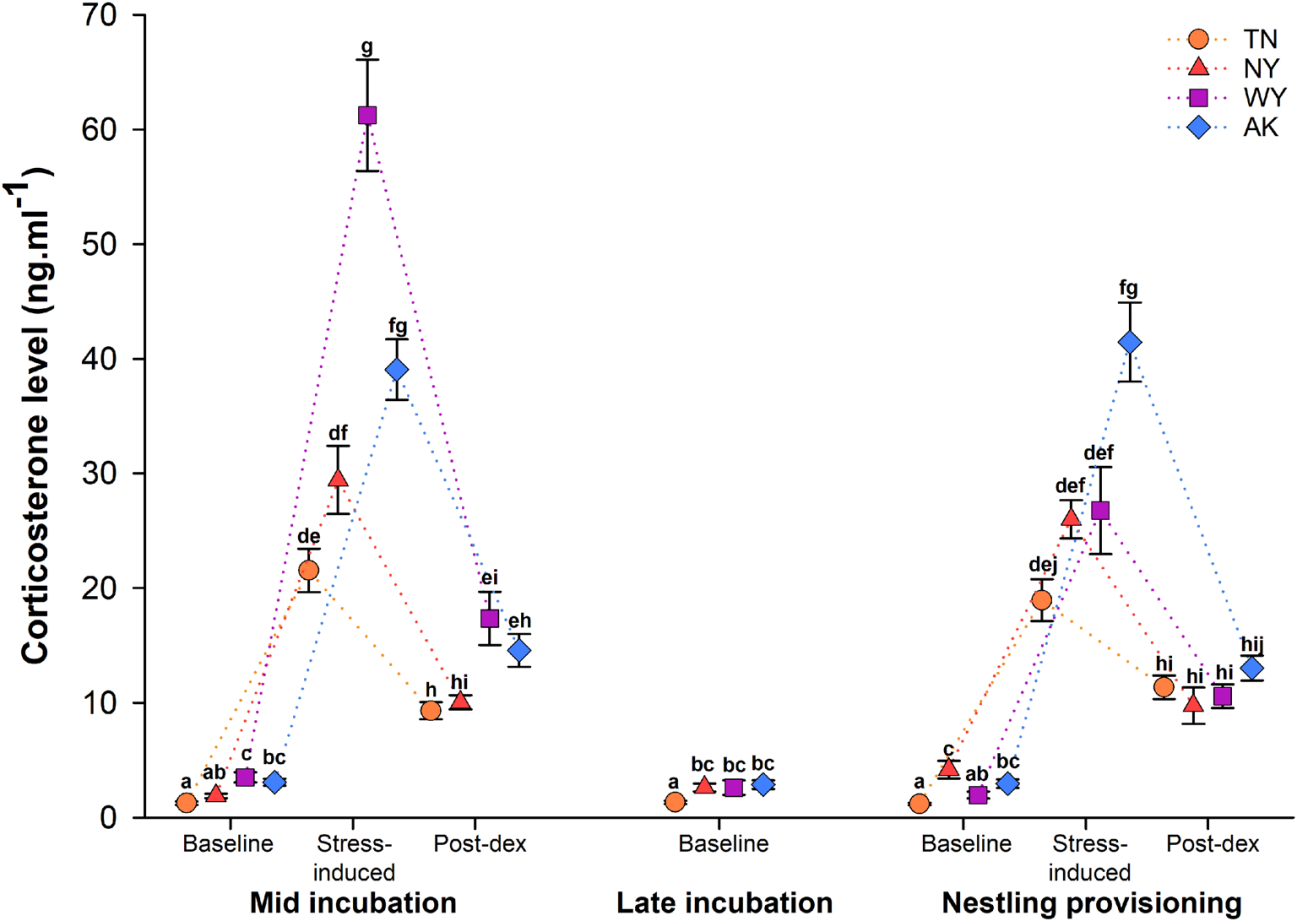
Corticosterone levels (mean ± SE) of females when captured at three points during the reproductive cycle (mid incubation: day 6–7 of incubation, late incubation: day 12–13 of incubation, and nestling provisioning: day 6 post-hatch) in Tennessee (TN), New York (NY), Wyoming (WY), and Alaska (AK). At all captures baseline corticosterone levels were measured. During the first and third captures, stress-induced corticosterone after 30 min of restraint and 30 min after injection of dexamethasone (post-dex) were also measured. Different letters indicate significant differences.

As part of a separate study, females were exposed to experimental stressors after the first capture. Treatment was included in all the models and did not affect females’ corticosterone phenotype (Table S1, S2, S3). Females’ corticosterone phenotypes differed between populations (Table S1). This difference was influenced by the life history substage during which the female was captured and corticosterone sample type (baseline, stress-induced, post-dexamethasone (post-dex); population × life history substage × sample: F_12,1196_ = 4.07, p < 0.0001; Fig. 3). To directly compare the degree to which temperature unpredictability and reproductive value predicted corticosterone phenotypes across populations, we ran similar models in which population identity was replaced by average temperature unpredictability or by total breeding season length as continuous variables. Glucocorticoid levels increased with increasing average temperature unpredictability (β = 2.03 [1.32–2.74], F_1,280.3_ = 18.96, p < 0.0001; Table S2, S15; Fig. S1) and decreased as breeding season lengthened (β = − 0.03 [− 0.05 to − 0.01], F_1,268.3_ = 29.72, p < 0.0001; Table S3, S16; Fig. S1). The model that included average temperature unpredictability was substantially better at predicting glucocorticoid levels than the model with total breeding season length (ΔAIC = − 382.1).

Across sampling periods, baseline corticosterone levels tended to be lower in Tennessee than in the other three populations (Fig. 3, see supplementary material for statistical analyses).

**Figure 4:**
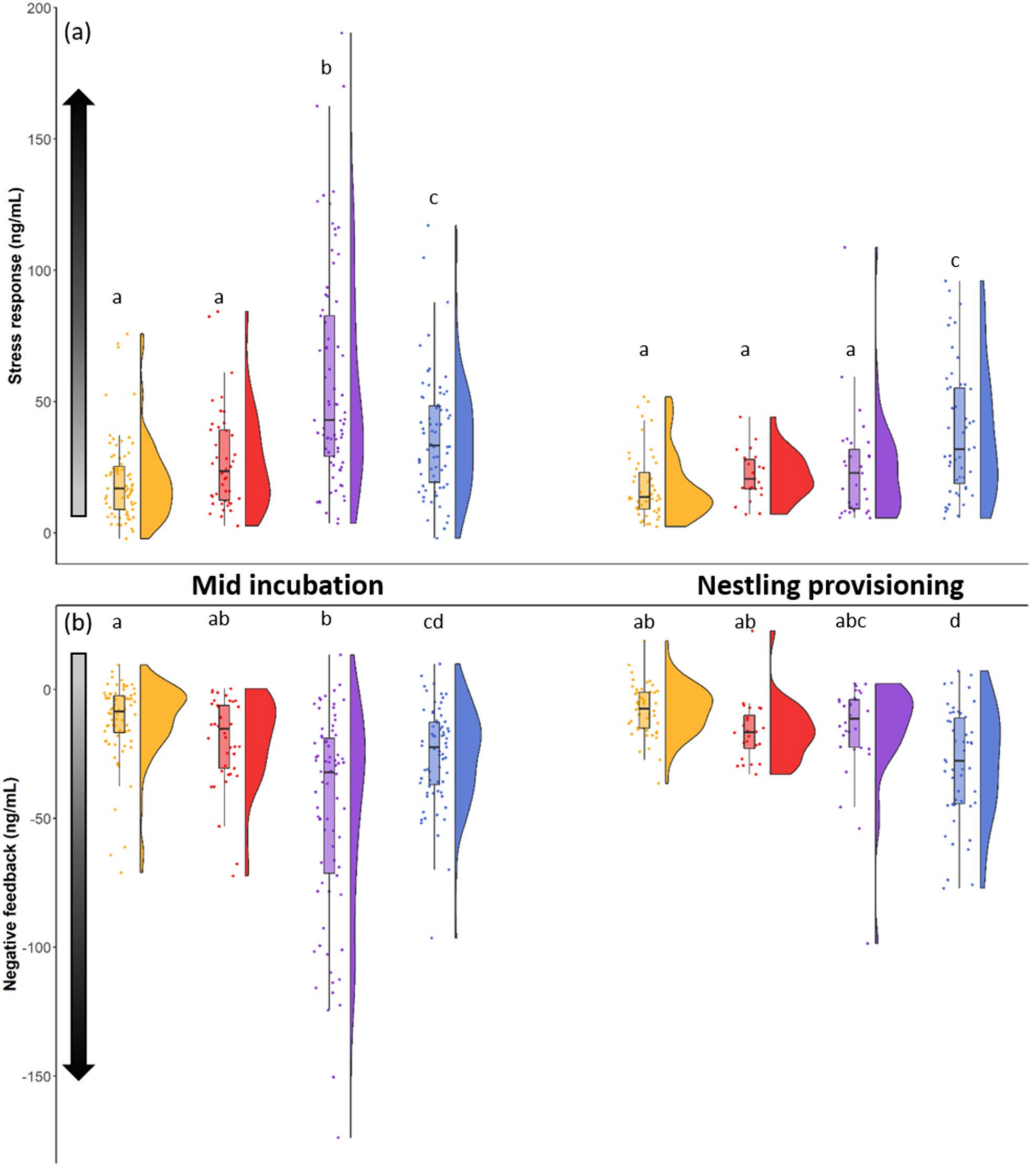
Females’ glucocorticoid stress responses (**a**) and the strength of negative feedback (**b**) in Tennessee (orange), New York (red), Wyoming (purple) and Alaska (blue) during mid incubation and nestling provisioning. Points overlaying the boxplot show raw data and the half-split violin shows the probability density function. Shaded arrows along the y-axis denote increasing strength of the stress response and negative feedback with increasing darkness. Different letters indicate significant differences.

Circulating stress-induced corticosterone levels tended to be higher in populations with higher temperature unpredictability and those with greater reproductive value, especially during incubation (Fig. 3; see supplementary material for statistical analyses). Corticosterone stress responses (the difference between stress-induced and baseline corticosterone) showed a similar pattern. Overall, stress responses differed between populations and life history substages (population × life history substage: F_3,326.7_ = 5.49, p = 0.001; Fig. 4a; Table S4). During mid-incubation, stress responses were highest in WY than in the other populations (t ≥ 5.08, p < 0.0001; Fig. 4a) and intermediate in AK (higher than in TN and NY: t ≥ 2.71, p ≤ 0.044; Fig. 3a). During nestling provisioning, females in AK had a stronger stress response than females in all other populations (t ≥ 3.01, p ≤ 0.048; Fig. 4a). Because it is currently unclear whether the strength of the stress response is more appropriately assessed via total stress-induced corticosterone levels or as their stress-induced increase over baseline (stress response), we ran the same model with stress-induced corticosterone as a dependent variable and baseline corticosterone as a covariate. This model gave similar results than the model on the stress response (Table S5). Then, we ran models for the stress response in which population identity was replaced by average temperature unpredictability or by total breeding season length as continuous variables. The strength of the stress response increased with increasing temperature unpredictability (β = 1.49 [0.65–2.33], F_1,257_ = 13.59, p < 0.0001; Table S6, S17; Fig. S2) and decreased as breeding season lengthened (β = − 0.014 [− 0.029 to − 0.002], F_1,249.9_ = 16.08, p < 0.0001; Table S7, S18; Fig. S2). Model comparisons revealed that the model that included average temperature unpredictability was substantially better at predicting the magnitude of the glucocorticoid stress response than the model with total breeding season length (ΔAIC = − 113.17).

Circulating corticosterone levels after dexamethasone injection did not differ between the four populations (t ≤ 1.73, p ≥ 0.99; Fig. 3). However, negative feedback, measured as the decrease in corticosterone following dexamethasone injection, differed between populations and life history substages (population × life history substage: F_3,267_ = 11.63, p < 0.0001; Fig. 4b, Table S8). During mid-incubation, negative feedback was stronger in WY than in all other populations (t ≥ 5.36, p < 0.0001; Fig. 4b). At this time period, negative feedback in AK was higher than in TN (but not NY); NY and TN did not differ in negative feedback (pairwise comparisons: AK vs. TN: t = 3.60, p = 0.016, AK vs. NY: t = 0.69, p = 0.99, TN vs. NY: t = 1.55, p = 0.77; Fig. 4b). During the nestling provisioning period, females in AK had stronger negative feedback than females in all other populations (t ≥ 1.96, p ≤ 0.05; Fig. 4b); at this time point negative feedback did not differ among the other populations (t ≤ 0.71, p ≥ 0.97; Fig. 4b). Because it is currently unclear which of several measures of negative feedback best captures meaningful variation^44^, we ran similar models using two additional measures of negative feedback: post-dex corticosterone with stress-induced corticosterone as a covariate, and the percentage change in corticosterone between the stress-induced and post-dex sampling periods. Overall, these models gave similar results to the model presented above, where negative feedback was calculated as the decrease from stress-induced to post-dex levels (Tables S9, S10). Then, we ran models using average temperature unpredictability or total breeding season length as continuous predictors, which found that the strength of negative feedback increased with temperature unpredictability (β = − 31.45 [− 61.10 to − 1.80], F_1,300.8_ = 8.33, p = 0.004; Table S11, S21; Fig. S3) and decreased as breeding season lengthened (β = 0.55 [0.01–1.10], F_1,277.8_ = 6.54, p = 0.011; Table S12, S22; Fig. S3). As seen for all other glucocorticoid traits, the model that included average temperature unpredictability was significantly better at predicting glucocorticoid levels than the model that included total breeding season length (ΔAIC = − 116.11).

Within populations, the magnitude of the stress response did not change between substages of the reproductive period (t ≤ 1.49, p ≥ 0.81; Fig. 4a), except in WY where it decreased between incubation and nestling provisioning (t = 7.09, p < 0.0001; Fig. 4a). Negative feedback efficacy also did not change between life history substages within populations (t ≤ 1.57, p ≥ 0.76; Fig. 4b). See supplementary material for detailed information about within population corticosterone changes across the breeding season.

Correlations between the different measures of the HPA axis differed between populations and life history substages. Most of the measured components of the HPA axis were not correlated or weakly to moderately correlated (Table 2). However, the strength of the stress response and the efficacy of negative feedback were positively correlated in all populations at both life history substages (Table 2).

**Table 2:** Correlations among components of the HPA axis within each population at each life history substage. Data are R^2^, p-value. Bold indicates significant correlations.

	Mid incubation			Nestling provisioning		
	Stress-induced (R^2^, p-value)	Post-dex (R^2^, p-value)	Negative feedback (R^2^, p-value)	Stress-induced (R^2^, p-value)	Post-dex (R^2^, p-value)	Negative feedback (R^2^, p-value)
**Tennessee**						
Baseline	0.001, 0.94	0.005, 0.54		**0.09, 0.03**	**0.18, 0.002**	
Stress-induced		**0.08, 0.02**	**0.84, < 0.0001**		**0.27, 0.0001**	**0.69, < 0.0001**
Post-dex			0.01, 0.33			0.02, 0.37
Stress response	**0.99, < 0.0001**		**0.84, < 0.0001**	**0.99, < 0.0001**		**0.70, < 0.0001**
**New York**						
Baseline	0.02, 0.43	0.02, 0.41		0.001, 0.89	0.03, 0.43	
Stress-induced		**0.15, 0.01**	**0.96, < 0.0001**		0.01, 0.62	**0.57, < 0.0001**
Post-dex			**0.17, 0.01**			0.52, 0.0001
Stress response	**0.99, < 0.0001**		**0.96, < 0.0001**	**0.81, < 0.0001**		**0.50, 0.0008**
**Wyoming**						
Baseline	**0.07, 0.02**	0.005, 0.55		0.11, 0.07	0.01, 0.60	
Stress-induced		0.001, 0.91	**0.92, < 0.0001**		0.08, 0.12	**0.92, < 0.0001**
Post-dex			0.001, 0.48			0.0002, 0.95
Stress response	**0.99, < 0.0001**		**0.91, < 0.0001**	**0.99, < 0.0001**		**0.92, < 0.0001**
**Alaska**						
Baseline	0.001, 0.78	0.003, 0.66		0.01, 0.43	0.007, 0.54	
Stress-induced		0.03, 0.15	**0.71, < 0.0001**		**0.25, 0.0001**	**0.91, < 0.0001**
Post-dex			0.005, 0.59			0.06, 0.10
Stress response	**0.88, < 0.0001**		**0.69, < 0.0001**	**0.9, < 0.0001**		**0.90, < 0.0001**

## Discussion

In the current context of global changes, it is important to understand how organisms have evolved to cope with environmental challenges. Our results suggest that the unpredictability of environmental challenges could affect how selection shapes glucocorticoid regulation. First, we showed that female tree swallows breeding in environments with higher temperature unpredictability had higher glucocorticoid concentrations in response to acute challenges. There was no support for the hypothesis that populations that engage in higher value and more time limited breeding attempts will mount weaker stress responses. Second, we found that females in populations with higher stress responses also have stronger negative feedback, which likely functions to limit potential damage caused by high glucocorticoid levels. The combination of high stress-induced glucocorticoid levels followed by the induction of strong negative feedback may allow females to cope effectively with frequent unpredictable challenges, and to recover faster in order to continue breeding activities. We predict that this phenotype is most strongly favoured in environments with both high unpredictability and greater time constraints on reproduction.

Across the four study populations we found differences in environment and life history that could result in differing selective pressures. Birds breeding in Alaska and in the mountains of Wyoming faced more unpredictable temperatures than birds in Tennessee; New York conditions were intermediate. The time constraints on reproduction differed similarly across populations, resulting in higher apparent reproductive value in Alaska and Wyoming and lowest apparent reproductive value in Tennessee. As predicted by life history theory (and consistent with previous comparative work in tree swallows)^45–48^, females in Alaska and Wyoming showed higher parental investment (measured as offspring feeding rate) and reared larger offspring than those in New York and Tennessee.

At our two sites with stronger time constraints on reproduction (higher reproductive value) and high temperature unpredictability (AK and WY) females mounted the strongest glucocorticoid stress responses, measured as both stress-induced glucocorticoid concentrations and their increase over baseline (measures which were highly correlated). This result suggests that in female tree swallows, environmental unpredictability could be a stronger force shaping the peak glucocorticoid response to challenges than reproductive value—which is instead predicted to select for suppressed stress responses when reproduction is more time constrained. This is consistent with the finding that the magnitude of the stress response did not change between the incubation and nestling rearing periods in three of the four study populations, despite reproductive value typically increasing over this time period (at least in populations in which renesting may be possible in the event a first attempt fails). Although both within-species and large-scale comparative analyses have found that stress responsiveness is negatively predicted by reproductive value^20,22,23,26^, this pattern is not ubiquituous. The positive relationship seen here could indicate that stress-induced corticosterone does not impair reproductive effort in tree swallows. Alternatively, the benefit of mounting a strong stress response in unpredictable environments could outweigh its deleterious effects on reproduction. We predict that differences in the relative importance of environmental unpredictability and reproductive value in shaping stress responsiveness varies across species, sex and breeding system. For instance, we predict that the stress responsiveness of income breeders will be more strongly influenced by environmental unpredictability than that of capital breeders. Similarly, we predict that stress responsiveness will be particularly closely tied to environmental unpredictability in species that rely on critical food sources impacted by short-term environmental fluctuations (e.g., aerial insectivores).

A robust stress response could allow appropriate responses to challenges, but might also result in associated reproductive costs^13^. However, we found no evidence that maintaining a robust stress response had negative effects on reproduction. Reproductive success did not vary consistently across populations; instead, the differences that we saw in hatching success (lowest in WY) and fledging success (lowest in NY) mirrored temporary periods of inclement weather in those populations. Consistent with life history theory, females in Alaska and Wyoming showed a higher investment in nestlings. Nevertheless, it is possible that the costs of maintaining an elevated stress response would manifest under more prolonged stressful conditions, or that it imposes longer-term costs (e.g., accelerated telomere shortening or senescence).

We found support for a mitigating effect of negative feedback on stress responsiveness: female tree swallows breeding in Alaska and Wyoming, where both time constraints on reproduction and temperature unpredictability were the highest, showed both a high stress response and strong negative feedback. Stronger negative feedback allows for a faster decrease in circulating glucocorticoids, potentially reducing the costs associated with sustained glucocorticoid elevation. Thus, strong negative feedback is predicted to be particularly beneficial for individuals with a strong stress response^29^. We recently demonstrated that individual variation in negative feedback efficacy predicts the speed of recovery of the HPA axis from repeated transient stressors, stress resilience, and reproductive success in female tree swallows^21,29,49^. These findings suggest that negative feedback regulation may be critical for coping with pertubances particularly in unpredictably challenging environments. The current study suggest that environmental unpredictability may have shaped this glucocorticoids regulation on larger scales.

Taken together, our results suggest that when environmental conditions become more unpredictable female tree swallows couple high magnitude stress responses and strong negative feedback, instead of decreasing the hormonal response to challenges. Through strong negative feedback individuals could limit the reproductive costs of glucocorticoid exposure, and resume reproductive activities as soon as the stressor has passed. Thus, this combination of glucocorticoid regulatory traits may enable individuals living in harsher environments to balance the challenges of high environmental unpredictability and a brief reproductive period in order to maximise survival and reproductive success. While we focused here on two factors previously predicted to shape variation in the stress response it is important to note that there are almost certainly other selective pressures at play as well. For example, food availability and mean temperature, which could covary with both environmental unpredictability and breeding season length, likely differ between these environments. The design of this study, where carefully controlled sampling was conducted across a limited number of populations, also limits the strength of the conclusions that we can draw. Follow-up studies comparing a broader range of populations could provide stronger support for the hypothesis that environmental unpredictability is a major factor shaping variation in stress responses.

Our findings are also consistent with the idea that interactions among glucocorticoid regulatory elements may be important for appropriately responding to challenges, and for fitness^21,29,39^. In all four populations the strength of the stress response and the efficacy of negative feedback positively covary; however, there were weakly positive or no phenotypic correlations between stress-induced and post-dexamethasone corticosterone levels. Covariation in components of glucocorticoid regulation could result from similar regulatory pathways, as negative feedback is regulated by glucocorticoids binding to glucocorticoid receptors^32,33^. As such, higher glucocorticoid levels could activate more receptors, inducing faster negative feedback^31,33^. However, it has been suggested that different components of the HPA axis are modulated independently (e.g.^33^). Therefore, the phenotypic correlations seen here could also result from selection favouring combinations of these traits. Determining whether stress-induced and post-dexamethasone corticosterone levels are genetically correlated and whether glucocorticoid profiles covary with receptor expression could help to illuminate the flexibility and physiological underpinnings of these traits and their potential to respond to selection.

Baseline glucocorticoid levels also differed across populations. However, as both the reproductive value hypothesis and the environmental unpredictability hypothesis predict higher baseline glucocorticoid levels in Alaska and Wyoming, where the season is short, temperatures colder and weather unpredictable, these patterns do not allow us to differentiate between the role of these forces in shaping glucocorticoid evolution. Elevated baseline glucocorticoid levels have been shown to support energetically demanding activities, and are associated with more challenging conditions and increased investment in reproduction within and across species^18,20,50–52^. In contrast, in Tennessee, in which tree swallows experience a long breeding season and more predictable conditions baseline glucocorticoid levels were consistently low throughout the reproductive period.

It is also possible that the observed differences in HPA axis activity between populations were influenced by the conditions birds were experiencing during the study period. For instance, females in Wyoming at the first capture had the highest baseline and stress-induced glucocorticoid levels, which is consistent with these birds mounting a temporary response to the inclement weather that occurred during the peak period of incubation captures at this study site. Females in Wyoming also had the lowest body mass at the first capture (Fig. S4). However, these patterns were no longer apparent by the third capture, after the unusually cold and rainy period had ended. It is worth noting that this study focused on female tree swallows only. While males show similar offspring provisioning rates to those of their mates, female tree swallows are the sole incubators in most populations, including those studied here, and devote more time to brooding young. It is possible that sex differences in reproductive investment would result in different selective pressures shaping HPA axis regulation in males and females.

Overall, these findings provide some support for the hypotheses that environmental unpredictability may be a critical factor in shaping glucocorticoid stress responses, and that selection favouring strong negative feedback in more stress responsive individuals could serve as a mechanism to mitigate the costs of mounting a strong stress response. Our results are also in accordance with the hypothesis that negative feedback and the dynamic regulation of glucocorticoids are important for coping with challenging conditions^21,37,53^. In the current context of global changes, intraspecific differences in the response to stressors may be particularly important for survival or for the ability to adapt to new conditions^4,54^. Our results suggest that this phenotype (elevated stress response and strong negative feedback) could have been selected for in females breeding in unpredictable environments and might therefore be expected to become increasingly common over time, assuming genetic variation exists. However, as climate change affects both the length of the breeding season^55^ and environmental predictability^56^, populations may face rapidly changing regimes of selection on glucocorticoid regulation outside the bounds of evolutionary history. Confirming that selection is occurring in these populations will require testing whether among individual differences in glucocorticoid phenotype affect fitness. Ultimately, determining the evolutionary causes and consequences of differences in glucocorticoid levels within and among populations will help to reveal how selection drives HPA axis regulation and whether the history of selection on hormonal regulation influences the ability to cope with unpredictable or changing environments.

## Methods

### Populations

Field data were collected from 2016 to 2018 in four different populations of tree swallows breeding in nest-boxes. Populations were located in Chattanooga, Tennessee (TN) (35.1°N, 85.2°W, 206 m elevation, established in 2006, 2018: n = 73), Ithaca, New York (NY) (42.5°N, 76.5°W, 340 m elevation, established in 1986, 2016: n = 42), Burgess Junction, Wyoming (WY) (44.5°N, 107.3°W, 2451 m elevation established in 1995, 2018, n = 77) and in McCarthy, Alaska (AK) (61.4°N, 143.3°W, 445 m elevation, established in 1993, 2016: n = 28, 2017: n = 41). Tree swallows are widely distributed across North America and breed in a variety of environments. These populations were chosen to allow for comparisons among populations breeding in environments with different degrees of environmental predictability and reproductive value. Populations at higher latitude (Alaska) or elevation (Wyoming) are expected to experience cooler and more unpredictable weather conditions and a shorter breeding season starting later in the year (late May). In Tennessee, near the Southern edge of the breeding distribution, tree swallows are expected to experience warmer and more predictable weather conditions and a long breeding season with the first egg usually laid earlier in the season (early to mid-April). In New York, environmental conditions and breeding season length are expected to be intermediate, with the first egg usually laid in early May.

### General field methods and stress manipulation

In the four populations, nests were monitored every 1–2 days throughout the breeding season from the initiation of activity at each site to fledging, except for the last week of nestlings’ development (to avoid inducing premature fledging). For every active nest, we recorded clutch initiation date and completion dates, clutch size, hatch date, brood size, and the number of nestlings fledged. Nestling fates were determined by checking boxes 22–24 days after hatching as fledging usually occurs around day 20. We installed radio frequency identification (RFID) units on each box on the fourth day of incubation (see below). Birds were captured at their nest boxes by hand or using a manually activated trap. All birds were captured and sampled on specific days of life history substages, and during a set time of day, to reduce the variation in circulating glucocorticoid hormones resulting from circadian rhythms. Adult females were captured between 0700 and 1000 h in NY, TN and WY and between or 0600 and 0900 h in AK to compensate for the earlier start of activity due to the increased day length compared to the other populations.

Females were initially captured 6 or 7 days after clutch completion (capture number 1). At this capture, we took a first blood sample within 3 min of initial disturbance to measure baseline circulating corticosterone levels. A second blood sample was taken after 30 min of restraint in a cloth bag to measure stress-induced corticosterone levels. Immediately after this sample was taken, females were injected with dexamethasone (dex) (0.5 μl g^−1^, Dexamethasone Sodium Phosphate, Mylan Institutional LLC), a synthetic glucocorticoid that binds to receptors within the HPA axis, in order to assess negative feedback indepently of the stress-induced corticosterone level^29^. A final blood sample was taken 30 min after dex injection to measure the degree of down-regulation in circulating corticosterone (a measure of negative feedback). Between samples, we weighed the females, and measured the length of their skull from the back of the head to the bill tip (head-bill) and flattened wing length. Non-banded individuals received USGS leg bands and a celluloid colour band with attached passive integrated transponder (PIT) tag encoding a 10-digit hexadecimal string (Cyntag, Cynthiana, KY). Female age was determined based on plumage coloration and characterized as second year (SY) or after second year (ASY)^57^.

As part of a separate study, at their first capture, adult females were allocated to one of the experimental groups: control, feather restraint (in which three primaries were reversibly attached to alter flight ability and thereby increasing the cost of foraging), or predator exposure (see^29^ for details on both experimental treatments). Treatments started after the first capture and lasted for 5–6 days. As these treatments had no effects on HPA axis regulation (Table S1, S2, S3), data from all individuals were included in analyses.

Females were then recaptured 5–6 days later (on incubation day 12 or 13; capture 2), at the end of the experimental treatments (see above). At this capture, we only took a baseline blood sample and weighed the bird before release. Finally, we recaptured females again 6–8 days after eggs hatched (capture 3). We followed the same procedure as in capture 1, taking a baseline, restraint stress-induced, and post-dex blood samples, and again weighed each female.

Twelve days after eggs hatched, each nestling received an USGS leg band, was weighed and had head-bill and flat wing length measured.

All blood samples were collected from the alar vein, in heparinised microhematocrit capillary tubes. Blood samples were then transferred to microcentrifuge tubes, and kept on ice until centrifugation (within 4 h). After separation, the plasma was stored at − 20 °C in the field and then at − 80 °C in the lab until analysis. All methods were approved by Cornell IACUC and conducted with appropriate state and federal permits. We followed the guidelines to the use of wild birds in research of the Onithological Council for the care and use of animals.

### Provisioning behaviour

Number of feeding trips for females from nestling ages 1–18 was recorded using radio-frequency identification (RFID) devices (Cellular Tracking Technologies; Rio Grande, NJ, USA)^58^. RFID units were installed on each active box on day 4 of incubation. Antennae were fastened around each entrance hole so that birds had to pass directly through an antenna to enter or exit the box. We programmed our RFID units to sample for PIT tags every second between 0500 and 2200 h each day as tree swallows are not very active at night. Poll time was set at 500, and cycle time at 1,000. The delay time (minimum period of time between successive tag recordings) was set to 1 s. RFID boards were powered by 12 V 5Ah (PS-1250, PowerSonic, San Diego, CA, USA) batteries that were replaced every five days. At the first capture, each bird was fitted with a PIT tag attached to a colour band. Each PIT tag encoded a unique 10-digit hexadecimal string that was recorded, along with a time stamp, when birds passed through or perched on the antenna (see^59^ for more details). From the raw RFID records, we determined the number of daily feeding trips for each female through 18 days of age for the brood using an algorithm validated in the New York population^59^.

### Corticosterone assay

Steroids were extracted from plasma samples using a triple ethyl acetate extraction and then corticosterone levels were determined using an enzyme immunoassay kit (DetectX Corticosterone, Arbor Assays: K014-H5) previously validated for tree swallows^60^. Samples were run in duplicate and all samples from an individual were run on the same plate. In total we ran 47 assays with an average extraction efficiency of 92.8% and a detection limit of 0.47 ng ml^−1^ (calculated as described in^60^). The intra-assay variation based on duplicate samples was 8.88% and the inter-assay variation based on plasma pool run across plates was 11.1%.

### Data analysis

To characterise the degree of environmental unpredictability at the different field sites we calculated the unpredictability of temperature variables. We obtained historical weather data for each site over as long a yearly range as possible. For New York we obtained data from the North East Climate Center (https://www.nrcc.cornell.edu/) for the Game Farm road weather station (from 1983, located about 7 km from field sites) and from the Western Regional Climate Center (https://wrcc.dri.edu/) for the Prentice Cooper State Forest station in Tennessee (from 2003, located about 16 km from field sites), the May Creek station in Alaska (from 1990 located about 19 km from field sites) and the Burgess station in Wyoming (from 1992, located about 5 km from field sites). From these data, we extracted the average daily temperature, the daytime average daily temperature (between 0600 and 2200) which is the period when the swallows are the most active and the daily maximum temperature, which is known to affect flying insects’ activity and therefore food availability^61^.

We quantified the unpredictability of these temperatures variables during the breeding season: from April to June in Tennessee and New York and from May to July in Alaska and Wyoming. We calculated unpredictability using a general additive model (GAM) following the methods described in Franch-Gras et al.^43^. For each site, these variables were divided by their mean to normalise them before analysis^43^. This model (one for each site) considers the dispersion of the data in the time series around a typical curve of the normalised variable. For each weather variable and site, the typical curve was fitted in a GAM model in relation to the day of the year using the gam function in the mgcv package in R 3.5.3 (R Core Team, 2019). As suggested by Franch-Gras and collaborators^43^, we fitted the GAMs using cubic splines as smoothing function to not a priori constrain the shape of the curve. The standard deviation of the residuals of the fitted model (SD_res_) represents an index of unpredictability for each variable^43^. This index gives an overall measure of unpredictability using the historical records and is not intended to indicate variation in weather in the particular years of study at each site.

All data come from the first nesting attempt of each female. We compared females’ corticosterone levels by fitting a generalised linear mixed model (GLMM) with a gamma distribution that included population, life history substage, sample (baseline, stress-induced and post-dex), female age, treatment and their interactions (2-, 3-, 4- and 5-ways that include sample) as fixed factors, relative clutch initiation date as a covariate, female identity and experimental year as random intercepts, and population as a random slope. We further characterised females’ corticosterone regulation by calculating their glucocorticoid stress response as the difference between stress-induced and baseline corticosterone levels and their negative feedback as the difference between post-dex and stress-induced corticosterone levels. As it is not clear yet which trait is more important for the regulation of the phenotype—the raw hormone level or the response (the derived value)—we ran similar models on stress-induced and post-dex corticosterone values with baseline and stress-induced corticosterone levels as covariate respectively. For negative feedback, we also ran a model on the percentage change in corticosterone between the stress-induced and post-dex sampling periods. In order to directly compare the strength of the relationship between temperature unpredictability or reproductive value and females’ corticosterone regulation, we ran the same models but we replaced the fixed effect of population with either average temperature unpredictability or total breeding season length as a continuous variable. Then we used an information theory approach by comparing models’ Akaike Information Criterion (AIC) scores to determine which model, and thus which of these two variables, better predicts the observed variation in corticosterone regulation. We compared the magnitude of females’ acute stress response and negative feedback strength using GLMMs fit with a normal distribution including population, capture number, female age and their interactions as fixed factors, relative clutch initiation date as a covariate, female identity and experimental year as random intercepts, and population as a random slope. Population was added as a random slope in order to allow changes in HPA axis regulation to be different between populations. Within each population, we determined whether corticosterone level at each time point, and stress response and negative feedback were correlated using Pearson correlations.

We calculated the total breeding season length during the experimental year as the number of days between the first clutch initiation and the last day nestlings fledged at each site. We compared populations using a GLM fitted with a Poisson distribution. We also compared clutch size, brood size, hatching success and fledging success between populations. Population, female age and their interactions were added as fixed factors, and relative clutch initiation day as a covariate. The model for relative clutch initiation date was fitted with a normal distribution, models for clutch size and brood size with a Poisson distribution and models for hatching success and fledging success with a binomial distribution.

We compared the number of daily feeding trips females made using a generalised linear mixed model GLMM fitted with a Poisson distribution. Population, female age, treatment, their interactions and nestling age were added as fixed factors and brood size at each nestling age as a covariate. We also added relative clutch initiation date and brood size as covariates and nest identity as random factor. We used GLMMs to compare nestlings’ body mass with population, female age and their interaction as fixed factors. Female identity was added as a random factor.

GLMs were run using the GENMOD procedure and GLMMs the GLIMMIX procedure in SAS University Edition (SAS Institute Inc., Cary, NC, USA). Distributions for the different models were chosen depending of the type of data. Poisson distribution was used for counting data (nestling provisioning, breeding season length, clutch size, brood size), binomial distribution was used for binary data (0/1: hatching and fledging success), gamma distribution was used for continuous data when all data were superior to 0 (corticosterone levels, stress response, nestlings’ body mass) and Gaussian distribution was used for continuous data that included positive and negative values, after checking for normality of the residuals (negative feedback). Post‐hoc comparisons were performed using Tukey‐Kramer multiple comparison adjustment to obtain corrected p‐values. Probability levels < 0.05 were considered significant. Data are presented as mean ± SE. Figures 1, 2 and 4 were made in R 3.6.2 (R Core Team 2019, Vienna, Austria) and Fig. 3 in sigmaplot 14 (Systat Software, Inc, SanJose, CA, USA).

## Supplementary information

Supplementary Information.

## Data Availability

The datasets generated during and/or analysed during the current study are available from the corresponding author on reasonable request and will be deposited on a data repository upon acceptance for publication.
