# Supplementary material for: Environmental unpredictability shapes glucocorticoid regulation across populations of tree swallows
Source: Sci Rep. 2020 Aug 13;10:13682. doi: 10.1038/s41598-020-70161-4 (PMC7426823; doi:10.1038/s41598-020-70161-4)
Supplement: Supplementary file 1 — Supplementary Information. [file 41598_2020_70161_MOESM1_ESM.docx]

**Supplementary material**

Environmental unpredictability shapes glucocorticoid regulation across populations of tree swallows

Cedric Zimmer^1^, Conor C. Taff^1,2^, Daniel R. Ardia^3^, Alexandra P. Rose^4^, David A. Aborn^5^, L. Scott Johnson^6^ and Maren N. Vitousek^1,2^

^1^ Department of Ecology and Evolutionary Biology, Cornell University, Ithaca, NY 14853, USA

^2^ Cornell Lab of Ornithology, Ithaca, NY 14850, USA

^3^ Department of Biology, Franklin and Marshall College, Lancaster, PA 17604, USA

^4^ Institute of Arctic and Alpine Research, University of Colorado, Boulder, CO 80303, USA

^5^ Biology, Geology and Environmental Science, The University of Tennessee Chattanooga, Chattanooga, TN 37403, USA

^6^ Department of Biological Sciences, Towson University, Towson, MD 21252, USA

**Methods**

Experimental treatment

Females were randomly assigned to one of the three treatments before first (capture: 1): 1) A control (C) group where females were not exposed to experimental stressors after capture and handling procedures (NY: n = 16, TN: n = 22, AK: n = 23, WY: = 35); 2) A group where predation risk (P) was manipulated by exposing incubating females to a mink mount at random time once per day for 30 sec for 5 days after initial capture (NY: n = 13, TN: n = 25, AK: n = 23, WY: = 42); 3) A group where we simulated the effects of a cold snap (increased workload to get food) by increasing the costs of locomotion by securing together wing feathers. This Feather Restraint (FR) treatment was the same as the Low Feather Restraint treatment in NY (Zimmer et al. 2019) where primary feathers 4, 5 and 6 were attached using miniature zip-ties (NY: n = 13, TN: n = 26, AK: n = 23). Zip-ties were removed at the second capture (five days after the start of treatment) allowing them to regain their original flight efficiency. Females in this group that abandoned their nests during incubation, and were thus not recaptured, lost the zip-ties during molt before migration when primary feathers are replaced. The Feather Restraint treatment was stopped in Wyoming very early in the experiment because of an unusually long bout of cold and wet weather that resulted in high rates of nest abandonment (more than 50% of the females abandoned). Because this treatment was designed to simulate a cold snap, we chose not to continue deploying it during a naturally occurring cold snap to avoid unnaturally elevated abandonment rates.

*Data analysis*

We compared female body mass between the populations and treatments over the three captures using a GLMM with population, treatment, capture number, female age, and their interactions as fixed factors. We specified relative clutch initiation date as a covariate and female identity as a random factor. The model was fitted with a gamma distribution.

For each nestling, we calculated their scaled body mass based on wing length (SBM_wing_) as a measure of nestlings’ condition (Peig and Green, 2009). This provides an index of wing loading. We used a GLMM with population, female age, and their interaction as fixed factors. Female identity was added as a random factor.

We compared clutch initiation date between populations using GLMs with population, female age, and their interaction as fixed factors. Also using GLMs, we compared clutch size, the duration of incubation, brood size at hatching and the number of nestlings fledged between populations. Population, female age and their interactions were added as fixed factors, and relative clutch initiation day as a covariate. The model for clutch initiation date was fitted with a gamma distribution, and models for clutch size, incubation duration, brood size at hatching and number of nestlings fledged were fitted with a Poisson distribution.

GLMs were run using the GENMOD procedure and GLMMs the GLIMMIX procedure in SAS University Edition (SAS Institute Inc., Cary, NC). Post‐hoc comparisons were performed using Tukey‐Kramer multiple comparison adjustment to obtain corrected p‐values. Probability levels <0.05 were considered significant. Data are presented as mean ± SE.

**Results**

*Females’ phenotype*

Within each of the four populations, baseline corticosterone levels at both the first and third captures were significantly lower than stress-induced and post-dex corticosterone levels (t ≥ 4.28, p ≤ 0.006; Fig 3). In addition, stress-induced corticosterone levels were significantly higher than the post-dex levels (t ≥ 3.73, p ≤ 0.048; Fig 3).

Table S1: Results of the generalized linear mixed model for females corticosterone levels. The model was fitted with a gamma law and individual and experimental year specified as random factor and populations as random slope. Significant effects are in bold.

| Effect | DF | F-Value | P-Value |
| --- | --- | --- | --- |
| **Population** | **3,292** | **16.59** | **<.0001** |
| Age | 1,498.3 | 0.41 | 0.5201 |
| **Life history substage** | **2,1228** | **23.48** | **<.0001** |
| **Sample** | **2,1124** | **1197.93** | **<.0001** |
| Treatment | 2,474.2 | 0.41 | 0.6609 |
| Relative clutch initiation | 1,366 | 2.70 | 0.1014 |
| **Mass** | **1,826** | **73.17** | **<.0001** |
| **Population*Sample** | **6,1127** | **90** | **<.0001** |
| Age*Sample | 2,1125 | 1.23 | 0.2929 |
| Treatment*Sample | 4,1126 | 0.40 | 0.8062 |
| **Life history substage *Sample** | **2,1124** | **3.01** | **0.0498** |
| Population*Age*Sample | 9,888.7 | 1.89 | 0.0504 |
| Population*Treatment*Sample | 15,959.1 | 1.15 | 0.3087 |
| Treatment*Age*Sample | 6,1067 | 1.03 | 0.4031 |
| Treatment* Life history substage *Sample | 8,1146 | 1.69 | 0.0955 |
| **Population* Life history substage *Sample** | **12,1157** | **3.36** | **<.0001** |
| Age* Life history substage *Sample | 4,1149 | 1.11 | 0.3491 |
| Population*Age* Life history substage *Sample | 12,1152 | 1.23 | 0.2573 |
| Population*Treatment*Age*Sample | 15,982.3 | 1.10 | 0.3485 |
| Treatment*Age* Life history substage *Sample | 8,1145 | 0.82 | 0.5868 |
| Population*Treatment*Age* Life history substage *Sample | 40,1148 | 1.07 | 0.3607 |

Random effect variance: Individual (intercept) = 0.044, Individual (Population) = 0.012, Experimental Year (intercept) = 0.028, Residual = 0.44

Life history substage: mid-incubation, end of incubation, nestling provisioning

During mid-incubation, females’ baseline and stress-induced corticosterone levels in TN were significantly lower than in WY and AK (t ≥ 4.08, p ≤ 0.014; Fig 3). They were also lower in NY than in WY (t ≥ 3.86, p ≤ 0.031; Fig 1) but they did not differ between NY and AK (t ≤ 3.00, p ≥ 0.36; Fig 3) and between WY and AK (t ≤ 3.13, p ≥ 0.27; Fig 3). Post-dex corticosterone level was lower in TN than in WY (t = 3.96, p = 0.022; Fig 3) but did not differ between NY, WY and AK (t ≤ 3.14, p ≥ 0.26; Fig 3).

At the end of incubation, baseline corticosterone was lower in TN than in the 3 other populations (t ≥ 3.67, p ≤ 0.048; Fig 3) but did not differ between NY, WY and AK (t ≤ 1.41, p ≥ 0.98; Fig 3).

During nestling provisioning, baseline corticosterone levels were lower in TN than NY and AK (t ≥ 5.34, p < 0.0001; Fig 3) but levels in TN did not differ from those WY (t = 2.69, p = 0.61; Fig 3). In WY, levels were lower than in NY (t = 4.54, p = 0.002; Fig 3) but did not differ from those in AK (t = 2.02, p = 0.96; Fig 3). Baseline corticosterone level did not differ between NY and AK (t = 1.97, p = 0.97; Fig 3). Stress-induced corticosterone levels in TN were significantly lower than in AK (t = 4.61, p = 0.002; Fig 3) but did not differ from those in NY and WY (t ≤ 1.46, p ≥ 0.99; Fig 3). Levels in NY, WY and AK did not differ from one another (t ≤ 2.73, p ≥ 0.57; Fig 3). Post-dex corticosterone level did not differ between the 4 populations (t ≤ 1.73, p ≥ 0.99; Fig 3).

In Tennessee, baseline, stress-induced and post-dex corticosterone did not change between captures (t ≤ 1.68, p ≥ 0.98; Fig 3). In New York, baseline corticosterone did not differ between mid and end of incubation (t = 1.96, p = 0.98; Fig 3) and between end of incubation and nestling provisioning (t = 2.40, p = 0.82; Fig 3) but was higher during nestling provisioning than during mid-incubation (t = 4.35, p = 0.005; Fig 3). In Wyoming, baseline corticosterone did not differ between mid and end of incubation (t = 3.20, p = 0.23; Fig 3) and between end of incubation and nestling provisioning (t = 0.39, p = 1.00; Fig 3) but was lower during nestling provisioning than during mid-incubation (t = 3.73, p = 0.05; Fig 3). Stress-induced corticosterone level decreased between incubation and nestling provisioning (t = 5.26, p < 0.0001; Fig 3) while post-dex level did not change (t = 1.42, p = 0.99; Fig 3). In Alaska, baseline, stress-induced and post-dex corticosterone did not change between captures (t ≤ 1.71, p ≥ 0.99; Fig 3).

In the models where population was replaced by average temperature unpredictability or total breeding season length influence baseline, stress-induced and post-dex glucocorticoid levels (Unpredictability*Sample, Breeding season length*Sample; Tables S2, S3). Overall, baseline and stress- induced glucocorticoid levels increase with increasing temperature unpredictability (Fig S1) while they decrease with increasing breeding season length (Fig S1). Post-dex glucocorticoid levels covary with both temperature unpredictability and total breeding season (Fig S1).

Table S2: Results of the generalized linear mixed model for females corticosterone levels with average temperature unpredictability as continuous predictor. The model was fitted with a gamma law and individual and experimental year specified as random factor. Significant effects are in bold.

| Effect | | DF | | F-Value | | P-Value | |
| --- | --- | --- | --- | --- | --- | --- | --- |
| **Unpredictability** | **1,280.3** | | **18.96** | | **<.0001** | |  |
| Age | 1,195.3 | | 1.51 | | 0.2208 | |  |
| Life history substage | 2,1278 | | 1.27 | | 0.2817 | |  |
| **Sample** | **2,1149** | | **173.23** | | **<.0001** | |  |
| Treatment | 2,187.5 | | 0.42 | | 0.6577 | |  |
| Relative clutch initiation | 1,257.9 | | 6.36 | | 0.0123 | |  |
| **Mass** | **1,625.9** | | **56.97** | | **<.0001** | |  |
| **Unpredictability*Sample** | **2,1153** | | **12.59** | | **<.0001** | |  |
| Age*Sample | 2,1152 | | 2.71 | | 0.0669 | |  |
| Treatment*Sample | 4,1152 | | 0.82 | | 0.5149 | |  |
| Life history substage *Sample | 2,1149 | | 1.1 | | 0.3327 | |  |
| Unpredictability*Age*Sample | 3,747.1 | | 1.77 | | 0.1505 | |  |
| Unpredictability*Treatment*Sample | 6,822.3 | | 1.13 | | 0.3419 | |  |
| Treatment*Age*Sample | 6,720.7 | | 1.2 | | 0.3046 | |  |
| Treatment* Life history substage *Sample | 8,1189 | | 0.41 | | 0.9128 | |  |
| Unpredictability* Life history substage *Sample | 4,1212 | | 0.87 | | 0.4783 | |  |
| Age* Life history substage *Sample | 4,1191 | | 1.14 | | 0.3353 | |  |
| Unpredictability*Age* Life history substage *Sample | 4,1200 | | 0.85 | | 0.4956 | |  |
| Unpredictability*Treatment*Age*Sample | 6,818.8 | | 1.28 | | 0.2635 | |  |
| Treatment*Age* Life history substage *Sample | 8,1190 | | 1.03 | | 0.4085 | |  |
| Unpredictability*Treatment*Age* Life history substage *Sample | 16,1196 | | 1.11 | | 0.3445 | |  |

Random effect variance: Individual (intercept) = 0.050, Experimental Year (intercept) = 0.033, Residual = 0.478

AIC = 3149.87

Life history substage: mid-incubation, end of incubation, nestling provisioning

Table S3: Results of the generalized linear mixed model for females corticosterone levels with total breeding season length as a continuous predictor. The model was fitted with a gamma distribution, with individual and experimental year specified as random factors. Significant effects are in bold.

| Effect | DF | F-Value | P-Value |
| --- | --- | --- | --- |
| **Breeding Season Length** | **1,268.1** | **29.72** | **<.0001** |
| Age | 1,288.3 | 0.58 | 0.4453 |
| Life history substage | 2,1246 | 1.38 | 0.2521 |
| **Sample** | **2,1151** | **28.35** | **<.0001** |
| Treatment | 2,265.5 | 0.35 | 0.703 |
| **Relative clutch initiation** | **1,262.1** | **4.29** | **0.0393** |
| **Mass** | **1,579.1** | **77.56** | **<.0001** |
| **Breeding Season Length*Sample** | **2,1154** | **14.8** | **<.0001** |
| Age*Sample | 2,1155 | 1.61 | 0.1998 |
| Treatment*Sample | 4,1155 | 1.07 | 0.3693 |
| Life history substage *Sample | 2,1151 | 1.87 | 0.1549 |
| Breeding Season Length*Age*Sample | 3,749.6 | 1.61 | 0.1862 |
| Breeding Season Length*Treatment*Sample | 6,837.9 | 0.74 | 0.6157 |
| Treatment*Age*Sample | 6,909.6 | 1.27 | 0.2707 |
| Treatment* Life history substage *Sample | 8,1197 | 1.33 | 0.2243 |
| Breeding Season Length* Life history substage *Sample | 4,1208 | 1.16 | 0.3258 |
| Age* Life history substage *Sample | 4,1200 | 0.97 | 0.4209 |
| Breeding Season Length*Age* Life history substage *Sample | 4,1197 | 1.14 | 0.3367 |
| Breeding Season Length*Treatment*Age*Sample | 6,838.9 | 1.2 | 0.3035 |
| Treatment*Age* Life history substage *Sample | 8,1199 | 1.6 | 0.1203 |
| Breeding Season Length*Treatment*Age* Life history substage *Sample | 16,1193 | 1.24 | 0.2262 |

Random effect variance: Individual (intercept) = 0.051, Experimental Year (intercept) = 0.024, Residual = 0.474

AIC = 3531.98

Life history substage: mid-incubation, end of incubation, nestling provisioning





Figure S1: Changes in baseline, stress-induced and post-dex corticosterone levels with average temperature unpredictability and total breeding season length. Solid lines are regression lines and dashed lines are the 95% confidence interval.

The corticosterone stress response of females (difference between stress-induced and baseline corticosterone) differed between populations and captures (Table S4, p < 0.0001; Fig 4a). During mid-incubation, females in WY had a stronger stress response than females in the three other populations (t ≥ 5.08, p < 0.0001; Fig 4a). The stress response was stronger in AK than in TN and NY (t ≥ 2.71, p ≤ 0.044; Fig 3a) but did not differ between TN and NY (t = 1.47, p = 0.82; Fig 4a). During nestling provisioning, females in AK had a stronger stress response than females in the 3 other populations (t ≥ 3.01, p ≤ 0.048; Fig 4a) while the response did not differ between TN, NY and WY (t ≤ 0.96, p ≥ 0.97; Fig 4a). Within populations, stress response did not change between the two times that it was measured, i.e., during incubation and nestling provisioning (t ≤ 1.49, p ≥ 0.81; Fig 4a), except in WY where it decreased from one period to the next (t = 7.09, p < 0.0001; Fig 4a).

Table S4: Results of the generalized linear mixed model for the stress response of female tree swallows. The model was fitted with a gamma law. Individual and experimental year were specified as random factors and population as a random slope. Significant effects are in bold.

| Effect | DF | F-Value | P-Value |
| --- | --- | --- | --- |
| **Population** | **3,174** | **8.76** | **<.0001** |
| Age | 1,257.5 | 0.86 | 0.3557 |
| **Life history substage** | **1,311.5** | **51.06** | **<.0001** |
| Treatment | 2,210.1 | 0.16 | 0.85 |
| **Relative clutch initiation** | **1,269.8** | **3.95** | **0.0479** |
| **Mass** | **1,359.1** | **40.57** | **<.0001** |
| Population*Age | 3,168.1 | 0.58 | 0.6308 |
| **Population*Life history substage** | **3,236.7** | **5.49** | **0.0012** |
| Population*Treatment | 5,168.1 | 1.24 | 0.2932 |
| Age*Life history substage | 1,236.6 | 1.28 | 0.2595 |
| Treatment*Age | 2,209.4 | 0.31 | 0.7345 |
| Treatment*Life history substage | 2,229.5 | 0.72 | 0.4898 |
| Population*Age*Life history substage | 3,231 | 0.65 | 0.5842 |
| Population*Treatment*Age | 5,173.5 | 0.91 | 0.4773 |
| Treatment*Age*Life history substage | 2,229.4 | 1.97 | 0.1422 |
| Population*Treatment*Age*Life history substage | 10,226 | 0.97 | 0.4745 |

Random effect variance: Individual (intercept) = 0.059, Individual (Population) = 0.012, Experimental Year (intercept) = 0.102, Residual = 0.264

Life history substage: mid-incubation, nestling provisioning

Table S5: Results of the generalized linear mixed model for stress-induced corticosterone level of female tree swallows. The model was fitted with a gamma law. Individual and experimental year were specified as random factors and population as a random slope. Significant effects are in bold.

| Effect | DF | F Value | P-value |
| --- | --- | --- | --- |
| **Population** | **3,196.0** | **8.58** | **<0.0001** |
| Age | 1,317.9 | 0.06 | 0.8076 |
| **Life history substage** | **1,315.5** | **42.56** | **<.0001** |
| Treatment | 2,298.2 | 0.22 | 0.803 |
| **Mass** | **1,368.3** | **34.12** | **<.0001** |
| **Relative Clutch Initiation** | **1,276.5** | **9.09** | **0.0028** |
| Baseline | 1,362.3 | 1.77 | 0.1838 |
| Population*Age | 3,314.2 | 0.44 | 0.7239 |
| **Population*Life history substage** | **3,244.3** | **6.5** | **0.0003** |
| Population*Treatment | 5,294.9 | 0.43 | 0.8307 |
| Age*Life history substage | 1,243.2 | 0.73 | 0.3924 |
| Treatment*Life history substage | 2,239.8 | 1.13 | 0.3258 |
| Treatment*Age | 2,308.6 | 0.15 | 0.8638 |
| Population*Age*Life history substage | 3,237.4 | 0.55 | 0.6501 |
| Population*Treatment*Age | 5,308.5 | 1.01 | 0.4145 |
| Population*Treatment*Life history substage | 5,232.8 | 1.22 | 0.2999 |
| Treatment*Age*Life history substage | 2,237.3 | 1.44 | 0.2393 |
| Population*Treatment*Age*Life history substage | 5,234.0 | 0.45 | 0.8115 |

Random effect variance: Individual (intercept) = 0.051, Individual (Population) = 0.003, Experimental Year (intercept) = 0.209, Residual = 0.228

Life history substage: mid-incubation, nestling provisioning

Table S6: Results of the generalized linear mixed model for females’ stress response with average temperature unpredictability as a continuous predictor. The model was fitted with a gamma law. Individual and experimental year were specified as random factors. Significant effects are in bold.

| Effect | DF | F-Value | P-Value |
| --- | --- | --- | --- |
| **Unpredictability** | **1,257** | **13.59** | **0.0003** |
| Age | 1,270.4 | 1.4 | 0.2377 |
| **Life history substage** | **2,172** | **6.29** | **0.0127** |
| Treatment | 1,251.9 | 0.06 | 0.9460 |
| **Relative clutch initiation** | **1,383.6** | **4.37** | **0.0376** |
| **Mass** | **1,270.4** | **36.61** | **<.0001** |
| Unpredictability*Age | 1,215.6 | 1.56 | 0.2124 |
| Unpredictability*Life history substage | 1,239.1 | 0.24 | 0.6274 |
| Unpredictability*Treatment | 2,209 | 0.07 | 0.9331 |
| Age*Life history substage | 1,211.3 | 0.41 | 0.5229 |
| Treatment*Age | 2,170.5 | 1.67 | 0.1911 |
| Treatment*Life history substage | 2,226.3 | 0.59 | 0.5578 |
| Unpredictability*Age*Life history substage | 1,217.4 | 0.01 | 0.9332 |
| Unpredictability*Treatment*Age | 2,204.7 | 1.54 | 0.2160 |
| Treatment*Age*Life history substage | 2,226.3 | 2.05 | 0.1309 |

Random effect variance: Individual (intercept) = 0.094, Experimental Year (intercept) = 0.048, Residual = 0.292

AIC = 833.84

Life history substage: mid-incubation, nestling provisioning

Table S7: Results of the generalized linear mixed model for females stress response with total breeding season length as a continuous predictor. The model was fitted with a gamma law and individual and experimental year were specified as random factors. Significant effects are in bold.

| Effect | DF | F-Value | P-Value |
| --- | --- | --- | --- |
| **Breeding Season Length** | **1,249.9** | **16.08** | **<0.0001** |
| Age | 1,222.9 | 1.91 | 0.1682 |
| Life history substage | 1,221.1 | 0.00 | 0.954 |
| Treatment | 2,241.9 | 0.30 | 0.7413 |
| **Relative clutch initiation** | **1,256** | **3.90** | **0.0492** |
| **Mass** | **1,381.1** | **52.45** | **<.0001** |
| Breeding Season Length *Age | 1,213.9 | 2.05 | 0.1539 |
| Breeding Season Length *Life history substage | 1,231.3 | 2.00 | 0.159 |
| Breeding Season Length *Treatment | 2,218.8 | 0.28 | 0.7569 |
| Age*Life history substage | 1,216.5 | 0.09 | 0.7609 |
| Treatment*Age | 2,230.4 | 1.06 | 0.3494 |
| Treatment*Life history substage | 2,227.3 | 0.45 | 0.637 |
| Breeding Season Length *Age*Life history substage | 1,217.4 | 0.01 | 0.9332 |
| Breeding Season Length *Treatment*Age | 1,212.7 | 0.01 | 0.9082 |
| Treatment*Age*Life history substage | 2,212.7 | 1.22 | 0.2975 |

Random effect variance: Individual (intercept) = 0.076, Experimental Year (intercept) = 0.069, Residual = 0.289.

AIC = 947.01

Life history substage: mid-incubation, nestling provisioning





Figure S2: Changes in the stress response with average temperature unpredictability and total breeding season length.

Table S8: Results of the generalized linear mixed model for females’ negative feedback. The model was fitted with a normal law and individual and experimental year specified as random factor and populations as random slope. Significant effects are in bold.

| Effect | DF | F-Value | P-Value |
| --- | --- | --- | --- |
| **Population** | **3,171.4** | **4.01** | **0.0086** |
| Age | 1,248.1 | 0.07 | 0.7979 |
| Life history substage | 1,304 | 25.88 | <.0001 |
| Treatment | 2,209.1 | 0.04 | 0.9605 |
| **Relative clutch initiation** | **1,312.5** | **5.39** | **0.0209** |
| **Mass** | **1,331.1** | **12.09** | **0.0006** |
| Population*Age | 3,170.6 | 0.79 | 0.4993 |
| **Population*Life history substage** | **3,267** | **11.63** | **<.0001** |
| Population*Treatment | 5,173.3 | 0.51 | 0.7656 |
| Age*Life history substage | 1,273.2 | 2.44 | 0.1196 |
| Treatment*Age | 2,209 | 0.28 | 0.7568 |
| Treatment*Life history substage | 2,267.3 | 1.28 | 0.2799 |
| Population*Age*Life history substage | 3,265.2 | 0.71 | 0.5455 |
| Population*Treatment*Age | 5,183.5 | 0.53 | 0.7505 |
| Treatment*Age*Life history substage | 2,267.4 | 1.03 | 0.3581 |
| Population*Treatment*Age*Life history substage | 10,263.4 | 1.23 | 0.2694 |

Random effect variance: Individual (intercept) = 93.28, Individual (Population) = 45.37, Experimental Year (intercept) = 92.81, Residual = 357.77.

Life history substage: mid-incubation, nestling provisioning

Post-dex corticosterone level, accounting for stress-induced corticosterone level (β = 0.0076 [0.0054 – 0.010], F_1,313_ = 47.77, p < 0.0001; Table S9), differed between population and life history substages (population x life history substage: F_3,240.1_ = 3.51, p = 0.016; Table S9). During mid-incubation, post-dex corticosterone level corrected by stress-induced level was stronger in AK than in the three other populations (t ≥ 2.75, p ≤ 0.01). At this time period, post-dex corticosterone level corrected by stress-induced level did not differ between the three other populations (t ≤ 1.74, p ≥ 0.08). During the nestling provisioning period, females in AK had lower post-dex corticosterone level corrected by stress-induced level than females in TN and NY (t ≥ 1.89, p ≤ 0.05) but did not differ from females in WY (t = 0.97, p = 0.33). At this time, post-dex corticosterone level corrected by stress-induced level did not differ between TN, NY and WY (t ≤ 1.41, p ≥ 0.16). Post-dex corticosterone level corrected by stress-induced level differed between populations by age (population x life history substage: F_3,181.9_ = 3.35, p = 0.02; Table S9). Second year females in NY had higher post-dex corticosterone level corrected by stress-induced level than second year and after second year females in AK (t ≥ 3.26, p ≤ 0.03).

Table S9: Results of the generalized linear mixed model for post-dex corticosterone level of female tree swallows. The model was fitted with a gamma law. Individual and experimental year were specified as random factors and population as a random slope. Significant effects are in bold.

| Effect | DF | F Value | P-value |
| --- | --- | --- | --- |
| **Population** | **3,200.3** | **5.32** | **0.0015** |
| Age | 1,267.2 | 1.5 | 0.2215 |
| **Life history substage** | **1,325.2** | **4.16** | **0.0423** |
| Treatment | 2,224.8 | 1.26 | 0.2864 |
| **Mass** | **1,355.1** | **8.53** | **0.0037** |
| Relative Clutch Initiation | 1,270.5 | 0.02 | 0.8918 |
| **Stress-induced CORT** | **1,313** | **47.77** | **<.0001** |
| **Population*Age** | **3,181.9** | **3.35** | **0.0203** |
| **Population*Life history substage** | **3,240.1** | **3.51** | **0.0160** |
| Population*Treatment | 5,187.5 | 1.12 | 0.3535 |
| Age*Life history substage | 1,237.6 | 3.84 | 0.0514 |
| Treatment*Life history substage | 2,232.2 | 0.25 | 0.7781 |
| Treatment*Age | 2,223.1 | 1.91 | 0.1500 |
| Population*Age*Life history substage | 3,230.6 | 2.99 | 0.0318 |
| Population*Treatment*Age | 5,187.1 | 1.64 | 0.1504 |
| Population*Treatment*Life history substage | 5,227.9 | 0.35 | 0.8796 |
| Treatment*Age*Life history substage | 2,229.5 | 1.41 | 0.2471 |
| Population*Treatment*Age*Life history substage | 5,227.2 | 0.26 | 0.9334 |

Random effect variance: Individual (intercept) = 0.11, Individual (Population) = 0.03, Experimental Year (intercept) = 0.001, Residual = 0.22.

Life history substage: mid-incubation, nestling provisioning

Relative decrease in corticosterone between stress-induced and post-dex levels differed between populations and life history substages (population x life history substage: F_3,312.4_ = 4.94, p = 0.002; Table S10). During mid-incubation, negative feedback was stronger in WY (-69.8 ± 5.0) and AK (-59.8 ± 3.2) than than in TN (-40.0 ± 4.9) and NY (-51.9 ± 5.1) (t ≥ 2.06, p < 0.04). At this time period, negative feedback did not differ between WY and AK (t = 0.45, p = 0.65), and NY and TN (t = 0.79, p = 0.43). During the nestling provisioning period, females in AK (-63.1 ± 3.3) still had stronger negative feedback than females in all other populations (t ≥ 1.96, p ≤ 0.05); at this time point negative feedback did not differ among the other populations (WY: -44.4 ± 5.8, NY: -47.8 ± 8.3, TN: -33.2 ± 10.24; t ≤ 1.88, p ≥ 0.06). Relative decrease in corticosterone between stress-induced and post-dex levels differed between populations and captures and ages (population x age: F_3,172.4_ = 2.82, p = 0.04; Table S10). However, multiple comparisons did not show any significant differences.

Table S10: Results of the generalized linear mixed model for percentage of corticosterone change between stress-induced and post-dex samples in female tree swallows. The model was fitted with a normal law. Individual and experimental year were specified as random factors and population as a random slope. Significant effects are in bold.

| Effect | DF | F-Value | P-Value |
| --- | --- | --- | --- |
| **Population** | **3,187.9** | **3.45** | **0.0177** |
| Age | 1,227 | 0.03 | 0.8606 |
| **Life history substage** | **1,331.8** | **4.46** | **0.0354** |
| Treatment | 2,186.1 | 0.03 | 0.9680 |
| Relative clutch initiation | 1,352.3 | 2.86 | 0.0920 |
| Mass | 1,351.6 | 1.96 | 0.1623 |
| **Population*Age** | **3,172.4** | **2.82** | **0.0406** |
| **Population*Life history substage** | **3,312.4** | **4.94** | **0.0023** |
| Population*Treatment | 5,169.5 | 0.59 | 0.7083 |
| Age*Life history substage | 1,316.9 | 0.08 | 0.7722 |
| Treatment*Age | 2,186.3 | 1.27 | 0.2832 |
| Treatment*Life history substage | 2,314.9 | 0.07 | 0.9337 |
| Population*Age*Life history substage | 3,309 | 1.28 | 0.2805 |
| Population*Treatment*Age | 5,311.4 | 0.13 | 0.9854 |
| Treatment*Age*Life history substage | 5,170.1 | 0.6 | 0.7018 |
| Population*Treatment*Age*Life history substage | 2,315 | 0.7 | 0.4975 |

Random effect variance: Individual (intercept) = 1354.75, Individual (Population) = 152.62, Experimental Year (intercept) = 620.89, Residual = 1038.17.

Life history substage: mid-incubation, nestling provisioning

Table S11: Results of the generalized linear mixed model for females’ negative feedback with average temperature unpredictability as continuous predictor. The model was fitted with a gamma law and individual and experimental year specified as random factor. Significant effects are in bold.

| Effect | DF | F-Value | P-Value |
| --- | --- | --- | --- |
| **Unpredictability** | **1,300.8** | **8.33** | **0.0042** |
| Age | 1,310.4 | 1.61 | 0.2059 |
| **Life history substage** | **1,276.7** | **4.14** | **0.0429** |
| Treatment | 2,303.9 | 0.03 | 0.9704 |
| **Relative clutch initiation** | **1,325.9** | **5.55** | **0.0191** |
| **Mass** | **1,377.1** | **20.72** | **<.0001** |
| **Stress-induced corticosterone** | **1,371.3** | **239.27** | **<.0001** |
| Unpredictability*Age | 1,349.7 | 1.93 | 0.1657 |
| Unpredictability*Life history substage | 1,266.2 | 0.09 | 0.7613 |
| Unpredictability*Treatment | 2,319.5 | 0.10 | 0.9016 |
| Age*Life history substage | 1,242.3 | 0.06 | 0.8020 |
| Treatment*Age | 2,301.5 | 0.79 | 0.4547 |
| Treatment*Life history substage | 2,241.4 | 0.06 | 0.9381 |
| Unpredictability*Age*Life history substage | 1,257.7 | 0.86 | 0.3560 |
| Unpredictability*Treatment*Age | 2,338 | 0.70 | 0.4974 |
| Unpredictability*Treatment*Life history substage | 2,255 | 0.20 | 0.8220 |
| Treatment*Age*Life history substage | 2,242.5 | 1.14 | 0.3220 |
| Unpredictability*Treatment*Age*Life history substage | 2,256.1 | 1.07 | 0.3428 |

Random effect variance: Individual (intercept) = 112.85Experimental Year (intercept) = 4.17, Residual = 450.55.

AIC = 3525.71

Life history substage: mid-incubation, nestling provisioning

Table S12: Results of the generalized linear mixed model for females negative feedback with total breeding season length as a continuous predictor. The model was fitted with a gamma law and individual and experimental year specified as random factor. Significant effects are in bold.

| Effect | DF | F-Value | P-Value |
| --- | --- | --- | --- |
| **Breeding Season Length** | **1,277.8** | **6.54** | **0.0111** |
| Age | 1,286.6 | 1.91 | 0.1679 |
| Life history substage | 1,262.7 | 0.03 | 0.8731 |
| Treatment | 2,248.7 | 0.15 | 0.8607 |
| **Relative clutch initiation** | **1,335.8** | **6.91** | **0.009** |
| **Mass** | **1,383.6** | **29.69** | **<.0001** |
| **Stress-induced corticosterone** | **1,383** | **260.55** | **<.0001** |
| Breeding Season Length*Age | 1,262.4 | 2.01 | 0.1580 |
| Breeding Season Length*Life history substage | 2,296.5 | 0.09 | 0.9129 |
| Breeding Season Length*Treatment | 1,262.3 | 1.8 | 0.1805 |
| Age*Life history substage | 2,276.1 | 0.54 | 0.5816 |
| Treatment*Age | 2,259.5 | 0.2 | 0.8191 |
| Treatment*Life history substage | 1,252.6 | 0.97 | 0.3263 |
| Breeding Season Length*Age*Life history substage | 2,314.8 | 0.63 | 0.5333 |
| Breeding Season Length*Treatment*Age | 2,251 | 0.16 | 0.8534 |
| Breeding Season Length*Treatment*Life history substage | 2,260.2 | 1.08 | 0.3408 |
| Treatment*Age*Life history substage | 2,251.9 | 1.13 | 0.3235 |
| Breeding Season Length*Treatment*Age*Life history substage | 1,262.4 | 2.01 | 0.1580 |

Random effect variance: Individual (intercept) = 113.89, Experimental Year (intercept) = 3.36, Residual = 442.92.

AIC = 3641.82

Life history substage: mid-incubation, nestling provisioning





Figure S3: Changes in the negative feedback with average temperature unpredictability and total breeding season length.

Female body mass differed between populations; these differences were affected by capture number (population x capture number: F_6,397.5_ = 18.73, p < 0.0001; Fig S4). Females in WY were lighter than females in the 3 other populations at all captures (t ≥ 3.73, p ≤ 0.012; Fig S4). In TN, females were heavier than in the 3 other populations during mid-incubation (t ≥ 3.56, p ≤ 0.012; Fig S4) and their body mass decreased between each capture (t ≥ 8.95, p < 0.0001; Fig S4). In NY, body mass did not change between the first and second captures (t = 0.16, p = 1.00; Fig S4) but decreased between the second and third captures (t = 8.09, p < 0.0001; Fig S4). In WY, female body mass did not change captures (t ≤ 1.85, p ≥ 0.79; Fig S4). In AK, female body mass decreased between each capture (t ≥ 4.30, p ≤ 0.0007; Fig S4).





Figure S4: Body mass of females in Tennessee (orange), New York (red), Wyoming (purple), and Alaska (blue) at the first capture during mid incubation, the second capture at the end of incubation and the third capture during nestling provisioning. Different letters indicate significant differences.

*Breeding phenology, behavior, nestlings’ phenotype and success*

Because tree swallows breeding at higher latitudes arrive later to their breeding sites (Gow et al., 2019), populations differed in their clutch initiation dates (population: χ^2^_3,250_ = 480.43, p < 0.0001). The four populations differed from each other (z ≥ 3.06, p ≤ 0.012) with females in Tennessee (118 ± 1 day of the year) showing the earliest clutch initiation followed by females in New York (135 ± 1 day of the year), females in Wyoming (152 ± 1 day of the year), and finally females in Alaska (158 ± 1 day of the year). Across populations, second-year females initiated clutches later than after-second-year females (relative clutch initiation dates: 14.3 ± 0.8 days vs. 9.0 ± 0.5 days; χ^2^_1,250_ = 32.63, p < 0.0001).

Clutch size did not differ between populations (TN: 5.8 ± 0.1 eggs, NY: 5.4 ± 0.1 eggs, WY: 5.4 ± 0.1 eggs, AK: 5.2 ± 0.1 eggs; χ^2^_3,250_ = 5.90, p = 0.12). Clutch size decreased with increasing relative clutch initiation date across populations (β = -0.009 [-0.018 – -0.0004], χ^2^_3,250_ = 4.20, p = 0.041) but did not differ with age (χ^2^_1,250_ = 0.38, p = 0.54).

Incubation duration differed between populations (χ^2^_3,159_ = 9.01, p = 0.029). Incubation length was the shortest in TN (13.5 ± 0.1 days) and longest in WY (16.4 ± 0.3 days), a difference that was significant (z = 2.93, p = 0.018). Incubation duration was intermediate in NY (15.2 ± 0.2 days) and AK (14.5 ± 0.1 days); incubation length in NY and AK did not differ, nor did these sites differ from TN and WY (z ≤ 2.19, p ≥ 0.13).

Brood size at hatching (maximum brood size) did not differ between populations (TN: 4.7 ± 0.2 nestlings, NY: 4.3 ± 0.2 nestlings, WY: 4.4 ± 0.3 nestlings, AK: 4.4 ± 0.2 nestlings; χ^2^_3,160_ = 1.20, p = 0.75).

Nestling provisioning behavior — measured as the number of daily feeding trips to the nest made by females between day 1 and 18 — increased with increasing brood size (β = 0.041 [0.035 – 0.047], F_1,1933_ = 66.13, p < 0.0001). Number of feeding trips by females changed over the nestling rearing period (F_17,1933_ = 654.91, p < 0.0001, Fig. S5). Number of feeding trips generally increased until day 13 when the number of feeding trips reached its maximum before slowly decreasing until day 18 (Fig. S5).

Figure 5: Number of daily feeding trips for females in Tennessee (orange), New York (red), Wyoming (purple), Alaska (blue) over the nestling rearing period. Faded dots represent individual females’ data on each day. Bright dots represent average fitted value ± SD for each population for each nestlings’ day. Different letters indicate significant differences between days.

Nestling body mass on day 12 after hatching differed between populations (F_3,128.5_ = 4.02, p = 0.009). Nestlings in AK (21.2 ± 0.2 g) were significantly heavier than in TN (19.6 ± 0.2 g; t = 2.41, p = 0.018) and NY (18.7 ± 0.5 g; t = 2.65, p = 0.024) but did not differ from those in WY (20.6 ± 0.2 g; t = 1.11, p = 0.68).

Fledging success was higher for ASY females (79.2 %, 76 of 96) than for SY females (61.7 %, 50 of 81) (χ^2^_1,250_ = 5.60, p = 0.018). Scaled body mass, a measure of condition, was significantly lower in TN than in all other populations (F_3,130.5_ = 12.06, p < 0.0001; TN: 18.6 ± 0.2 g, NY: 22.7 ± 0.5 g, WY: 21.2 ± 0.2 g, AK: 21.5 ± 0.2 g; t ≥ 3.01, p ≤ 0.016). The number of nestlings fledged also differed between populations (χ^2^_3,250_ = 22.49, p < 0.0001) and age classes (χ^2^_1,250_ = 4.53, p = 0.033). The number of nestlings fledged per nest was lower in NY (1.1 ± 0.3 nestlings) than in the 3 other populations (TN: 3.2 ± 0.3, AK: 3.0 ± 0.3, WY: 2.9 ± 0.3; t ≥ 5.13, p < 0.0001) and greater in ASY (3.1 ± 0.2 nestlings) than in SY females (2.2 ± 0.2 nestlings).

Table S13: Average (±SE) historical hatching and fledging success in the four populations.

| Population | Years | Hatching success | Fledging success |
| --- | --- | --- | --- |
| Tennessee | 2014 - 2017 | 81.9 ± 16.6% | 88.6 ± 15.9% |
| New York | 2014 - 2019 | 80.6 ± 3.1% | 78.2 ± 1.9% |
| Wyoming* | 2019 | 95.90% | 91.50% |
| Alaska | 2016-2017 | 70.5 ± 6.2% | 81.2 ± 3.4% |

*Data for Wyoming come from only one year, as this population has not been monitored as extensively as the other populations. Weather conditions during the 2019 breeding season were better than in 2018 with no extended periods of cold and wet weather occurring during the middle of the breeding season. These data show that the Wyoming site is not an inherently poor site for reproduction and hence the low hatching success during the experimental year is likely due to the bad weather conditions during incubation.

**Discussion**

Hatching success and fledging success did not differ much between populations. The exception was that Wyoming females had lower hatching success and New York females had lower fledging success during the experimental year. These two populations experienced unusually bad weather conditions in the year of study during the incubation and nestling rearing periods, respectively. Thus, differences were likely the result of current conditions rather than inherent differences in the quality of these locations as breeding sites (Table S4). In support of this, previous studies at sites in Tennessee, New York and Alaska well as sites in Ontario and North Carolina did not find differences in the number of nestlings fledged (Ardia, 2007; Akçay et al., 2016).

Table S14: Parameter estimates, standard error and confidence interval for the generalized linear mixed model for females corticosterone levels. The model was fitted with a gamma law and individual and experimental year specified as random factor and populations as random slope. Spl = Sample, Base = Baseline, Str = stress-induced, dex = post-dex. LHSS = Life history substage, Mid Inc = Mid Incubation, End incubation = End incubation, Nestling = Nestling provisioning, Rel_CI = relative clutch initiation date, Mass = Body mass

| Effect | Population | Treatment | Age | Sample | LHSS | Estimate | SE | Lower | Upper |
| --- | --- | --- | --- | --- | --- | --- | --- | --- | --- |
|  |  |  |  |  |  |  |  |  |  |
| Intercept |  |  |  |  |  | 5.3033 | 0.4368 | 4.4463 | 6.1603 |
| Population | AK |  |  |  |  | 0.09901 | 0.3686 | -0.6243 | 0.8224 |
| Population | NY |  |  |  |  | -0.6954 | 0.4217 | -1.5229 | 0.1321 |
| Population | TN |  |  |  |  | 0.101 | 0.4021 | -0.688 | 0.8899 |
| Population | WY |  |  |  |  | 0 | . | . | . |
| Age |  |  | ASY |  |  | -0.1326 | 0.4025 | -0.9226 | 0.6575 |
| Age |  |  | SY |  |  | 0 | . | . | . |
| LHSS |  |  |  |  | Mid Inc | 0.01197 | 0.3204 | -0.6167 | 0.6406 |
| LHSS |  |  |  |  | End Inc | 0.5423 | 0.3539 | -0.152 | 1.2365 |
| LHSS |  |  |  |  | Nestling | 0 | . | . | . |
| Spl |  |  |  | Base |  | -2.4546 | 0.3767 | -3.1936 | -1.7155 |
| Spl |  |  |  | Str |  | 0.3897 | 0.3767 | -0.3493 | 1.1288 |
| Spl |  |  |  | Dex |  | 0 | . | . | . |
| Treatment |  | Control |  |  |  | -0.6406 | 0.4518 | -1.5274 | 0.2462 |
| Treatment |  | Low_Tape |  |  |  | -0.03431 | 0.3871 | -0.794 | 0.7254 |
| Treatment |  | Predator |  |  |  | 0 | . | . | . |
| Rel_CI |  |  |  |  |  | -0.00675 | 0.004109 | -0.01483 | 0.001333 |
| Mass |  |  |  |  |  | -0.1433 | 0.01676 | -0.1762 | -0.1105 |
| Population*Spl | AK |  |  | Base |  | 1.0704 | 0.4683 | 0.1516 | 1.9892 |
| Population*Spl | AK |  |  | Str |  | 0.8199 | 0.4683 | -0.09887 | 1.7387 |
| Population*Spl | AK |  |  | Dex |  | 0 | . | . | . |
| Population*Spl | NY |  |  | Base |  | 2.5615 | 0.5587 | 1.4653 | 3.6577 |
| Population*Spl | NY |  |  | Str |  | 0.8276 | 0.5587 | -0.2686 | 1.9238 |
| Population*Spl | NY |  |  | Dex |  | 0 | . | . | . |
| Population*Spl | TN |  |  | Base |  | 0.03382 | 0.5133 | -0.9734 | 1.041 |
| Population*Spl | TN |  |  | Str |  | 0.04045 | 0.5133 | -0.9667 | 1.0476 |
| Population*Spl | TN |  |  | Dex |  | 0 | . | . | . |
| Population*Spl | WY |  |  | Base |  | 0 | . | . | . |
| Population*Spl | WY |  |  | Str |  | 0 | . | . | . |
| Population*Spl | WY |  |  | Dex |  | 0 | . | . | . |
| Age*Spl |  |  | ASY | Base |  | 0.9231 | 0.4932 | -0.04459 | 1.8908 |
| Age*Spl |  |  | ASY | Str |  | 0.6011 | 0.5068 | -0.3934 | 1.5955 |
| Age*Spl |  |  | ASY | Dex |  | 0 | . | . | . |
| Age*Spl |  |  | SY | Base |  | 0 | . | . | . |
| Age*Spl |  |  | SY | Str |  | 0 | . | . | . |
| Age*Spl |  |  | SY | Dex |  | 0 | . | . | . |
| Treatment*Spl |  | Control |  | Base |  | 1.0894 | 0.5587 | -0.00676 | 2.1856 |
| Treatment*Spl |  | Control |  | Str |  | 0.8035 | 0.5587 | -0.2927 | 1.8997 |
| Treatment*Spl |  | Control |  | Dex |  | 0 | . | . | . |
| Treatment*Spl |  | Low_Tape |  | Base |  | 0.1466 | 0.5133 | -0.8606 | 1.1537 |
| Treatment*Spl |  | Low_Tape |  | Str |  | 0.05355 | 0.5133 | -0.9536 | 1.0607 |
| Treatment*Spl |  | Low_Tape |  | Dex |  | 0 | . | . | . |
| Treatment*Spl |  | Predator |  | Base |  | 0 | . | . | . |
| Treatment*Spl |  | Predator |  | Str |  | 0 | . | . | . |
| Treatment*Spl |  | Predator |  | Dex |  | 0 | . | . | . |
| LHSS*Spl |  |  |  | Base | Mid Inc | 0.6849 | 0.4321 | -0.1629 | 1.5326 |
| LHSS*Spl |  |  |  | Str | Mid Inc | 0.8702 | 0.4321 | 0.02248 | 1.718 |
| LHSS*Spl |  |  |  | Dex | Mid Inc | 0 | . | . | . |
| LHSS*Spl |  |  |  | Base | End Inc | 0 | . | . | . |
| LHSS*Spl |  |  |  | Base | Nestling | 0 | . | . | . |
| LHSS*Spl |  |  |  | Str | Nestling | 0 | . | . | . |
| LHSS*Spl |  |  |  | Dex | Nestling | 0 | . | . | . |
| Population*Age*Spl | AK |  | ASY | Base |  | -1.4254 | 0.4957 | -2.3982 | -0.4527 |
| Population*Age*Spl | AK |  | ASY | Str |  | -0.6256 | 0.511 | -1.6283 | 0.377 |
| Population*Age*Spl | AK |  | ASY | Dex |  | -0.00241 | 0.5026 | -0.9885 | 0.9837 |
| Population*Age*Spl | AK |  | SY | Base |  | 0 | . | . | . |
| Population*Age*Spl | AK |  | SY | Str |  | 0 | . | . | . |
| Population*Age*Spl | AK |  | SY | Dex |  | 0 | . | . | . |
| Population*Age*Spl | NY |  | ASY | Base |  | -1.1938 | 0.5891 | -2.3496 | -0.03799 |
| Population*Age*Spl | NY |  | ASY | Str |  | -0.3253 | 0.602 | -1.5064 | 0.8558 |
| Population*Age*Spl | NY |  | ASY | Dex |  | 0.5926 | 0.5948 | -0.5745 | 1.7596 |
| Population*Age*Spl | NY |  | SY | Base |  | 0 | . | . | . |
| Population*Age*Spl | NY |  | SY | Str |  | 0 | . | . | . |
| Population*Age*Spl | NY |  | SY | Dex |  | 0 | . | . | . |
| Population*Age*Spl | TN |  | ASY | Base |  | -0.9576 | 0.5233 | -1.9843 | 0.06922 |
| Population*Age*Spl | TN |  | ASY | Str |  | -0.5636 | 0.5378 | -1.6188 | 0.4917 |
| Population*Age*Spl | TN |  | ASY | Dex |  | -0.09331 | 0.5298 | -1.1329 | 0.9463 |
| Population*Age*Spl | TN |  | SY | Base |  | 0 | . | . | . |
| Population*Age*Spl | TN |  | SY | Str |  | 0 | . | . | . |
| Population*Age*Spl | TN |  | SY | Dex |  | 0 | . | . | . |
| Population*Age*Spl | WY |  | ASY | Base |  | 0 | . | . | . |
| Population*Age*Spl | WY |  | ASY | Str |  | 0 | . | . | . |
| Population*Age*Spl | WY |  | ASY | Dex |  | 0 | . | . | . |
| Population*Age*Spl | WY |  | SY | Base |  | 0 | . | . | . |
| Population*Age*Spl | WY |  | SY | Str |  | 0 | . | . | . |
| Population*Age*Spl | WY |  | SY | Dex |  | 0 | . | . | . |
| Population*Treatment*Spl | AK | Control |  | Base |  | -1.0053 | 0.5493 | -2.0831 | 0.07245 |
| Population*Treatment*Spl | AK | Control |  | Str |  | -0.0337 | 0.5493 | -1.1115 | 1.0441 |
| Population*Treatment*Spl | AK | Control |  | Dex |  | 0.5246 | 0.5493 | -0.5532 | 1.6024 |
| Population*Treatment*Spl | AK | Low_Tape |  | Base |  | -0.4339 | 0.5577 | -1.5282 | 0.6604 |
| Population*Treatment*Spl | AK | Low_Tape |  | Str |  | -0.6685 | 0.5577 | -1.7628 | 0.4258 |
| Population*Treatment*Spl | AK | Low_Tape |  | Dex |  | -0.6467 | 0.5577 | -1.7411 | 0.4476 |
| Population*Treatment*Spl | AK | Predator |  | Base |  | 0 | . | . | . |
| Population*Treatment*Spl | AK | Predator |  | Str |  | 0 | . | . | . |
| Population*Treatment*Spl | AK | Predator |  | Dex |  | 0 | . | . | . |
| Population*Treatment*Spl | NY | Control |  | Base |  | -1.1449 | 0.6321 | -2.3851 | 0.09541 |
| Population*Treatment*Spl | NY | Control |  | Str |  | 0.1763 | 0.6321 | -1.064 | 1.4165 |
| Population*Treatment*Spl | NY | Control |  | Dex |  | 0.9014 | 0.6321 | -0.3389 | 2.1417 |
| Population*Treatment*Spl | NY | Low_Tape |  | Base |  | -1.2862 | 0.8131 | -2.8816 | 0.3091 |
| Population*Treatment*Spl | NY | Low_Tape |  | Str |  | 0.3736 | 0.8131 | -1.2217 | 1.9689 |
| Population*Treatment*Spl | NY | Low_Tape |  | Dex |  | -0.3942 | 0.8131 | -1.9895 | 1.2011 |
| Population*Treatment*Spl | NY | Predator |  | Base |  | 0 | . | . | . |
| Population*Treatment*Spl | NY | Predator |  | Str |  | 0 | . | . | . |
| Population*Treatment*Spl | NY | Predator |  | Dex |  | 0 | . | . | . |
| Population*Treatment*Spl | TN | Control |  | Base |  | -0.4768 | 0.5649 | -1.5853 | 0.6318 |
| Population*Treatment*Spl | TN | Control |  | Str |  | -0.4843 | 0.5649 | -1.5929 | 0.6242 |
| Population*Treatment*Spl | TN | Control |  | Dex |  | 0.4598 | 0.5649 | -0.6488 | 1.5683 |
| Population*Treatment*Spl | TN | Low_Tape |  | Base |  | 0 | . | . | . |
| Population*Treatment*Spl | TN | Low_Tape |  | Str |  | 0 | . | . | . |
| Population*Treatment*Spl | TN | Low_Tape |  | Dex |  | 0 | . | . | . |
| Population*Treatment*Spl | TN | Predator |  | Base |  | 0 | . | . | . |
| Population*Treatment*Spl | TN | Predator |  | Str |  | 0 | . | . | . |
| Population*Treatment*Spl | TN | Predator |  | Dex |  | 0 | . | . | . |
| Population*Treatment*Spl | WY | Control |  | Base |  | 0 | . | . | . |
| Population*Treatment*Spl | WY | Control |  | Str |  | 0 | . | . | . |
| Population*Treatment*Spl | WY | Control |  | Dex |  | 0 | . | . | . |
| Population*Treatment*Spl | WY | Predator |  | Base |  | 0 | . | . | . |
| Population*Treatment*Spl | WY | Predator |  | Str |  | 0 | . | . | . |
| Population*Treatment*Spl | WY | Predator |  | Dex |  | 0 | . | . | . |
| Treatment*Age*Spl |  | Control | ASY | Base |  | -1.1978 | 0.5587 | -2.2946 | -0.1009 |
| Treatment*Age*Spl |  | Control | ASY | Str |  | -0.7411 | 0.5723 | -1.8646 | 0.3824 |
| Treatment*Age*Spl |  | Control | ASY | Dex |  | 0.2697 | 0.5648 | -0.8391 | 1.3786 |
| Treatment*Age*Spl |  | Control | SY | Base |  | 0 | . | . | . |
| Treatment*Age*Spl |  | Control | SY | Str |  | 0 | . | . | . |
| Treatment*Age*Spl |  | Control | SY | Dex |  | 0 | . | . | . |
| Treatment*Age*Spl |  | Low_Tape | ASY | Base |  | 0.07194 | 0.5112 | -0.9315 | 1.0753 |
| Treatment*Age*Spl |  | Low_Tape | ASY | Str |  | -0.1806 | 0.5112 | -1.184 | 0.8228 |
| Treatment*Age*Spl |  | Low_Tape | ASY | Dex |  | -0.1099 | 0.5112 | -1.1133 | 0.8935 |
| Treatment*Age*Spl |  | Low_Tape | SY | Base |  | 0 | . | . | . |
| Treatment*Age*Spl |  | Low_Tape | SY | Str |  | 0 | . | . | . |
| Treatment*Age*Spl |  | Low_Tape | SY | Dex |  | 0 | . | . | . |
| Treatment*Age*Spl |  | Predator | ASY | Base |  | 0 | . | . | . |
| Treatment*Age*Spl |  | Predator | ASY | Str |  | 0 | . | . | . |
| Treatment*Age*Spl |  | Predator | ASY | Dex |  | 0 | . | . | . |
| Treatment*Age*Spl |  | Predator | SY | Base |  | 0 | . | . | . |
| Treatment*Age*Spl |  | Predator | SY | Str |  | 0 | . | . | . |
| Treatment*Age*Spl |  | Predator | SY | Dex |  | 0 | . | . | . |
| Treatment*LHSS*Spl |  | Control |  | Base | Mid Inc | -0.3893 | 0.4783 | -1.3277 | 0.5491 |
| Treatment*LHSS*Spl |  | Control |  | Str | Mid Inc | -0.1795 | 0.4783 | -1.1179 | 0.7589 |
| Treatment*LHSS*Spl |  | Control |  | Dex | Mid Inc | 0.3728 | 0.4783 | -0.5656 | 1.3111 |
| Treatment*LHSS*Spl |  | Control |  | Base | End Inc | -0.884 | 0.5189 | -1.9021 | 0.134 |
| Treatment*LHSS*Spl |  | Control |  | Base | Nestling | 0 | . | . | . |
| Treatment*LHSS*Spl |  | Control |  | Str | Nestling | 0 | . | . | . |
| Treatment*LHSS*Spl |  | Control |  | Dex | Nestling | 0 | . | . | . |
| Treatment*LHSS*Spl |  | Low_Tape |  | Base | Mid Inc | -0.7148 | 0.4523 | -1.6022 | 0.1725 |
| Treatment*LHSS*Spl |  | Low_Tape |  | Str | Mid Inc | -0.4249 | 0.4523 | -1.3122 | 0.4624 |
| Treatment*LHSS*Spl |  | Low_Tape |  | Dex | Mid Inc | 0.1471 | 0.4523 | -0.7402 | 1.0344 |
| Treatment*LHSS*Spl |  | Low_Tape |  | Base | End Inc | -0.5679 | 0.4815 | -1.5125 | 0.3768 |
| Treatment*LHSS*Spl |  | Low_Tape |  | Base | Nestling | 0 | . | . | . |
| Treatment*LHSS*Spl |  | Low_Tape |  | Str | Nestling | 0 | . | . | . |
| Treatment*LHSS*Spl |  | Low_Tape |  | Dex | Nestling | 0 | . | . | . |
| Treatment*LHSS*Spl |  | Predator |  | Base | Mid Inc | 0 | . | . | . |
| Treatment*LHSS*Spl |  | Predator |  | Str | Mid Inc | 0 | . | . | . |
| Treatment*LHSS*Spl |  | Predator |  | Dex | Mid Inc | 0 | . | . | . |
| Treatment*LHSS*Spl |  | Predator |  | Base | End Inc | 0 | . | . | . |
| Treatment*LHSS*Spl |  | Predator |  | Base | Nestling | 0 | . | . | . |
| Treatment*LHSS*Spl |  | Predator |  | Str | Nestling | 0 | . | . | . |
| Treatment*LHSS*Spl |  | Predator |  | Dex | Nestling | 0 | . | . | . |
| Population*LHSS*Spl | AK |  |  | Base | Mid Inc | -0.7034 | 0.4212 | -1.5298 | 0.123 |
| Population*LHSS*Spl | AK |  |  | Str | Mid Inc | -0.8344 | 0.4212 | -1.6608 | -0.00802 |
| Population*LHSS*Spl | AK |  |  | Dex | Mid Inc | 0.3969 | 0.4212 | -0.4295 | 1.2233 |
| Population*LHSS*Spl | AK |  |  | Base | End Inc | -0.4092 | 0.451 | -1.2941 | 0.4756 |
| Population*LHSS*Spl | AK |  |  | Base | Nestling | 0 | . | . | . |
| Population*LHSS*Spl | AK |  |  | Str | Nestling | 0 | . | . | . |
| Population*LHSS*Spl | AK |  |  | Dex | Nestling | 0 | . | . | . |
| Population*LHSS*Spl | NY |  |  | Base | Mid Inc | -1.7741 | 0.5002 | -2.7556 | -0.7927 |
| Population*LHSS*Spl | NY |  |  | Str | Mid Inc | -0.4099 | 0.5002 | -1.3913 | 0.5716 |
| Population*LHSS*Spl | NY |  |  | Dex | Mid Inc | 0.6971 | 0.5002 | -0.2843 | 1.6786 |
| Population*LHSS*Spl | NY |  |  | Base | End Inc | -1.5598 | 0.5311 | -2.6018 | -0.5177 |
| Population*LHSS*Spl | NY |  |  | Base | Nestling | 0 | . | . | . |
| Population*LHSS*Spl | NY |  |  | Str | Nestling | 0 | . | . | . |
| Population*LHSS*Spl | NY |  |  | Dex | Nestling | 0 | . | . | . |
| Population*LHSS*Spl | TN |  |  | Base | Mid Inc | 0.2604 | 0.453 | -0.6284 | 1.1492 |
| Population*LHSS*Spl | TN |  |  | Str | Mid Inc | -0.3252 | 0.453 | -1.214 | 0.5636 |
| Population*LHSS*Spl | TN |  |  | Dex | Mid Inc | -0.0197 | 0.453 | -0.9085 | 0.8691 |
| Population*LHSS*Spl | TN |  |  | Base | End Inc | 0.1426 | 0.4804 | -0.8 | 1.0852 |
| Population*LHSS*Spl | TN |  |  | Base | Nestling | 0 | . | . | . |
| Population*LHSS*Spl | TN |  |  | Str | Nestling | 0 | . | . | . |
| Population*LHSS*Spl | TN |  |  | Dex | Nestling | 0 | . | . | . |
| Population*LHSS*Spl | WY |  |  | Base | Mid Inc | 0 | . | . | . |
| Population*LHSS*Spl | WY |  |  | Str | Mid Inc | 0 | . | . | . |
| Population*LHSS*Spl | WY |  |  | Dex | Mid Inc | 0 | . | . | . |
| Population*LHSS*Spl | WY |  |  | Base | End Inc | 0 | . | . | . |
| Population*LHSS*Spl | WY |  |  | Base | Nestling | 0 | . | . | . |
| Population*LHSS*Spl | WY |  |  | Str | Nestling | 0 | . | . | . |
| Population*LHSS*Spl | WY |  |  | Dex | Nestling | 0 | . | . | . |
| Age*LHSS*Spl |  |  | ASY | Base | Mid Inc | -0.7017 | 0.4173 | -1.5204 | 0.1169 |
| Age*LHSS*Spl |  |  | ASY | Str | Mid Inc | -0.2809 | 0.4353 | -1.135 | 0.5731 |
| Age*LHSS*Spl |  |  | ASY | Dex | Mid Inc | 0.2368 | 0.426 | -0.5991 | 1.0727 |
| Age*LHSS*Spl |  |  | ASY | Base | End Inc | -0.6868 | 0.4611 | -1.5916 | 0.2179 |
| Age*LHSS*Spl |  |  | ASY | Base | Nestling | 0 | . | . | . |
| Age*LHSS*Spl |  |  | ASY | Str | Nestling | 0 | . | . | . |
| Age*LHSS*Spl |  |  | ASY | Dex | Nestling | 0 | . | . | . |
| Age*LHSS*Spl |  |  | SY | Base | Mid Inc | 0 | . | . | . |
| Age*LHSS*Spl |  |  | SY | Str | Mid Inc | 0 | . | . | . |
| Age*LHSS*Spl |  |  | SY | Dex | Mid Inc | 0 | . | . | . |
| Age*LHSS*Spl |  |  | SY | Base | End Inc | 0 | . | . | . |
| Age*LHSS*Spl |  |  | SY | Base | Nestling | 0 | . | . | . |
| Age*LHSS*Spl |  |  | SY | Str | Nestling | 0 | . | . | . |
| Age*LHSS*Spl |  |  | SY | Dex | Nestling | 0 | . | . | . |
| Population*Age*LHSS*Spl | AK |  | ASY | Base | Mid Inc | 0.8727 | 0.5745 | -0.2545 | 1.9998 |
| Population*Age*LHSS*Spl | AK |  | ASY | Str | Mid Inc | 0.371 | 0.5877 | -0.7821 | 1.524 |
| Population*Age*LHSS*Spl | AK |  | ASY | Dex | Mid Inc | -0.497 | 0.5809 | -1.6367 | 0.6427 |
| Population*Age*LHSS*Spl | AK |  | ASY | Base | End Inc | 0.4574 | 0.61 | -0.7394 | 1.6542 |
| Population*Age*LHSS*Spl | AK |  | ASY | Base | Nestling | 0 | . | . | . |
| Population*Age*LHSS*Spl | AK |  | ASY | Str | Nestling | 0 | . | . | . |
| Population*Age*LHSS*Spl | AK |  | ASY | Dex | Nestling | 0 | . | . | . |
| Population*Age*LHSS*Spl | AK |  | SY | Base | Mid Inc | 0 | . | . | . |
| Population*Age*LHSS*Spl | AK |  | SY | Str | Mid Inc | 0 | . | . | . |
| Population*Age*LHSS*Spl | AK |  | SY | Dex | Mid Inc | 0 | . | . | . |
| Population*Age*LHSS*Spl | AK |  | SY | Base | End Inc | 0 | . | . | . |
| Population*Age*LHSS*Spl | AK |  | SY | Base | Nestling | 0 | . | . | . |
| Population*Age*LHSS*Spl | AK |  | SY | Str | Nestling | 0 | . | . | . |
| Population*Age*LHSS*Spl | AK |  | SY | Dex | Nestling | 0 | . | . | . |
| Population*Age*LHSS*Spl | NY |  | ASY | Base | Mid Inc | 0.7334 | 0.7055 | -0.6508 | 2.1175 |
| Population*Age*LHSS*Spl | NY |  | ASY | Str | Mid Inc | -0.01601 | 0.7162 | -1.4213 | 1.3893 |
| Population*Age*LHSS*Spl | NY |  | ASY | Dex | Mid Inc | -0.5542 | 0.7106 | -1.9485 | 0.8401 |
| Population*Age*LHSS*Spl | NY |  | ASY | Base | End Inc | 2.1341 | 0.7858 | 0.5922 | 3.6759 |
| Population*Age*LHSS*Spl | NY |  | ASY | Base | Nestling | 0 | . | . | . |
| Population*Age*LHSS*Spl | NY |  | ASY | Str | Nestling | 0 | . | . | . |
| Population*Age*LHSS*Spl | NY |  | ASY | Dex | Nestling | 0 | . | . | . |
| Population*Age*LHSS*Spl | NY |  | SY | Base | Mid Inc | 0 | . | . | . |
| Population*Age*LHSS*Spl | NY |  | SY | Str | Mid Inc | 0 | . | . | . |
| Population*Age*LHSS*Spl | NY |  | SY | Dex | Mid Inc | 0 | . | . | . |
| Population*Age*LHSS*Spl | NY |  | SY | Base | End Inc | 0 | . | . | . |
| Population*Age*LHSS*Spl | NY |  | SY | Base | Nestling | 0 | . | . | . |
| Population*Age*LHSS*Spl | NY |  | SY | Str | Nestling | 0 | . | . | . |
| Population*Age*LHSS*Spl | NY |  | SY | Dex | Nestling | 0 | . | . | . |
| Population*Age*LHSS*Spl | TN |  | ASY | Base | Mid Inc | -0.05746 | 0.5943 | -1.2235 | 1.1086 |
| Population*Age*LHSS*Spl | TN |  | ASY | Str | Mid Inc | 0.1431 | 0.6071 | -1.0481 | 1.3342 |
| Population*Age*LHSS*Spl | TN |  | ASY | Dex | Mid Inc | 0.1122 | 0.6005 | -1.066 | 1.2904 |
| Population*Age*LHSS*Spl | TN |  | ASY | Base | End Inc | 0.2852 | 0.6299 | -0.9506 | 1.521 |
| Population*Age*LHSS*Spl | TN |  | ASY | Base | Nestling | 0 | . | . | . |
| Population*Age*LHSS*Spl | TN |  | ASY | Str | Nestling | 0 | . | . | . |
| Population*Age*LHSS*Spl | TN |  | ASY | Dex | Nestling | 0 | . | . | . |
| Population*Age*LHSS*Spl | TN |  | SY | Base | Mid Inc | 0 | . | . | . |
| Population*Age*LHSS*Spl | TN |  | SY | Str | Mid Inc | 0 | . | . | . |
| Population*Age*LHSS*Spl | TN |  | SY | Dex | Mid Inc | 0 | . | . | . |
| Population*Age*LHSS*Spl | TN |  | SY | Base | End Inc | 0 | . | . | . |
| Population*Age*LHSS*Spl | TN |  | SY | Base | Nestling | 0 | . | . | . |
| Population*Age*LHSS*Spl | TN |  | SY | Str | Nestling | 0 | . | . | . |
| Population*Age*LHSS*Spl | TN |  | SY | Dex | Nestling | 0 | . | . | . |
| Population*Age*LHSS*Spl | WY |  | ASY | Base | Mid Inc | 0 | . | . | . |
| Population*Age*LHSS*Spl | WY |  | ASY | Str | Mid Inc | 0 | . | . | . |
| Population*Age*LHSS*Spl | WY |  | ASY | Dex | Mid Inc | 0 | . | . | . |
| Population*Age*LHSS*Spl | WY |  | ASY | Base | End Inc | 0 | . | . | . |
| Population*Age*LHSS*Spl | WY |  | ASY | Base | Nestling | 0 | . | . | . |
| Population*Age*LHSS*Spl | WY |  | ASY | Str | Nestling | 0 | . | . | . |
| Population*Age*LHSS*Spl | WY |  | ASY | Dex | Nestling | 0 | . | . | . |
| Population*Age*LHSS*Spl | WY |  | SY | Base | Mid Inc | 0 | . | . | . |
| Population*Age*LHSS*Spl | WY |  | SY | Str | Mid Inc | 0 | . | . | . |
| Population*Age*LHSS*Spl | WY |  | SY | Dex | Mid Inc | 0 | . | . | . |
| Population*Age*LHSS*Spl | WY |  | SY | Base | End Inc | 0 | . | . | . |
| Population*Age*LHSS*Spl | WY |  | SY | Base | Nestling | 0 | . | . | . |
| Population*Age*LHSS*Spl | WY |  | SY | Str | Nestling | 0 | . | . | . |
| Population*Age*LHSS*Spl | WY |  | SY | Dex | Nestling | 0 | . | . | . |
| Population*Treatment*Age*Spl | AK | Control | ASY | Base |  | 2.2478 | 0.718 | 0.8389 | 3.6566 |
| Population*Treatment*Age*Spl | AK | Control | ASY | Str |  | 0.729 | 0.7287 | -0.7008 | 2.1588 |
| Population*Treatment*Age*Spl | AK | Control | ASY | Dex |  | 0.1268 | 0.7228 | -1.2915 | 1.5451 |
| Population*Treatment*Age*Spl | AK | Control | SY | Base |  | 0 | . | . | . |
| Population*Treatment*Age*Spl | AK | Control | SY | Str |  | 0 | . | . | . |
| Population*Treatment*Age*Spl | AK | Control | SY | Dex |  | 0 | . | . | . |
| Population*Treatment*Age*Spl | AK | Low_Tape | ASY | Base |  | 0.9083 | 0.7239 | -0.512 | 2.3286 |
| Population*Treatment*Age*Spl | AK | Low_Tape | ASY | Str |  | 0.3774 | 0.7239 | -1.0429 | 1.7977 |
| Population*Treatment*Age*Spl | AK | Low_Tape | ASY | Dex |  | 0.8124 | 0.7283 | -0.6165 | 2.2414 |
| Population*Treatment*Age*Spl | AK | Low_Tape | SY | Base |  | 0 | . | . | . |
| Population*Treatment*Age*Spl | AK | Low_Tape | SY | Str |  | 0 | . | . | . |
| Population*Treatment*Age*Spl | AK | Low_Tape | SY | Dex |  | 0 | . | . | . |
| Population*Treatment*Age*Spl | AK | Predator | ASY | Base |  | 0 | . | . | . |
| Population*Treatment*Age*Spl | AK | Predator | ASY | Str |  | 0 | . | . | . |
| Population*Treatment*Age*Spl | AK | Predator | ASY | Dex |  | 0 | . | . | . |
| Population*Treatment*Age*Spl | AK | Predator | SY | Base |  | 0 | . | . | . |
| Population*Treatment*Age*Spl | AK | Predator | SY | Str |  | 0 | . | . | . |
| Population*Treatment*Age*Spl | AK | Predator | SY | Dex |  | 0 | . | . | . |
| Population*Treatment*Age*Spl | NY | Control | ASY | Base |  | 1.3907 | 0.8363 | -0.2503 | 3.0316 |
| Population*Treatment*Age*Spl | NY | Control | ASY | Str |  | 0.4213 | 0.8581 | -1.2624 | 2.1049 |
| Population*Treatment*Age*Spl | NY | Control | ASY | Dex |  | -0.7576 | 0.8531 | -2.4315 | 0.9162 |
| Population*Treatment*Age*Spl | NY | Control | SY | Base |  | 0 | . | . | . |
| Population*Treatment*Age*Spl | NY | Control | SY | Str |  | 0 | . | . | . |
| Population*Treatment*Age*Spl | NY | Control | SY | Dex |  | 0 | . | . | . |
| Population*Treatment*Age*Spl | NY | Low_Tape | ASY | Base |  | 0.6862 | 0.9751 | -1.227 | 2.5993 |
| Population*Treatment*Age*Spl | NY | Low_Tape | ASY | Str |  | -0.05479 | 0.9751 | -1.9679 | 1.8583 |
| Population*Treatment*Age*Spl | NY | Low_Tape | ASY | Dex |  | 0.9804 | 0.9751 | -0.9327 | 2.8935 |
| Population*Treatment*Age*Spl | NY | Low_Tape | SY | Base |  | 0 | . | . | . |
| Population*Treatment*Age*Spl | NY | Low_Tape | SY | Str |  | 0 | . | . | . |
| Population*Treatment*Age*Spl | NY | Low_Tape | SY | Dex |  | 0 | . | . | . |
| Population*Treatment*Age*Spl | NY | Predator | ASY | Base |  | 0 | . | . | . |
| Population*Treatment*Age*Spl | NY | Predator | ASY | Str |  | 0 | . | . | . |
| Population*Treatment*Age*Spl | NY | Predator | ASY | Dex |  | 0 | . | . | . |
| Population*Treatment*Age*Spl | NY | Predator | SY | Base |  | 0 | . | . | . |
| Population*Treatment*Age*Spl | NY | Predator | SY | Str |  | 0 | . | . | . |
| Population*Treatment*Age*Spl | NY | Predator | SY | Dex |  | 0 | . | . | . |
| Population*Treatment*Age*Spl | TN | Control | ASY | Base |  | 1.0845 | 0.7258 | -0.3397 | 2.5087 |
| Population*Treatment*Age*Spl | TN | Control | ASY | Str |  | 1.0298 | 0.7399 | -0.4221 | 2.4816 |
| Population*Treatment*Age*Spl | TN | Control | ASY | Dex |  | -0.3229 | 0.7305 | -1.7564 | 1.1106 |
| Population*Treatment*Age*Spl | TN | Control | SY | Base |  | 0 | . | . | . |
| Population*Treatment*Age*Spl | TN | Control | SY | Str |  | 0 | . | . | . |
| Population*Treatment*Age*Spl | TN | Control | SY | Dex |  | 0 | . | . | . |
| Population*Treatment*Age*Spl | TN | Low_Tape | ASY | Base |  | 0 | . | . | . |
| Population*Treatment*Age*Spl | TN | Low_Tape | ASY | Str |  | 0 | . | . | . |
| Population*Treatment*Age*Spl | TN | Low_Tape | ASY | Dex |  | 0 | . | . | . |
| Population*Treatment*Age*Spl | TN | Low_Tape | SY | Base |  | 0 | . | . | . |
| Population*Treatment*Age*Spl | TN | Low_Tape | SY | Str |  | 0 | . | . | . |
| Population*Treatment*Age*Spl | TN | Low_Tape | SY | Dex |  | 0 | . | . | . |
| Population*Treatment*Age*Spl | TN | Predator | ASY | Base |  | 0 | . | . | . |
| Population*Treatment*Age*Spl | TN | Predator | ASY | Str |  | 0 | . | . | . |
| Population*Treatment*Age*Spl | TN | Predator | ASY | Dex |  | 0 | . | . | . |
| Population*Treatment*Age*Spl | TN | Predator | SY | Base |  | 0 | . | . | . |
| Population*Treatment*Age*Spl | TN | Predator | SY | Str |  | 0 | . | . | . |
| Population*Treatment*Age*Spl | TN | Predator | SY | Dex |  | 0 | . | . | . |
| Population*Treatment*Age*Spl | WY | Control | ASY | Base |  | 0 | . | . | . |
| Population*Treatment*Age*Spl | WY | Control | ASY | Str |  | 0 | . | . | . |
| Population*Treatment*Age*Spl | WY | Control | ASY | Dex |  | 0 | . | . | . |
| Population*Treatment*Age*Spl | WY | Control | SY | Base |  | 0 | . | . | . |
| Population*Treatment*Age*Spl | WY | Control | SY | Str |  | 0 | . | . | . |
| Population*Treatment*Age*Spl | WY | Control | SY | Dex |  | 0 | . | . | . |
| Population*Treatment*Age*Spl | WY | Predator | ASY | Base |  | 0 | . | . | . |
| Population*Treatment*Age*Spl | WY | Predator | ASY | Str |  | 0 | . | . | . |
| Population*Treatment*Age*Spl | WY | Predator | ASY | Dex |  | 0 | . | . | . |
| Population*Treatment*Age*Spl | WY | Predator | SY | Base |  | 0 | . | . | . |
| Population*Treatment*Age*Spl | WY | Predator | SY | Str |  | 0 | . | . | . |
| Population*Treatment*Age*Spl | WY | Predator | SY | Dex |  | 0 | . | . | . |
| Treatment*Age*LHSS*Spl |  | Control | ASY | Base | Mid Inc | 1.5352 | 0.5964 | 0.3651 | 2.7052 |
| Treatment*Age*LHSS*Spl |  | Control | ASY | Str | Mid Inc | 0.7974 | 0.6091 | -0.3977 | 1.9926 |
| Treatment*Age*LHSS*Spl |  | Control | ASY | Dex | Mid Inc | -0.2493 | 0.6032 | -1.4328 | 0.9343 |
| Treatment*Age*LHSS*Spl |  | Control | ASY | Base | End Inc | 1.207 | 0.6457 | -0.05993 | 2.4739 |
| Treatment*Age*LHSS*Spl |  | Control | ASY | Base | Nestling | 0 | . | . | . |
| Treatment*Age*LHSS*Spl |  | Control | ASY | Str | Nestling | 0 | . | . | . |
| Treatment*Age*LHSS*Spl |  | Control | ASY | Dex | Nestling | 0 | . | . | . |
| Treatment*Age*LHSS*Spl |  | Control | SY | Base | Mid Inc | 0 | . | . | . |
| Treatment*Age*LHSS*Spl |  | Control | SY | Str | Mid Inc | 0 | . | . | . |
| Treatment*Age*LHSS*Spl |  | Control | SY | Dex | Mid Inc | 0 | . | . | . |
| Treatment*Age*LHSS*Spl |  | Control | SY | Base | End Inc | 0 | . | . | . |
| Treatment*Age*LHSS*Spl |  | Control | SY | Base | Nestling | 0 | . | . | . |
| Treatment*Age*LHSS*Spl |  | Control | SY | Str | Nestling | 0 | . | . | . |
| Treatment*Age*LHSS*Spl |  | Control | SY | Dex | Nestling | 0 | . | . | . |
| Treatment*Age*LHSS*Spl |  | Low_Tape | ASY | Base | Mid Inc | 0.953 | 0.6143 | -0.2522 | 2.1583 |
| Treatment*Age*LHSS*Spl |  | Low_Tape | ASY | Str | Mid Inc | 1.0457 | 0.6143 | -0.1595 | 2.251 |
| Treatment*Age*LHSS*Spl |  | Low_Tape | ASY | Dex | Mid Inc | -0.2181 | 0.6143 | -1.4233 | 0.9872 |
| Treatment*Age*LHSS*Spl |  | Low_Tape | ASY | Base | End Inc | 0.355 | 0.6416 | -0.9039 | 1.6139 |
| Treatment*Age*LHSS*Spl |  | Low_Tape | ASY | Base | Nestling | 0 | . | . | . |
| Treatment*Age*LHSS*Spl |  | Low_Tape | ASY | Str | Nestling | 0 | . | . | . |
| Treatment*Age*LHSS*Spl |  | Low_Tape | ASY | Dex | Nestling | 0 | . | . | . |
| Treatment*Age*LHSS*Spl |  | Low_Tape | SY | Base | Mid Inc | 0 | . | . | . |
| Treatment*Age*LHSS*Spl |  | Low_Tape | SY | Str | Mid Inc | 0 | . | . | . |
| Treatment*Age*LHSS*Spl |  | Low_Tape | SY | Dex | Mid Inc | 0 | . | . | . |
| Treatment*Age*LHSS*Spl |  | Low_Tape | SY | Base | End Inc | 0 | . | . | . |
| Treatment*Age*LHSS*Spl |  | Low_Tape | SY | Base | Nestling | 0 | . | . | . |
| Treatment*Age*LHSS*Spl |  | Low_Tape | SY | Str | Nestling | 0 | . | . | . |
| Treatment*Age*LHSS*Spl |  | Low_Tape | SY | Dex | Nestling | 0 | . | . | . |
| Treatment*Age*LHSS*Spl |  | Predator | ASY | Base | Mid Inc | 0 | . | . | . |
| Treatment*Age*LHSS*Spl |  | Predator | ASY | Str | Mid Inc | 0 | . | . | . |
| Treatment*Age*LHSS*Spl |  | Predator | ASY | Dex | Mid Inc | 0 | . | . | . |
| Treatment*Age*LHSS*Spl |  | Predator | ASY | Base | End Inc | 0 | . | . | . |
| Treatment*Age*LHSS*Spl |  | Predator | ASY | Base | Nestling | 0 | . | . | . |
| Treatment*Age*LHSS*Spl |  | Predator | ASY | Str | Nestling | 0 | . | . | . |
| Treatment*Age*LHSS*Spl |  | Predator | ASY | Dex | Nestling | 0 | . | . | . |
| Treatment*Age*LHSS*Spl |  | Predator | SY | Base | Mid Inc | 0 | . | . | . |
| Treatment*Age*LHSS*Spl |  | Predator | SY | Str | Mid Inc | 0 | . | . | . |
| Treatment*Age*LHSS*Spl |  | Predator | SY | Dex | Mid Inc | 0 | . | . | . |
| Treatment*Age*LHSS*Spl |  | Predator | SY | Base | End Inc | 0 | . | . | . |
| Treatment*Age*LHSS*Spl |  | Predator | SY | Base | Nestling | 0 | . | . | . |
| Treatment*Age*LHSS*Spl |  | Predator | SY | Str | Nestling | 0 | . | . | . |
| Treatment*Age*LHSS*Spl |  | Predator | SY | Dex | Nestling | 0 | . | . | . |
| Population*Treatment*Age*LHSS*Spl | AK | Control | ASY | Base | Mid Inc | -0.8793 | 0.5465 | -1.9516 | 0.1929 |
| Population*Treatment*Age*LHSS*Spl | AK | Control | ASY | Str | Mid Inc | -0.6148 | 0.5604 | -1.7143 | 0.4847 |
| Population*Treatment*Age*LHSS*Spl | AK | Control | ASY | Dex | Mid Inc | -0.1055 | 0.5574 | -1.1992 | 0.9881 |
| Population*Treatment*Age*LHSS*Spl | AK | Control | ASY | Base | End Inc | -0.5412 | 0.5757 | -1.6709 | 0.5884 |
| Population*Treatment*Age*LHSS*Spl | AK | Control | ASY | Base | Nestling | 0 | . | . | . |
| Population*Treatment*Age*LHSS*Spl | AK | Control | ASY | Str | Nestling | 0 | . | . | . |
| Population*Treatment*Age*LHSS*Spl | AK | Control | ASY | Dex | Nestling | 0 | . | . | . |
| Population*Treatment*Age*LHSS*Spl | AK | Control | SY | Base | Mid Inc | 1.0336 | 0.625 | -0.1926 | 2.2599 |
| Population*Treatment*Age*LHSS*Spl | AK | Control | SY | Str | Mid Inc | -0.1788 | 0.6286 | -1.412 | 1.0545 |
| Population*Treatment*Age*LHSS*Spl | AK | Control | SY | Dex | Mid Inc | -0.07632 | 0.625 | -1.3026 | 1.1499 |
| Population*Treatment*Age*LHSS*Spl | AK | Control | SY | Base | End Inc | 1.3183 | 0.6704 | 0.003073 | 2.6336 |
| Population*Treatment*Age*LHSS*Spl | AK | Control | SY | Base | Nestling | 0 | . | . | . |
| Population*Treatment*Age*LHSS*Spl | AK | Control | SY | Str | Nestling | 0 | . | . | . |
| Population*Treatment*Age*LHSS*Spl | AK | Control | SY | Dex | Nestling | 0 | . | . | . |
| Population*Treatment*Age*LHSS*Spl | AK | Low_Tape | ASY | Base | Mid Inc | -0.4049 | 0.5814 | -1.5455 | 0.7358 |
| Population*Treatment*Age*LHSS*Spl | AK | Low_Tape | ASY | Str | Mid Inc | -0.1296 | 0.5814 | -1.2702 | 1.0111 |
| Population*Treatment*Age*LHSS*Spl | AK | Low_Tape | ASY | Dex | Mid Inc | 0.6314 | 0.5869 | -0.52 | 1.7829 |
| Population*Treatment*Age*LHSS*Spl | AK | Low_Tape | ASY | Base | End Inc | 0.2413 | 0.5936 | -0.9234 | 1.406 |
| Population*Treatment*Age*LHSS*Spl | AK | Low_Tape | ASY | Base | Nestling | 0 | . | . | . |
| Population*Treatment*Age*LHSS*Spl | AK | Low_Tape | ASY | Str | Nestling | 0 | . | . | . |
| Population*Treatment*Age*LHSS*Spl | AK | Low_Tape | ASY | Dex | Nestling | 0 | . | . | . |
| Population*Treatment*Age*LHSS*Spl | AK | Low_Tape | SY | Base | Mid Inc | 1.3518 | 0.6562 | 0.06441 | 2.6392 |
| Population*Treatment*Age*LHSS*Spl | AK | Low_Tape | SY | Str | Mid Inc | 1.0963 | 0.6562 | -0.1911 | 2.3837 |
| Population*Treatment*Age*LHSS*Spl | AK | Low_Tape | SY | Dex | Mid Inc | 0.4181 | 0.6562 | -0.8693 | 1.7055 |
| Population*Treatment*Age*LHSS*Spl | AK | Low_Tape | SY | Base | End Inc | 0.7942 | 0.7092 | -0.5973 | 2.1858 |
| Population*Treatment*Age*LHSS*Spl | AK | Low_Tape | SY | Base | Nestling | 0 | . | . | . |
| Population*Treatment*Age*LHSS*Spl | AK | Low_Tape | SY | Str | Nestling | 0 | . | . | . |
| Population*Treatment*Age*LHSS*Spl | AK | Low_Tape | SY | Dex | Nestling | 0 | . | . | . |
| Population*Treatment*Age*LHSS*Spl | AK | Predator | ASY | Base | Mid Inc | 0 | . | . | . |
| Population*Treatment*Age*LHSS*Spl | AK | Predator | ASY | Str | Mid Inc | 0 | . | . | . |
| Population*Treatment*Age*LHSS*Spl | AK | Predator | ASY | Dex | Mid Inc | 0 | . | . | . |
| Population*Treatment*Age*LHSS*Spl | AK | Predator | ASY | Base | End Inc | 0 | . | . | . |
| Population*Treatment*Age*LHSS*Spl | AK | Predator | ASY | Base | Nestling | 0 | . | . | . |
| Population*Treatment*Age*LHSS*Spl | AK | Predator | ASY | Str | Nestling | 0 | . | . | . |
| Population*Treatment*Age*LHSS*Spl | AK | Predator | ASY | Dex | Nestling | 0 | . | . | . |
| Population*Treatment*Age*LHSS*Spl | AK | Predator | SY | Base | Mid Inc | 0 | . | . | . |
| Population*Treatment*Age*LHSS*Spl | AK | Predator | SY | Str | Mid Inc | 0 | . | . | . |
| Population*Treatment*Age*LHSS*Spl | AK | Predator | SY | Dex | Mid Inc | 0 | . | . | . |
| Population*Treatment*Age*LHSS*Spl | AK | Predator | SY | Base | End Inc | 0 | . | . | . |
| Population*Treatment*Age*LHSS*Spl | AK | Predator | SY | Base | Nestling | 0 | . | . | . |
| Population*Treatment*Age*LHSS*Spl | AK | Predator | SY | Str | Nestling | 0 | . | . | . |
| Population*Treatment*Age*LHSS*Spl | AK | Predator | SY | Dex | Nestling | 0 | . | . | . |
| Population*Treatment*Age*LHSS*Spl | NY | Control | ASY | Base | Mid Inc | 0.1145 | 0.6718 | -1.2036 | 1.4325 |
| Population*Treatment*Age*LHSS*Spl | NY | Control | ASY | Str | Mid Inc | -0.3799 | 0.6986 | -1.7506 | 0.9908 |
| Population*Treatment*Age*LHSS*Spl | NY | Control | ASY | Dex | Mid Inc | 0.1422 | 0.6933 | -1.2182 | 1.5025 |
| Population*Treatment*Age*LHSS*Spl | NY | Control | ASY | Base | End Inc | -0.5528 | 0.7529 | -2.0301 | 0.9244 |
| Population*Treatment*Age*LHSS*Spl | NY | Control | ASY | Base | Nestling | 0 | . | . | . |
| Population*Treatment*Age*LHSS*Spl | NY | Control | ASY | Str | Nestling | 0 | . | . | . |
| Population*Treatment*Age*LHSS*Spl | NY | Control | ASY | Dex | Nestling | 0 | . | . | . |
| Population*Treatment*Age*LHSS*Spl | NY | Control | SY | Base | Mid Inc | 1.1437 | 0.7277 | -0.2841 | 2.5714 |
| Population*Treatment*Age*LHSS*Spl | NY | Control | SY | Str | Mid Inc | 0.1167 | 0.7277 | -1.311 | 1.5445 |
| Population*Treatment*Age*LHSS*Spl | NY | Control | SY | Dex | Mid Inc | -0.5272 | 0.7317 | -1.9628 | 0.9084 |
| Population*Treatment*Age*LHSS*Spl | NY | Control | SY | Base | End Inc | 1.7607 | 0.7699 | 0.2501 | 3.2714 |
| Population*Treatment*Age*LHSS*Spl | NY | Control | SY | Base | Nestling | 0 | . | . | . |
| Population*Treatment*Age*LHSS*Spl | NY | Control | SY | Str | Nestling | 0 | . | . | . |
| Population*Treatment*Age*LHSS*Spl | NY | Control | SY | Dex | Nestling | 0 | . | . | . |
| Population*Treatment*Age*LHSS*Spl | NY | Low_Tape | ASY | Base | Mid Inc | 0.5229 | 0.6869 | -0.8248 | 1.8706 |
| Population*Treatment*Age*LHSS*Spl | NY | Low_Tape | ASY | Str | Mid Inc | -0.3543 | 0.6869 | -1.702 | 0.9934 |
| Population*Treatment*Age*LHSS*Spl | NY | Low_Tape | ASY | Dex | Mid Inc | -0.3653 | 0.6869 | -1.713 | 0.9824 |
| Population*Treatment*Age*LHSS*Spl | NY | Low_Tape | ASY | Base | End Inc | 0.005967 | 0.7491 | -1.4637 | 1.4757 |
| Population*Treatment*Age*LHSS*Spl | NY | Low_Tape | ASY | Base | Nestling | 0 | . | . | . |
| Population*Treatment*Age*LHSS*Spl | NY | Low_Tape | ASY | Str | Nestling | 0 | . | . | . |
| Population*Treatment*Age*LHSS*Spl | NY | Low_Tape | ASY | Dex | Nestling | 0 | . | . | . |
| Population*Treatment*Age*LHSS*Spl | NY | Low_Tape | SY | Base | Mid Inc | 2.3602 | 0.9395 | 0.5168 | 4.2036 |
| Population*Treatment*Age*LHSS*Spl | NY | Low_Tape | SY | Str | Mid Inc | 0.1866 | 0.9395 | -1.6568 | 2.03 |
| Population*Treatment*Age*LHSS*Spl | NY | Low_Tape | SY | Dex | Mid Inc | -0.01124 | 0.9395 | -1.8547 | 1.8322 |
| Population*Treatment*Age*LHSS*Spl | NY | Low_Tape | SY | Base | End Inc | 1.4244 | 1.1131 | -0.7597 | 3.6085 |
| Population*Treatment*Age*LHSS*Spl | NY | Low_Tape | SY | Base | Nestling | 0 | . | . | . |
| Population*Treatment*Age*LHSS*Spl | NY | Low_Tape | SY | Str | Nestling | 0 | . | . | . |
| Population*Treatment*Age*LHSS*Spl | NY | Low_Tape | SY | Dex | Nestling | 0 | . | . | . |
| Population*Treatment*Age*LHSS*Spl | NY | Predator | ASY | Base | Mid Inc | 0 | . | . | . |
| Population*Treatment*Age*LHSS*Spl | NY | Predator | ASY | Str | Mid Inc | 0 | . | . | . |
| Population*Treatment*Age*LHSS*Spl | NY | Predator | ASY | Dex | Mid Inc | 0 | . | . | . |
| Population*Treatment*Age*LHSS*Spl | NY | Predator | ASY | Base | End Inc | 0 | . | . | . |
| Population*Treatment*Age*LHSS*Spl | NY | Predator | ASY | Base | Nestling | 0 | . | . | . |
| Population*Treatment*Age*LHSS*Spl | NY | Predator | ASY | Str | Nestling | 0 | . | . | . |
| Population*Treatment*Age*LHSS*Spl | NY | Predator | ASY | Dex | Nestling | 0 | . | . | . |
| Population*Treatment*Age*LHSS*Spl | NY | Predator | SY | Base | Mid Inc | 0 | . | . | . |
| Population*Treatment*Age*LHSS*Spl | NY | Predator | SY | Str | Mid Inc | 0 | . | . | . |
| Population*Treatment*Age*LHSS*Spl | NY | Predator | SY | Dex | Mid Inc | 0 | . | . | . |
| Population*Treatment*Age*LHSS*Spl | NY | Predator | SY | Base | End Inc | 0 | . | . | . |
| Population*Treatment*Age*LHSS*Spl | NY | Predator | SY | Base | Nestling | 0 | . | . | . |
| Population*Treatment*Age*LHSS*Spl | NY | Predator | SY | Str | Nestling | 0 | . | . | . |
| Population*Treatment*Age*LHSS*Spl | NY | Predator | SY | Dex | Nestling | 0 | . | . | . |
| Population*Treatment*Age*LHSS*Spl | TN | Control | ASY | Base | Mid Inc | -1.2519 | 0.5362 | -2.3039 | -0.1998 |
| Population*Treatment*Age*LHSS*Spl | TN | Control | ASY | Str | Mid Inc | -0.1214 | 0.555 | -1.2103 | 0.9676 |
| Population*Treatment*Age*LHSS*Spl | TN | Control | ASY | Dex | Mid Inc | -0.03865 | 0.5437 | -1.1055 | 1.0282 |
| Population*Treatment*Age*LHSS*Spl | TN | Control | ASY | Base | End Inc | -0.06358 | 0.5584 | -1.1592 | 1.032 |
| Population*Treatment*Age*LHSS*Spl | TN | Control | ASY | Base | Nestling | 0 | . | . | . |
| Population*Treatment*Age*LHSS*Spl | TN | Control | ASY | Str | Nestling | 0 | . | . | . |
| Population*Treatment*Age*LHSS*Spl | TN | Control | ASY | Dex | Nestling | 0 | . | . | . |
| Population*Treatment*Age*LHSS*Spl | TN | Control | SY | Base | Mid Inc | 0.08723 | 0.6375 | -1.1636 | 1.3381 |
| Population*Treatment*Age*LHSS*Spl | TN | Control | SY | Str | Mid Inc | 0.09063 | 0.6375 | -1.1602 | 1.3414 |
| Population*Treatment*Age*LHSS*Spl | TN | Control | SY | Dex | Mid Inc | -0.1436 | 0.6375 | -1.3944 | 1.1073 |
| Population*Treatment*Age*LHSS*Spl | TN | Control | SY | Base | End Inc | 0.2868 | 0.6722 | -1.0321 | 1.6056 |
| Population*Treatment*Age*LHSS*Spl | TN | Control | SY | Base | Nestling | 0 | . | . | . |
| Population*Treatment*Age*LHSS*Spl | TN | Control | SY | Str | Nestling | 0 | . | . | . |
| Population*Treatment*Age*LHSS*Spl | TN | Control | SY | Dex | Nestling | 0 | . | . | . |
| Population*Treatment*Age*LHSS*Spl | TN | Low_Tape | ASY | Base | Mid Inc | 0 | . | . | . |
| Population*Treatment*Age*LHSS*Spl | TN | Low_Tape | ASY | Str | Mid Inc | 0 | . | . | . |
| Population*Treatment*Age*LHSS*Spl | TN | Low_Tape | ASY | Dex | Mid Inc | 0 | . | . | . |
| Population*Treatment*Age*LHSS*Spl | TN | Low_Tape | ASY | Base | End Inc | 0 | . | . | . |
| Population*Treatment*Age*LHSS*Spl | TN | Low_Tape | ASY | Base | Nestling | 0 | . | . | . |
| Population*Treatment*Age*LHSS*Spl | TN | Low_Tape | ASY | Str | Nestling | 0 | . | . | . |
| Population*Treatment*Age*LHSS*Spl | TN | Low_Tape | ASY | Dex | Nestling | 0 | . | . | . |
| Population*Treatment*Age*LHSS*Spl | TN | Low_Tape | SY | Base | Mid Inc | 0 | . | . | . |
| Population*Treatment*Age*LHSS*Spl | TN | Low_Tape | SY | Str | Mid Inc | 0 | . | . | . |
| Population*Treatment*Age*LHSS*Spl | TN | Low_Tape | SY | Dex | Mid Inc | 0 | . | . | . |
| Population*Treatment*Age*LHSS*Spl | TN | Low_Tape | SY | Base | End Inc | 0 | . | . | . |
| Population*Treatment*Age*LHSS*Spl | TN | Low_Tape | SY | Base | Nestling | 0 | . | . | . |
| Population*Treatment*Age*LHSS*Spl | TN | Low_Tape | SY | Str | Nestling | 0 | . | . | . |
| Population*Treatment*Age*LHSS*Spl | TN | Low_Tape | SY | Dex | Nestling | 0 | . | . | . |
| Population*Treatment*Age*LHSS*Spl | TN | Predator | ASY | Base | Mid Inc | 0 | . | . | . |
| Population*Treatment*Age*LHSS*Spl | TN | Predator | ASY | Str | Mid Inc | 0 | . | . | . |
| Population*Treatment*Age*LHSS*Spl | TN | Predator | ASY | Dex | Mid Inc | 0 | . | . | . |
| Population*Treatment*Age*LHSS*Spl | TN | Predator | ASY | Base | End Inc | 0 | . | . | . |
| Population*Treatment*Age*LHSS*Spl | TN | Predator | ASY | Base | Nestling | 0 | . | . | . |
| Population*Treatment*Age*LHSS*Spl | TN | Predator | ASY | Str | Nestling | 0 | . | . | . |
| Population*Treatment*Age*LHSS*Spl | TN | Predator | ASY | Dex | Nestling | 0 | . | . | . |
| Population*Treatment*Age*LHSS*Spl | TN | Predator | SY | Base | Mid Inc | 0 | . | . | . |
| Population*Treatment*Age*LHSS*Spl | TN | Predator | SY | Str | Mid Inc | 0 | . | . | . |
| Population*Treatment*Age*LHSS*Spl | TN | Predator | SY | Dex | Mid Inc | 0 | . | . | . |
| Population*Treatment*Age*LHSS*Spl | TN | Predator | SY | Base | End Inc | 0 | . | . | . |
| Population*Treatment*Age*LHSS*Spl | TN | Predator | SY | Base | Nestling | 0 | . | . | . |
| Population*Treatment*Age*LHSS*Spl | TN | Predator | SY | Str | Nestling | 0 | . | . | . |
| Population*Treatment*Age*LHSS*Spl | TN | Predator | SY | Dex | Nestling | 0 | . | . | . |
| Population*Treatment*Age*LHSS*Spl | WY | Control | ASY | Base | Mid Inc | 0 | . | . | . |
| Population*Treatment*Age*LHSS*Spl | WY | Control | ASY | Str | Mid Inc | 0 | . | . | . |
| Population*Treatment*Age*LHSS*Spl | WY | Control | ASY | Dex | Mid Inc | 0 | . | . | . |
| Population*Treatment*Age*LHSS*Spl | WY | Control | ASY | Base | End Inc | 0 | . | . | . |
| Population*Treatment*Age*LHSS*Spl | WY | Control | ASY | Base | Nestling | 0 | . | . | . |
| Population*Treatment*Age*LHSS*Spl | WY | Control | ASY | Str | Nestling | 0 | . | . | . |
| Population*Treatment*Age*LHSS*Spl | WY | Control | ASY | Dex | Nestling | 0 | . | . | . |
| Population*Treatment*Age*LHSS*Spl | WY | Control | SY | Base | Mid Inc | 0 | . | . | . |
| Population*Treatment*Age*LHSS*Spl | WY | Control | SY | Str | Mid Inc | 0 | . | . | . |
| Population*Treatment*Age*LHSS*Spl | WY | Control | SY | Dex | Mid Inc | 0 | . | . | . |
| Population*Treatment*Age*LHSS*Spl | WY | Control | SY | Base | End Inc | 0 | . | . | . |
| Population*Treatment*Age*LHSS*Spl | WY | Control | SY | Base | Nestling | 0 | . | . | . |
| Population*Treatment*Age*LHSS*Spl | WY | Control | SY | Str | Nestling | 0 | . | . | . |
| Population*Treatment*Age*LHSS*Spl | WY | Control | SY | Dex | Nestling | 0 | . | . | . |
| Population*Treatment*Age*LHSS*Spl | WY | Predator | ASY | Base | Mid Inc | 0 | . | . | . |
| Population*Treatment*Age*LHSS*Spl | WY | Predator | ASY | Str | Mid Inc | 0 | . | . | . |
| Population*Treatment*Age*LHSS*Spl | WY | Predator | ASY | Dex | Mid Inc | 0 | . | . | . |
| Population*Treatment*Age*LHSS*Spl | WY | Predator | ASY | Base | End Inc | 0 | . | . | . |
| Population*Treatment*Age*LHSS*Spl | WY | Predator | ASY | Base | Nestling | 0 | . | . | . |
| Population*Treatment*Age*LHSS*Spl | WY | Predator | ASY | Str | Nestling | 0 | . | . | . |
| Population*Treatment*Age*LHSS*Spl | WY | Predator | ASY | Dex | Nestling | 0 | . | . | . |
| Population*Treatment*Age*LHSS*Spl | WY | Predator | SY | Base | Mid Inc | 0 | . | . | . |
| Population*Treatment*Age*LHSS*Spl | WY | Predator | SY | Str | Mid Inc | 0 | . | . | . |
| Population*Treatment*Age*LHSS*Spl | WY | Predator | SY | Dex | Mid Inc | 0 | . | . | . |
| Population*Treatment*Age*LHSS*Spl | WY | Predator | SY | Base | End Inc | 0 | . | . | . |
| Population*Treatment*Age*LHSS*Spl | WY | Predator | SY | Base | Nestling | 0 | . | . | . |
| Population*Treatment*Age*LHSS*Spl | WY | Predator | SY | Str | Nestling | 0 | . | . | . |
| Population*Treatment*Age*LHSS*Spl | WY | Predator | SY | Dex | Nestling | 0 | . | . | . |

Table S15: Parameter estimates, standard error and confidence interval for the generalized linear mixed model for females corticosterone levels with average temperature unpredictability as a continuous predictor. The model was fitted with a gamma law and individual and experimental year specified as random factors. Unpred = average temperature unpredictability, Spl = Sample, Base = Baseline, Str = stress-induced, dex = post-dex, LHSS = Life history substage, Mid Inc = Mid Incubation, End incubation = End incubation, Nestling = Nestling provisioning, Rel_CI = relative clutch initiation date, Mass = Body mass

| Effect | Treatment | Age | Sample | LHSS | Estimate | SE | Lower | Upper |
| --- | --- | --- | --- | --- | --- | --- | --- | --- |
|  |  |  |  |  |  |  |  |  |
| Intercept |  |  |  |  | 2.7037 | 0.6053 | 1.516 | 3.8914 |
| unpred |  |  |  |  | 2.0281 | 0.3599 | 1.3209 | 2.7352 |
| Age |  | ASY |  |  | -0.6386 | 0.6878 | -1.9882 | 0.711 |
| Age |  | SY |  |  | 0 | . | . | . |
| LHSS |  |  |  | Mid Inc | 0.8661 | 0.6176 | -0.3456 | 2.0777 |
| LHSS |  |  |  | End Inc | 0.5154 | 0.631 | -0.7225 | 1.7534 |
| LHSS |  |  |  | Nestling | 0 | . | . | . |
| Spl |  |  | Str |  | 2.4722 | 0.6732 | 1.1513 | 3.7931 |
| Spl |  |  | Dex |  | 2.286 | 0.6732 | 0.9651 | 3.6069 |
| Spl |  |  | Base |  | 0 | . | . | . |
| Treatment | Control |  |  |  | -0.3484 | 0.6856 | -1.6936 | 0.9969 |
| Treatment | Low_Tape |  |  |  | -0.4215 | 0.8215 | -2.0332 | 1.1902 |
| Treatment | Predator |  |  |  | 0 | . | . | . |
| Rel_CI |  |  |  |  | -0.01081 | 0.004287 | -0.01926 | -0.00237 |
| Mass |  |  |  |  | -0.1195 | 0.01583 | -0.1506 | -0.0884 |
| unpred*Spl | |  | Str |  | -0.3314 | 0.3968 | -1.1098 | 0.447 |
| unpred*Spl | |  | Dex |  | -1c.7866 | 0.3962 | -2.5639 | -1.0093 |
| unpred*Spl | |  | Base |  | 0 | . | . | . |
| Age*Spl |  | ASY | Str |  | 0.3782 | 0.8834 | -1.355 | 2.1115 |
| Age*Spl |  | ASY | Dex |  | 0.4121 | 0.8822 | -1.3189 | 2.143 |
| Age*Spl |  | ASY | Base |  | 0 | . | . | . |
| Age*Spl |  | SY | Str |  | 0 | . | . | . |
| Age*Spl |  | SY | Dex |  | 0 | . | . | . |
| Age*Spl |  | SY | Base |  | 0 | . | . | . |
| Treatment*Spl | Control |  | Str |  | -0.2668 | 0.8764 | -1.9863 | 1.4527 |
| Treatment*Spl | Control |  | Dex |  | 0.4285 | 0.8764 | -1.291 | 2.148 |
| Treatment*Spl | Control |  | Base |  | 0 | . | . | . |
| Treatment*Spl | Low_Tape |  | Str |  | 0.6314 | 1.0662 | -1.4606 | 2.7234 |
| Treatment*Spl | Low_Tape |  | Dex |  | 0.9718 | 1.0662 | -1.1202 | 3.0637 |
| Treatment*Spl | Low_Tape |  | Base |  | 0 | . | . | . |
| Treatment*Spl | Predator |  | Str |  | 0 | . | . | . |
| Treatment*Spl | Predator |  | Dex |  | 0 | . | . | . |
| Treatment*Spl | Predator |  | Base |  | 0 | . | . | . |
| LHSS*Spl | |  | Str | Mid Inc | -0.3609 | 0.8412 | -2.0113 | 1.2895 |
| LHSS*Spl | |  | Dex | Mid Inc | -0.9982 | 0.8412 | -2.6486 | 0.6523 |
| LHSS*Spl | |  | Base | Mid Inc | 0 | . | . | . |
| LHSS*Spl | |  | Base | End Inc | 0 | . | . | . |
| LHSS*Spl | |  | Str | Nestling | 0 | . | . | . |
| LHSS*Spl | |  | Dex | Nestling | 0 | . | . | . |
| LHSS*Spl | |  | Base | Nestling | 0 | . | . | . |
| unpred*Age*Spl | | ASY | Str |  | 0.7019 | 1.7447 | -2.7212 | 4.125 |
| unpred*Age*Spl | | ASY | Dex |  | 0.3433 | 1.7351 | -3.0609 | 3.7475 |
| unpred*Age*Spl | | ASY | Base |  | 0.7534 | 1.7262 | -2.6334 | 4.1402 |
| unpred*Age*Spl | | SY | Str |  | 0 | . | . | . |
| unpred*Age*Spl | | SY | Dex |  | 0 | . | . | . |
| unpred*Age*Spl | | SY | Base |  | 0 | . | . | . |
| unpred*Treatment*Spl | Control |  | Str |  | 1.8011 | 1.7598 | -1.6516 | 5.2538 |
| unpred*Treatment*Spl | Control |  | Dex |  | -0.6518 | 1.7598 | -4.1045 | 2.8009 |
| unpred*Treatment*Spl | Control |  | Base |  | -0.01793 | 1.7598 | -3.4706 | 3.4348 |
| unpred*Treatment*Spl | Low_Tape |  | Str |  | -1.0199 | 2.3017 | -5.5352 | 3.4954 |
| unpred*Treatment*Spl | Low_Tape |  | Dex |  | -2.6649 | 2.3017 | -7.1802 | 1.8504 |
| unpred*Treatment*Spl | Low_Tape |  | Base |  | 0.3428 | 2.3017 | -4.1725 | 4.8581 |
| unpred*Treatment*Spl | Predator |  | Str |  | 0 | . | . | . |
| unpred*Treatment*Spl | Predator |  | Dex |  | 0 | . | . | . |
| unpred*Treatment*Spl | Predator |  | Base |  | 0 | . | . | . |
| Treatment*Age*Spl | Control | ASY | Str |  | 0.9091 | 0.9423 | -0.94 | 2.7581 |
| Treatment*Age*Spl | Control | ASY | Dex |  | -0.3775 | 0.9323 | -2.2069 | 1.452 |
| Treatment*Age*Spl | Control | ASY | Base |  | 0.3238 | 0.9309 | -1.503 | 2.1505 |
| Treatment*Age*Spl | Control | SY | Str |  | 0 | . | . | . |
| Treatment*Age*Spl | Control | SY | Dex |  | 0 | . | . | . |
| Treatment*Age*Spl | Control | SY | Base |  | 0 | . | . | . |
| Treatment*Age*Spl | Low_Tape | ASY | Str |  | -0.2334 | 1.0806 | -2.3534 | 1.8866 |
| Treatment*Age*Spl | Low_Tape | ASY | Dex |  | -0.8391 | 1.0818 | -2.9616 | 1.2834 |
| Treatment*Age*Spl | Low_Tape | ASY | Base |  | 0.1762 | 1.0787 | -1.9403 | 2.2926 |
| Treatment*Age*Spl | Low_Tape | SY | Str |  | 0 | . | . | . |
| Treatment*Age*Spl | Low_Tape | SY | Dex |  | 0 | . | . | . |
| Treatment*Age*Spl | Low_Tape | SY | Base |  | 0 | . | . | . |
| Treatment*Age*Spl | Predator | ASY | Str |  | 0 | . | . | . |
| Treatment*Age*Spl | Predator | ASY | Dex |  | 0 | . | . | . |
| Treatment*Age*Spl | Predator | ASY | Base |  | 0 | . | . | . |
| Treatment*Age*Spl | Predator | SY | Str |  | 0 | . | . | . |
| Treatment*Age*Spl | Predator | SY | Dex |  | 0 | . | . | . |
| Treatment*Age*Spl | Predator | SY | Base |  | 0 | . | . | . |
| Treatment*LHSS*Spl | Control |  | Str | Mid Inc | 0.07135 | 0.8208 | -1.539 | 1.6817 |
| Treatment*LHSS*Spl | Control |  | Dex | Mid Inc | 0.08966 | 0.8208 | -1.5206 | 1.6999 |
| Treatment*LHSS*Spl | Control |  | Base | Mid Inc | -0.2491 | 0.8206 | -1.8589 | 1.3608 |
| Treatment*LHSS*Spl | Control |  | Base | End Inc | -0.4749 | 0.8403 | -2.1236 | 1.1738 |
| Treatment*LHSS*Spl | Control |  | Str | Nestling | 0 | . | . | . |
| Treatment*LHSS*Spl | Control |  | Dex | Nestling | 0 | . | . | . |
| Treatment*LHSS*Spl | Control |  | Base | Nestling | 0 | . | . | . |
| Treatment*LHSS*Spl | Low_Tape |  | Str | Mid Inc | -0.8529 | 0.9364 | -2.69 | 0.9842 |
| Treatment*LHSS*Spl | Low_Tape |  | Dex | Mid Inc | -0.4411 | 0.9364 | -2.2782 | 1.396 |
| Treatment*LHSS*Spl | Low_Tape |  | Base | Mid Inc | -1.0271 | 0.9364 | -2.8642 | 0.81 |
| Treatment*LHSS*Spl | Low_Tape |  | Base | End Inc | -0.5934 | 1.0049 | -2.565 | 1.3783 |
| Treatment*LHSS*Spl | Low_Tape |  | Str | Nestling | 0 | . | . | . |
| Treatment*LHSS*Spl | Low_Tape |  | Dex | Nestling | 0 | . | . | . |
| Treatment*LHSS*Spl | Low_Tape |  | Base | Nestling | 0 | . | . | . |
| Treatment*LHSS*Spl | Predator |  | Str | Mid Inc | 0 | . | . | . |
| Treatment*LHSS*Spl | Predator |  | Dex | Mid Inc | 0 | . | . | . |
| Treatment*LHSS*Spl | Predator |  | Base | Mid Inc | 0 | . | . | . |
| Treatment*LHSS*Spl | Predator |  | Base | End Inc | 0 | . | . | . |
| Treatment*LHSS*Spl | Predator |  | Str | Nestling | 0 | . | . | . |
| Treatment*LHSS*Spl | Predator |  | Dex | Nestling | 0 | . | . | . |
| Treatment*LHSS*Spl | Predator |  | Base | Nestling | 0 | . | . | . |
| unpred*LHSS*Spl | |  | Str | Mid Inc | -0.3773 | 1.5258 | -3.3708 | 2.6162 |
| unpred*LHSS*Spl | |  | Dex | Mid Inc | 0.7549 | 1.5258 | -2.2386 | 3.7484 |
| unpred*LHSS*Spl | |  | Base | Mid Inc | -2.361 | 1.5258 | -5.3545 | 0.6325 |
| unpred*LHSS*Spl | |  | Base | End Inc | -1.4756 | 1.5883 | -4.5918 | 1.6406 |
| unpred*LHSS*Spl | |  | Str | Nestling | 0 | . | . | . |
| unpred*LHSS*Spl | |  | Dex | Nestling | 0 | . | . | . |
| unpred*LHSS*Spl | |  | Base | Nestling | 0 | . | . | . |
| Age*LHSS*Spl | | ASY | Str | Mid Inc | -0.2322 | 0.818 | -1.837 | 1.3726 |
| Age*LHSS*Spl | | ASY | Dex | Mid Inc | 0.4571 | 0.817 | -1.1458 | 2.0599 |
| Age*LHSS*Spl | | ASY | Base | Mid Inc | -0.8731 | 0.8156 | -2.4732 | 0.727 |
| Age*LHSS*Spl | | ASY | Base | End Inc | 0.03521 | 0.8349 | -1.6027 | 1.6732 |
| Age*LHSS*Spl | | ASY | Str | Nestling | 0 | . | . | . |
| Age*LHSS*Spl | | ASY | Dex | Nestling | 0 | . | . | . |
| Age*LHSS*Spl | | ASY | Base | Nestling | 0 | . | . | . |
| Age*LHSS*Spl | | SY | Str | Mid Inc | 0 | . | . | . |
| Age*LHSS*Spl | | SY | Dex | Mid Inc | 0 | . | . | . |
| Age*LHSS*Spl | | SY | Base | Mid Inc | 0 | . | . | . |
| Age*LHSS*Spl | | SY | Base | End Inc | 0 | . | . | . |
| Age*LHSS*Spl | | SY | Str | Nestling | 0 | . | . | . |
| Age*LHSS*Spl | | SY | Dex | Nestling | 0 | . | . | . |
| Age*LHSS*Spl | | SY | Base | Nestling | 0 | . | . | . |
| unpred*Age*LHSS*Spl | | ASY | Str | Mid Inc | 0.4599 | 2.0466 | -3.5554 | 4.4751 |
| unpred*Age*LHSS*Spl | | ASY | Dex | Mid Inc | -1.0648 | 2.0399 | -5.0669 | 2.9373 |
| unpred*Age*LHSS*Spl | | ASY | Base | Mid Inc | 2.1135 | 2.0306 | -1.8704 | 6.0974 |
| unpred*Age*LHSS*Spl | | ASY | Base | End Inc | 0.1008 | 2.1136 | -4.046 | 4.2476 |
| unpred*Age*LHSS*Spl | | ASY | Str | Nestling | 0 | . | . | . |
| unpred*Age*LHSS*Spl | | ASY | Dex | Nestling | 0 | . | . | . |
| unpred*Age*LHSS*Spl | | ASY | Base | Nestling | 0 | . | . | . |
| unpred*Age*LHSS*Spl | | SY | Str | Mid Inc | 0 | . | . | . |
| unpred*Age*LHSS*Spl | | SY | Dex | Mid Inc | 0 | . | . | . |
| unpred*Age*LHSS*Spl | | SY | Base | Mid Inc | 0 | . | . | . |
| unpred*Age*LHSS*Spl | | SY | Base | End Inc | 0 | . | . | . |
| unpred*Age*LHSS*Spl | | SY | Str | Nestling | 0 | . | . | . |
| unpred*Age*LHSS*Spl | | SY | Dex | Nestling | 0 | . | . | . |
| unpred*Age*LHSS*Spl | | SY | Base | Nestling | 0 | . | . | . |
| unpred*Treatment*Age*Spl | Control | ASY | Str |  | -3.0396 | 2.393 | -7.7347 | 1.6556 |
| unpred*Treatment*Age*Spl | Control | ASY | Dex |  | 1.0667 | 2.3693 | -3.582 | 5.7155 |
| unpred*Treatment*Age*Spl | Control | ASY | Base |  | -0.4388 | 2.3628 | -5.0749 | 4.1973 |
| unpred*Treatment*Age*Spl | Control | SY | Str |  | 0 | . | . | . |
| unpred*Treatment*Age*Spl | Control | SY | Dex |  | 0 | . | . | . |
| unpred*Treatment*Age*Spl | Control | SY | Base |  | 0 | . | . | . |
| unpred*Treatment*Age*Spl | Low_Tape | ASY | Str |  | 0.5387 | 2.9666 | -5.2811 | 6.3585 |
| unpred*Treatment*Age*Spl | Low_Tape | ASY | Dex |  | 3.6631 | 2.9753 | -2.1738 | 9.5001 |
| unpred*Treatment*Age*Spl | Low_Tape | ASY | Base |  | 1.0999 | 2.9557 | -4.6985 | 6.8983 |
| unpred*Treatment*Age*Spl | Low_Tape | SY | Str |  | 0 | . | . | . |
| unpred*Treatment*Age*Spl | Low_Tape | SY | Dex |  | 0 | . | . | . |
| unpred*Treatment*Age*Spl | Low_Tape | SY | Base |  | 0 | . | . | . |
| unpred*Treatment*Age*Spl | Predator | ASY | Str |  | 0 | . | . | . |
| unpred*Treatment*Age*Spl | Predator | ASY | Dex |  | 0 | . | . | . |
| unpred*Treatment*Age*Spl | Predator | ASY | Base |  | 0 | . | . | . |
| unpred*Treatment*Age*Spl | Predator | SY | Str |  | 0 | . | . | . |
| unpred*Treatment*Age*Spl | Predator | SY | Dex |  | 0 | . | . | . |
| unpred*Treatment*Age*Spl | Predator | SY | Base |  | 0 | . | . | . |
| Treatment*Age*LHSS*Spl | Control | ASY | Str | Mid Inc | 0.3066 | 1.1323 | -1.9149 | 2.5281 |
| Treatment*Age*LHSS*Spl | Control | ASY | Dex | Mid Inc | 0.04367 | 1.1244 | -2.1623 | 2.2496 |
| Treatment*Age*LHSS*Spl | Control | ASY | Base | Mid Inc | -0.4161 | 1.1227 | -2.6188 | 1.7866 |
| Treatment*Age*LHSS*Spl | Control | ASY | Base | End Inc | 0.7823 | 1.1475 | -1.4691 | 3.0337 |
| Treatment*Age*LHSS*Spl | Control | ASY | Str | Nestling | 0 | . | . | . |
| Treatment*Age*LHSS*Spl | Control | ASY | Dex | Nestling | 0 | . | . | . |
| Treatment*Age*LHSS*Spl | Control | ASY | Base | Nestling | 0 | . | . | . |
| Treatment*Age*LHSS*Spl | Control | SY | Str | Mid Inc | 0 | . | . | . |
| Treatment*Age*LHSS*Spl | Control | SY | Dex | Mid Inc | 0 | . | . | . |
| Treatment*Age*LHSS*Spl | Control | SY | Base | Mid Inc | 0 | . | . | . |
| Treatment*Age*LHSS*Spl | Control | SY | Base | End Inc | 0 | . | . | . |
| Treatment*Age*LHSS*Spl | Control | SY | Str | Nestling | 0 | . | . | . |
| Treatment*Age*LHSS*Spl | Control | SY | Dex | Nestling | 0 | . | . | . |
| Treatment*Age*LHSS*Spl | Control | SY | Base | Nestling | 0 | . | . | . |
| Treatment*Age*LHSS*Spl | Low_Tape | ASY | Str | Mid Inc | 1.7236 | 1.2593 | -0.747 | 4.1941 |
| Treatment*Age*LHSS*Spl | Low_Tape | ASY | Dex | Mid Inc | -0.1786 | 1.2605 | -2.6516 | 2.2945 |
| Treatment*Age*LHSS*Spl | Low_Tape | ASY | Base | Mid Inc | 1.6571 | 1.2577 | -0.8104 | 4.1246 |
| Treatment*Age*LHSS*Spl | Low_Tape | ASY | Base | End Inc | 0.1158 | 1.3248 | -2.4835 | 2.7151 |
| Treatment*Age*LHSS*Spl | Low_Tape | ASY | Str | Nestling | 0 | . | . | . |
| Treatment*Age*LHSS*Spl | Low_Tape | ASY | Dex | Nestling | 0 | . | . | . |
| Treatment*Age*LHSS*Spl | Low_Tape | ASY | Base | Nestling | 0 | . | . | . |
| Treatment*Age*LHSS*Spl | Low_Tape | SY | Str | Mid Inc | 0 | . | . | . |
| Treatment*Age*LHSS*Spl | Low_Tape | SY | Dex | Mid Inc | 0 | . | . | . |
| Treatment*Age*LHSS*Spl | Low_Tape | SY | Base | Mid Inc | 0 | . | . | . |
| Treatment*Age*LHSS*Spl | Low_Tape | SY | Base | End Inc | 0 | . | . | . |
| Treatment*Age*LHSS*Spl | Low_Tape | SY | Str | Nestling | 0 | . | . | . |
| Treatment*Age*LHSS*Spl | Low_Tape | SY | Dex | Nestling | 0 | . | . | . |
| Treatment*Age*LHSS*Spl | Low_Tape | SY | Base | Nestling | 0 | . | . | . |
| Treatment*Age*LHSS*Spl | Predator | ASY | Str | Mid Inc | 0 | . | . | . |
| Treatment*Age*LHSS*Spl | Predator | ASY | Dex | Mid Inc | 0 | . | . | . |
| Treatment*Age*LHSS*Spl | Predator | ASY | Base | Mid Inc | 0 | . | . | . |
| Treatment*Age*LHSS*Spl | Predator | ASY | Base | End Inc | 0 | . | . | . |
| Treatment*Age*LHSS*Spl | Predator | ASY | Str | Nestling | 0 | . | . | . |
| Treatment*Age*LHSS*Spl | Predator | ASY | Dex | Nestling | 0 | . | . | . |
| Treatment*Age*LHSS*Spl | Predator | ASY | Base | Nestling | 0 | . | . | . |
| Treatment*Age*LHSS*Spl | Predator | SY | Str | Mid Inc | 0 | . | . | . |
| Treatment*Age*LHSS*Spl | Predator | SY | Dex | Mid Inc | 0 | . | . | . |
| Treatment*Age*LHSS*Spl | Predator | SY | Base | Mid Inc | 0 | . | . | . |
| Treatment*Age*LHSS*Spl | Predator | SY | Base | End Inc | 0 | . | . | . |
| Treatment*Age*LHSS*Spl | Predator | SY | Str | Nestling | 0 | . | . | . |
| Treatment*Age*LHSS*Spl | Predator | SY | Dex | Nestling | 0 | . | . | . |
| Treatment*Age*LHSS*Spl | Predator | SY | Base | Nestling | 0 | . | . | . |
| unpred*Treatment*Age*LHSS*Spl | Control | ASY | Str | Mid Inc | 0.06325 | 1.9387 | -3.7404 | 3.8669 |
| unpred*Treatment*Age*LHSS*Spl | Control | ASY | Dex | Mid Inc | -0.04023 | 1.914 | -3.7955 | 3.7151 |
| unpred*Treatment*Age*LHSS*Spl | Control | ASY | Base | Mid Inc | 3.2137 | 1.9011 | -0.5162 | 6.9435 |
| unpred*Treatment*Age*LHSS*Spl | Control | ASY | Base | End Inc | -0.5982 | 1.9622 | -4.448 | 3.2517 |
| unpred*Treatment*Age*LHSS*Spl | Control | ASY | Str | Nestling | 0 | . | . | . |
| unpred*Treatment*Age*LHSS*Spl | Control | ASY | Dex | Nestling | 0 | . | . | . |
| unpred*Treatment*Age*LHSS*Spl | Control | ASY | Base | Nestling | 0 | . | . | . |
| unpred*Treatment*Age*LHSS*Spl | Control | SY | Str | Mid Inc | -0.612 | 2.0893 | -4.7111 | 3.4871 |
| unpred*Treatment*Age*LHSS*Spl | Control | SY | Dex | Mid Inc | 0.3144 | 2.0871 | -3.7802 | 4.4091 |
| unpred*Treatment*Age*LHSS*Spl | Control | SY | Base | Mid Inc | 1.5205 | 2.087 | -2.5741 | 5.6151 |
| unpred*Treatment*Age*LHSS*Spl | Control | SY | Base | End Inc | 1.5824 | 2.1743 | -2.6835 | 5.8482 |
| unpred*Treatment*Age*LHSS*Spl | Control | SY | Str | Nestling | 0 | . | . | . |
| unpred*Treatment*Age*LHSS*Spl | Control | SY | Dex | Nestling | 0 | . | . | . |
| unpred*Treatment*Age*LHSS*Spl | Control | SY | Base | Nestling | 0 | . | . | . |
| unpred*Treatment*Age*LHSS*Spl | Low_Tape | ASY | Str | Mid Inc | -1.5354 | 2.2472 | -5.9442 | 2.8734 |
| unpred*Treatment*Age*LHSS*Spl | Low_Tape | ASY | Dex | Mid Inc | 2.06 | 2.2605 | -2.3749 | 6.495 |
| unpred*Treatment*Age*LHSS*Spl | Low_Tape | ASY | Base | Mid Inc | -1.5381 | 2.2326 | -5.9184 | 2.8421 |
| unpred*Treatment*Age*LHSS*Spl | Low_Tape | ASY | Base | End Inc | 1.2948 | 2.3159 | -3.2489 | 5.8386 |
| unpred*Treatment*Age*LHSS*Spl | Low_Tape | ASY | Str | Nestling | 0 | . | . | . |
| unpred*Treatment*Age*LHSS*Spl | Low_Tape | ASY | Dex | Nestling | 0 | . | . | . |
| unpred*Treatment*Age*LHSS*Spl | Low_Tape | ASY | Base | Nestling | 0 | . | . | . |
| unpred*Treatment*Age*LHSS*Spl | Low_Tape | SY | Str | Mid Inc | 2.5579 | 2.6196 | -2.5814 | 7.6972 |
| unpred*Treatment*Age*LHSS*Spl | Low_Tape | SY | Dex | Mid Inc | 2.5381 | 2.6196 | -2.6013 | 7.6774 |
| unpred*Treatment*Age*LHSS*Spl | Low_Tape | SY | Base | Mid Inc | 4.0761 | 2.6196 | -1.0632 | 9.2154 |
| unpred*Treatment*Age*LHSS*Spl | Low_Tape | SY | Base | End Inc | 2.3336 | 2.8687 | -3.2947 | 7.9618 |
| unpred*Treatment*Age*LHSS*Spl | Low_Tape | SY | Str | Nestling | 0 | . | . | . |
| unpred*Treatment*Age*LHSS*Spl | Low_Tape | SY | Dex | Nestling | 0 | . | . | . |
| unpred*Treatment*Age*LHSS*Spl | Low_Tape | SY | Base | Nestling | 0 | . | . | . |
| unpred*Treatment*Age*LHSS*Spl | Predator | ASY | Str | Mid Inc | 0 | . | . | . |
| unpred*Treatment*Age*LHSS*Spl | Predator | ASY | Dex | Mid Inc | 0 | . | . | . |
| unpred*Treatment*Age*LHSS*Spl | Predator | ASY | Base | Mid Inc | 0 | . | . | . |
| unpred*Treatment*Age*LHSS*Spl | Predator | ASY | Base | End Inc | 0 | . | . | . |
| unpred*Treatment*Age*LHSS*Spl | Predator | ASY | Str | Nestling | 0 | . | . | . |
| unpred*Treatment*Age*LHSS*Spl | Predator | ASY | Dex | Nestling | 0 | . | . | . |
| unpred*Treatment*Age*LHSS*Spl | Predator | ASY | Base | Nestling | 0 | . | . | . |
| unpred*Treatment*Age*LHSS*Spl | Predator | SY | Str | Mid Inc | 0 | . | . | . |
| unpred*Treatment*Age*LHSS*Spl | Predator | SY | Dex | Mid Inc | 0 | . | . | . |
| unpred*Treatment*Age*LHSS*Spl | Predator | SY | Base | Mid Inc | 0 | . | . | . |
| unpred*Treatment*Age*LHSS*Spl | Predator | SY | Base | End Inc | 0 | . | . | . |
| unpred*Treatment*Age*LHSS*Spl | Predator | SY | Str | Nestling | 0 | . | . | . |
| unpred*Treatment*Age*LHSS*Spl | Predator | SY | Dex | Nestling | 0 | . | . | . |
| unpred*Treatment*Age*LHSS*Spl | Predator | SY | Base | Nestling | 0 | . | . | . |

Table S16: Parameter estimates, standard error and confidence interval for the generalized linear mixed model for females corticosterone levels with total breeding season length as continuous predictor. The model was fitted with a gamma law and individual and experimental year specified as random factor and populations as random slope. Breedinglength = Total breeding season length, Spl = Sample, Base = Baseline, Str = stress-induced, dex = post-dex, LHSS = Life history substage, Mid Inc = Mid Incubation, End incubation = End incubation, Nestling = Nestling provisioning, Rel_CI = relative clutch initiation date, Mass = Body mass

| Effect | Treatment | Age | Sample | LHSS | | Estimate | | SE | Lower | | Upper | |  |
| --- | --- | --- | --- | --- | --- | --- | --- | --- | --- | --- | --- | --- | --- |
|  |  |  |  |  |  |  |  |  |  |  |  |  |  |
| Intercept |  |  |  | |  | 5.7431 | 0.8672 | | | 4.0418 | | 7.4444 | |
| breedinglength | |  |  | |  | -0.02904 | 0.01072 | | | -0.05007 | | -0.008 | |
| Age |  | ASY |  | |  | -0.5966 | 1.1085 | | | -2.7713 | | 1.578 | |
| Age |  | SY |  | |  | 0 | . | | | . | | . | |
| LHSS |  |  |  | | Mid Inc | -2.3665 | 0.9854 | | | -4.2997 | | -0.4333 | |
| LHSS |  |  |  | | End Inc | -1.6047 | 1.0232 | | | -3.6122 | | 0.4029 | |
| LHSS |  |  |  | | Nestling | 0 | . | | | . | | . | |
| Spl |  |  | Str | |  | 1.4418 | 1.0729 | | | -0.6633 | | 3.5469 | |
| Spl |  |  | Dex | |  | -0.877 | 1.0729 | | | -2.9821 | | 1.2281 | |
| Spl |  |  | Base | |  | 0 | . | | | . | | . | |
| Treatment | Control |  |  | |  | -1.077 | 1.126 | | | -3.286 | | 1.1321 | |
| Treatment | Low_Tape |  |  | |  | -1.1458 | 1.4592 | | | -4.0084 | | 1.7169 | |
| Treatment | Predator |  |  | |  | 0 | . | | | . | | . | |
| Rel_CI |  |  |  | |  | -0.0087 | 0.004199 | | | -0.01697 | | -0.00043 | |
| Mass |  |  |  | |  | -0.128 | 0.01453 | | | -0.1566 | | -0.09946 | |
| breedinglength*Spl | |  | Str | |  | 0.01163 | 0.01388 | | | -0.0156 | | 0.03887 | |
| breedinglength*Spl | |  | Dex | |  | 0.03096 | 0.01388 | | | 0.003732 | | 0.0582 | |
| breedinglength*Spl | |  | Base | |  | 0 | . | | | . | | . | |
| Age*Spl |  | ASY | Str | |  | 0.7664 | 1.4528 | | | -2.0841 | | 3.6169 | |
| Age*Spl |  | ASY | Dex | |  | 0.7684 | 1.4495 | | | -2.0755 | | 3.6123 | |
| Age*Spl |  | ASY | Base | |  | 0 | . | | | . | | . | |
| Age*Spl |  | SY | Str | |  | 0 | . | | | . | | . | |
| Age*Spl |  | SY | Dex | |  | 0 | . | | | . | | . | |
| Age*Spl |  | SY | Base | |  | 0 | . | | | . | | . | |
| Treatment*Spl | Control |  | Str | |  | 2.2242 | 1.4719 | | | -0.6636 | | 5.112 | |
| Treatment*Spl | Control |  | Dex | |  | 0.8031 | 1.4719 | | | -2.0847 | | 3.6909 | |
| Treatment*Spl | Control |  | Base | |  | 0 | . | | | . | | . | |
| Treatment*Spl | Low_Tape |  | Str | |  | -0.01203 | 1.9447 | | | -3.8276 | | 3.8036 | |
| Treatment*Spl | Low_Tape |  | Dex | |  | -0.7553 | 1.9447 | | | -4.5709 | | 3.0604 | |
| Treatment*Spl | Low_Tape |  | Base | |  | 0 | . | | | . | | . | |
| Treatment*Spl | Predator |  | Str | |  | 0 | . | | | . | | . | |
| Treatment*Spl | Predator |  | Dex | |  | 0 | . | | | . | | . | |
| Treatment*Spl | Predator |  | Base | |  | 0 | . | | | . | | . | |
| LHSS*Spl | |  | Str | | 1 | 2.1275 | 1.364 | | | -0.5488 | | 4.8037 | |
| LHSS*Spl | |  | Dex | | 1 | 3.2591 | 1.364 | | | 0.5828 | | 5.9353 | |
| LHSS*Spl | |  | Base | | 1 | 0 | . | | | . | | . | |
| LHSS*Spl | |  | Base | | 2 | 0 | . | | | . | | . | |
| LHSS*Spl | |  | Str | | 3 | 0 | . | | | . | | . | |
| LHSS*Spl | |  | Dex | | 3 | 0 | . | | | . | | . | |
| LHSS*Spl | |  | Base | | 3 | 0 | . | | | . | | . | |
| breedinglength*Age*Spl | | ASY | Str | |  | -0.00174 | 0.01434 | | | -0.02987 | | 0.02638 | |
| breedinglength*Age*Spl | | ASY | Dex | |  | -0.00319 | 0.01431 | | | -0.03126 | | 0.02488 | |
| breedinglength*Age*Spl | | ASY | Base | |  | 0.003991 | 0.01428 | | | -0.02402 | | 0.032 | |
| breedinglength*Age*Spl | | SY | Str | |  | 0 | . | | | . | | . | |
| breedinglength*Age*Spl | | SY | Dex | |  | 0 | . | | | . | | . | |
| breedinglength*Age*Spl | | SY | Base | |  | 0 | . | | | . | | . | |
| breedinglength*Treatment*Spl | Control |  | Str | |  | -0.01395 | 0.01433 | | | -0.04206 | | 0.01416 | |
| breedinglength*Treatment*Spl | Control |  | Dex | |  | 0.001544 | 0.01433 | | | -0.02657 | | 0.02966 | |
| breedinglength*Treatment*Spl | Control |  | Base | |  | 0.009718 | 0.01433 | | | -0.0184 | | 0.03783 | |
| breedinglength*Treatment*Spl | Low_Tape |  | Str | |  | 0.01295 | 0.01771 | | | -0.02179 | | 0.0477 | |
| breedinglength*Treatment*Spl | Low_Tape |  | Dex | |  | 0.01939 | 0.01771 | | | -0.01536 | | 0.05414 | |
| breedinglength*Treatment*Spl | Low_Tape |  | Base | |  | 0.01126 | 0.01771 | | | -0.02349 | | 0.04601 | |
| breedinglength*Treatment*Spl | Predator |  | Str | |  | 0 | . | | | . | | . | |
| breedinglength*Treatment*Spl | Predator |  | Dex | |  | 0 | . | | | . | | . | |
| breedinglength*Treatment*Spl | Predator |  | Base | |  | 0 | . | | | . | | . | |
| Treatment*Age*Spl | Control | ASY | Str | |  | -1.6002 | 1.5589 | | | -4.6585 | | 1.4581 | |
| Treatment*Age*Spl | Control | ASY | Dex | |  | 0.7531 | 1.5463 | | | -2.2805 | | 3.7866 | |
| Treatment*Age*Spl | Control | ASY | Base | |  | 1.0936 | 1.5427 | | | -1.9329 | | 4.12 | |
| Treatment*Age*Spl | Control | SY | Str | |  | 0 | . | | | . | | . | |
| Treatment*Age*Spl | Control | SY | Dex | |  | 0 | . | | | . | | . | |
| Treatment*Age*Spl | Control | SY | Base | |  | 0 | . | | | . | | . | |
| Treatment*Age*Spl | Low_Tape | ASY | Str | |  | 0.5229 | 1.8616 | | | -3.1291 | | 4.1749 | |
| Treatment*Age*Spl | Low_Tape | ASY | Dex | |  | 2.6054 | 1.8696 | | | -1.0622 | | 6.273 | |
| Treatment*Age*Spl | Low_Tape | ASY | Base | |  | 1.9019 | 1.857 | | | -1.741 | | 5.5448 | |
| Treatment*Age*Spl | Low_Tape | SY | Str | |  | 0 | . | | | . | | . | |
| Treatment*Age*Spl | Low_Tape | SY | Dex | |  | 0 | . | | | . | | . | |
| Treatment*Age*Spl | Low_Tape | SY | Base | |  | 0 | . | | | . | | . | |
| Treatment*Age*Spl | Predator | ASY | Str | |  | 0 | . | | | . | | . | |
| Treatment*Age*Spl | Predator | ASY | Dex | |  | 0 | . | | | . | | . | |
| Treatment*Age*Spl | Predator | ASY | Base | |  | 0 | . | | | . | | . | |
| Treatment*Age*Spl | Predator | SY | Str | |  | 0 | . | | | . | | . | |
| Treatment*Age*Spl | Predator | SY | Dex | |  | 0 | . | | | . | | . | |
| Treatment*Age*Spl | Predator | SY | Base | |  | 0 | . | | | . | | . | |
| Treatment*LHSS*Spl | Control |  | Str | | Mid Inc | -0.3761 | 1.3772 | | | -3.0781 | | 2.3259 | |
| Treatment*LHSS*Spl | Control |  | Dex | | Mid Inc | 0.3398 | 1.3735 | | | -2.355 | | 3.0346 | |
| Treatment*LHSS*Spl | Control |  | Base | | Mid Inc | 2.0972 | 1.373 | | | -0.5966 | | 4.791 | |
| Treatment*LHSS*Spl | Control |  | Base | | End Inc | 2.0307 | 1.4295 | | | -0.774 | | 4.8353 | |
| Treatment*LHSS*Spl | Control |  | Str | | Nestling | 0 | . | | | . | | . | |
| Treatment*LHSS*Spl | Control |  | Dex | | Nestling | 0 | . | | | . | | . | |
| Treatment*LHSS*Spl | Control |  | Base | | Nestling | 0 | . | | | . | | . | |
| Treatment*LHSS*Spl | Low_Tape |  | Str | | Mid Inc | 1.9519 | 1.6848 | | | -1.3534 | | 5.2573 | |
| Treatment*LHSS*Spl | Low_Tape |  | Dex | | Mid Inc | 1.6525 | 1.6848 | | | -1.6529 | | 4.9578 | |
| Treatment*LHSS*Spl | Low_Tape |  | Base | | Mid Inc | 3.8219 | 1.6848 | | | 0.5165 | | 7.1272 | |
| Treatment*LHSS*Spl | Low_Tape |  | Base | | End Inc | 2.35 | 1.8549 | | | -1.2892 | | 5.9892 | |
| Treatment*LHSS*Spl | Low_Tape |  | Str | | Nestling | 0 | . | | | . | | . | |
| Treatment*LHSS*Spl | Low_Tape |  | Dex | | Nestling | 0 | . | | | . | | . | |
| Treatment*LHSS*Spl | Low_Tape |  | Base | | Nestling | 0 | . | | | . | | . | |
| Treatment*LHSS*Spl | Predator |  | Str | | Mid Inc | 0 | . | | | . | | . | |
| Treatment*LHSS*Spl | Predator |  | Dex | | Mid Inc | 0 | . | | | . | | . | |
| Treatment*LHSS*Spl | Predator |  | Base | | Mid Inc | 0 | . | | | . | | . | |
| Treatment*LHSS*Spl | Predator |  | Base | | End Inc | 0 | . | | | . | | . | |
| Treatment*LHSS*Spl | Predator |  | Str | | Nestling | 0 | . | | | . | | . | |
| Treatment*LHSS*Spl | Predator |  | Dex | | Nestling | 0 | . | | | . | | . | |
| Treatment*LHSS*Spl | Predator |  | Base | | Nestling | 0 | . | | | . | | . | |
| breedinglength*LHSS*Spl | | | Str | | Mid Inc | 0.008398 | 0.01278 | | | -0.01667 | | 0.03346 | |
| breedinglength*LHSS*Spl | | | Dex | | Mid Inc | -0.00922 | 0.01278 | | | -0.03429 | | 0.01584 | |
| breedinglength*LHSS*Spl | | | Baseline | | Mid Inc | 0.03101 | 0.01278 | | | 0.005948 | | 0.05608 | |
| breedinglength*LHSS*Spl | | | Baseline | | End Inc | 0.02104 | 0.01314 | | | -0.00474 | | 0.04683 | |
| breedinglength*LHSS*Spl | | | Str | | Nestling | 0 | . | | | . | | . | |
| breedinglength*LHSS*Spl | | | Dex | | Nestling | 0 | . | | | . | | . | |
| breedinglength*LHSS*Spl | | | Baseline | | Nestling | 0 | . | | | . | | . | |
| Age*LHSS*Spl | | ASY | Str | | Mid Inc | 0.4357 | 1.3497 | | | -2.2123 | | 3.0837 | |
| Age*LHSS*Spl | | ASY | Dex | | Mid Inc | -0.9926 | 1.3471 | | | -3.6355 | | 1.6503 | |
| Age*LHSS*Spl | | ASY | Base | | Mid Inc | 2.8478 | 1.3428 | | | -0.7868 | | 4.4824 | |
| Age*LHSS*Spl | | ASY | Base | | End Inc | 0.7777 | 1.3899 | | | -1.9492 | | 3.5046 | |
| Age*LHSS*Spl | | ASY | Str | | Nestling | 0 | . | | | . | | . | |
| Age*LHSS*Spl | | ASY | Dex | | Nestling | 0 | . | | | . | | . | |
| Age*LHSS*Spl | | ASY | Base | | Nestling | 0 | . | | | . | | . | |
| Age*LHSS*Spl | | SY | Str | | Mid Inc | 0 | . | | | . | | . | |
| Age*LHSS*Spl | | SY | Dex | | Mid Inc | 0 | . | | | . | | . | |
| Age*LHSS*Spl | | SY | Base | | Mid Inc | 0 | . | | | . | | . | |
| Age*LHSS*Spl | | SY | Base | | End Inc | 0 | . | | | . | | . | |
| Age*LHSS*Spl | | SY | Str | | Nestling | 0 | . | | | . | | . | |
| Age*LHSS*Spl | | SY | Dex | | Nestling | 0 | . | | | . | | . | |
| Age*LHSS*Spl | | SY | Base | | Nestling | 0 | . | | | . | | . | |
| breedinglength*Age*LHSS*Spl | | ASY | Str | | Mid Inc | -0.00646 | 0.0173 | | | -0.0404 | | 0.02748 | |
| breedinglength*Age*LHSS*Spl | | ASY | Dex | | Mid Inc | 0.01328 | 0.01728 | | | -0.02062 | | 0.04718 | |
| breedinglength*Age*LHSS*Spl | | ASY | Base | | Mid Inc | -0.02524 | 0.01725 | | | -0.05907 | | 0.008601 | |
| breedinglength*Age*LHSS*Spl | | ASY | Base | | End Inc | -0.00988 | 0.01768 | | | -0.04457 | | 0.02481 | |
| breedinglength*Age*LHSS*Spl | | ASY | Str | | Nestling | 0 | . | | | . | | . | |
| breedinglength*Age*LHSS*Spl | | ASY | Dex | | Nestling | 0 | . | | | . | | . | |
| breedinglength*Age*LHSS*Spl | | ASY | Base | | Nestling | 0 | . | | | . | | . | |
| breedinglength*Age*LHSS*Spl | | SY | Str | | Mid Inc | 0 | . | | | . | | . | |
| breedinglength*Age*LHSS*Spl | | SY | Dex | | Mid Inc | 0 | . | | | . | | . | |
| breedinglength*Age*LHSS*Spl | | SY | Base | | Mid Inc | 0 | . | | | . | | . | |
| breedinglength*Age*LHSS*Spl | | SY | Base | | End Inc | 0 | . | | | . | | . | |
| breedinglength*Age*LHSS*Spl | | SY | Str | | Nestling | 0 | . | | | . | | . | |
| breedinglength*Age*LHSS*Spl | | SY | Dex | | Nestling | 0 | . | | | . | | . | |
| breedinglength*Age*LHSS*Spl | | SY | Base | | Nestling | 0 | . | | | . | | . | |
| breedinglength*Treatment*Age*Spl | Control | ASY | Str | |  | 0.01757 | 0.01986 | | | -0.02139 | | 0.05653 | |
| breedinglength*Treatment*Age*Spl | Control | ASY | Dex | |  | -0.00958 | 0.01968 | | | -0.04819 | | 0.02903 | |
| breedinglength*Treatment*Age*Spl | Control | ASY | Base | |  | -0.01259 | 0.01965 | | | -0.05115 | | 0.02597 | |
| breedinglength*Treatment*Age*Spl | Control | SY | Str | |  | 0 | . | | | . | | . | |
| breedinglength*Treatment*Age*Spl | Control | SY | Dex | |  | 0 | . | | | . | | . | |
| breedinglength*Treatment*Age*Spl | Control | SY | Base | |  | 0 | . | | | . | | . | |
| breedinglength*Treatment*Age*Spl | Low_Tape | ASY | Str | |  | -0.00762 | 0.0229 | | | -0.05255 | | 0.03731 | |
| breedinglength*Treatment*Age*Spl | Low_Tape | ASY | Dex | |  | -0.02747 | 0.02298 | | | -0.07255 | | 0.01761 | |
| breedinglength*Treatment*Age*Spl | Low_Tape | ASY | Base | |  | -0.018 | 0.02287 | | | -0.06286 | | 0.02686 | |
| breedinglength*Treatment*Age*Spl | Low_Tape | SY | Str | |  | 0 | . | | | . | | . | |
| breedinglength*Treatment*Age*Spl | Low_Tape | SY | Dex | |  | 0 | . | | | . | | . | |
| breedinglength*Treatment*Age*Spl | Low_Tape | SY | Base | |  | 0 | . | | | . | | . | |
| breedinglength*Treatment*Age*Spl | Predator | ASY | Str | |  | 0 | . | | | . | | . | |
| breedinglength*Treatment*Age*Spl | Predator | ASY | Dex | |  | 0 | . | | | . | | . | |
| breedinglength*Treatment*Age*Spl | Predator | ASY | Base | |  | 0 | . | | | . | | . | |
| breedinglength*Treatment*Age*Spl | Predator | SY | Str | |  | 0 | . | | | . | | . | |
| breedinglength*Treatment*Age*Spl | Predator | SY | Dex | |  | 0 | . | | | . | | . | |
| breedinglength*Treatment*Age*Spl | Predator | SY | Base | |  | 0 | . | | | . | | . | |
| Treatment*Age*LHSS*Spl | Control | ASY | Str | | Mid Inc | 0.2639 | 1.9049 | | | -3.4734 | | 4.0012 | |
| Treatment*Age*LHSS*Spl | Control | ASY | Dex | | Mid Inc | -0.2263 | 1.8951 | | | -3.9443 | | 3.4917 | |
| Treatment*Age*LHSS*Spl | Control | ASY | Base | | Mid Inc | 0.4656 | 1.8882 | | | -3.239 | | 4.1701 | |
| Treatment*Age*LHSS*Spl | Control | ASY | Base | | End Inc | -2.5902 | 1.9586 | | | -6.4329 | | 1.2524 | |
| Treatment*Age*LHSS*Spl | Control | ASY | Str | | Nestling | 0 | . | | | . | | . | |
| Treatment*Age*LHSS*Spl | Control | ASY | Dex | | Nestling | 0 | . | | | . | | . | |
| Treatment*Age*LHSS*Spl | Control | ASY | Base | | Nestling | 0 | . | | | . | | . | |
| Treatment*Age*LHSS*Spl | Control | SY | Str | | Mid Inc | 0 | . | | | . | | . | |
| Treatment*Age*LHSS*Spl | Control | SY | Dex | | Mid Inc | 0 | . | | | . | | . | |
| Treatment*Age*LHSS*Spl | Control | SY | Base | | Mid Inc | 0 | . | | | . | | . | |
| Treatment*Age*LHSS*Spl | Control | SY | Base | | End Inc | 0 | . | | | . | | . | |
| Treatment*Age*LHSS*Spl | Control | SY | Str | | Nestling | 0 | . | | | . | | . | |
| Treatment*Age*LHSS*Spl | Control | SY | Dex | | Nestling | 0 | . | | | . | | . | |
| Treatment*Age*LHSS*Spl | Control | SY | Base | | Nestling | 0 | . | | | . | | . | |
| Treatment*Age*LHSS*Spl | Low_Tape | ASY | Str | | Mid Inc | -2.4778 | 2.2006 | | | -6.7951 | | 1.8395 | |
| Treatment*Age*LHSS*Spl | Low_Tape | ASY | Dex | | Mid Inc | -0.2931 | 2.2081 | | | -4.6252 | | 4.039 | |
| Treatment*Age*LHSS*Spl | Low_Tape | ASY | Base | | Mid Inc | -4.4703 | 2.1964 | | | -8.7793 | | -0.1612 | |
| Treatment*Age*LHSS*Spl | Low_Tape | ASY | Base | | End Inc | -1.5453 | 2.3597 | | | -6.175 | | 3.0845 | |
| Treatment*Age*LHSS*Spl | Low_Tape | ASY | Str | | Nestling | 0 | . | | | . | | . | |
| Treatment*Age*LHSS*Spl | Low_Tape | ASY | Dex | | Nestling | 0 | . | | | . | | . | |
| Treatment*Age*LHSS*Spl | Low_Tape | ASY | Base | | Nestling | 0 | . | | | . | | . | |
| Treatment*Age*LHSS*Spl | Low_Tape | SY | Str | | Mid Inc | 0 | . | | | . | | . | |
| Treatment*Age*LHSS*Spl | Low_Tape | SY | Dex | | Mid Inc | 0 | . | | | . | | . | |
| Treatment*Age*LHSS*Spl | Low_Tape | SY | Base | | Mid Inc | 0 | . | | | . | | . | |
| Treatment*Age*LHSS*Spl | Low_Tape | SY | Base | | End Inc | 0 | . | | | . | | . | |
| Treatment*Age*LHSS*Spl | Low_Tape | SY | Str | | Nestling | 0 | . | | | . | | . | |
| Treatment*Age*LHSS*Spl | Low_Tape | SY | Dex | | Nestling | 0 | . | | | . | | . | |
| Treatment*Age*LHSS*Spl | Low_Tape | SY | Base | | Nestling | 0 | . | | | . | | . | |
| Treatment*Age*LHSS*Spl | Predator | ASY | Str | | Mid Inc | 0 | . | | | . | | . | |
| Treatment*Age*LHSS*Spl | Predator | ASY | Dex | | Mid Inc | 0 | . | | | . | | . | |
| Treatment*Age*LHSS*Spl | Predator | ASY | Base | | Mid Inc | 0 | . | | | . | | . | |
| Treatment*Age*LHSS*Spl | Predator | ASY | Base | | End Inc | 0 | . | | | . | | . | |
| Treatment*Age*LHSS*Spl | Predator | ASY | Str | | Nestling | 0 | . | | | . | | . | |
| Treatment*Age*LHSS*Spl | Predator | ASY | Dex | | Nestling | 0 | . | | | . | | . | |
| Treatment*Age*LHSS*Spl | Predator | ASY | Base | | Nestling | 0 | . | | | . | | . | |
| Treatment*Age*LHSS*Spl | Predator | SY | Str | | Mid Inc | 0 | . | | | . | | . | |
| Treatment*Age*LHSS*Spl | Predator | SY | Dex | | Mid Inc | 0 | . | | | . | | . | |
| Treatment*Age*LHSS*Spl | Predator | SY | Base | | Mid Inc | 0 | . | | | . | | . | |
| Treatment*Age*LHSS*Spl | Predator | SY | Base | | End Inc | 0 | . | | | . | | . | |
| Treatment*Age*LHSS*Spl | Predator | SY | Str | | Nestling | 0 | . | | | . | | . | |
| Treatment*Age*LHSS*Spl | Predator | SY | Dex | | Nestling | 0 | . | | | . | | . | |
| Treatment*Age*LHSS*Spl | Predator | SY | Base | | Nestling | 0 | . | | | . | | . | |
| breedinglength*Treatment*Age*LHSS*Spl | Control | ASY | Str | | Mid Inc | 0.006362 | 0.0168 | | | -0.02661 | | 0.03933 | |
| breedinglength*Treatment*Age*LHSS*Spl | Control | ASY | Dex | | Mid Inc | 0.000093 | 0.01663 | | | -0.03253 | | 0.03272 | |
| breedinglength*Treatment*Age*LHSS*Spl | Control | ASY | Base | | Mid Inc | -0.02584 | 0.01655 | | | -0.05832 | | 0.006635 | |
| breedinglength*Treatment*Age*LHSS*Spl | Control | ASY | Base | | End Inc | 0.008332 | 0.01695 | | | -0.02492 | | 0.04158 | |
| breedinglength*Treatment*Age*LHSS*Spl | Control | ASY | Str | | Nestling | 0 | . | | | . | | . | |
| breedinglength*Treatment*Age*LHSS*Spl | Control | ASY | Dex | | Nestling | 0 | . | | | . | | . | |
| breedinglength*Treatment*Age*LHSS*Spl | Control | ASY | Base | | Nestling | 0 | . | | | . | | . | |
| breedinglength*Treatment*Age*LHSS*Spl | Control | SY | Str | | Mid Inc | 0.002619 | 0.0175 | | | -0.03171 | | 0.03695 | |
| breedinglength*Treatment*Age*LHSS*Spl | Control | SY | Dex | | Mid Inc | -0.00194 | 0.01746 | | | -0.0362 | | 0.03232 | |
| breedinglength*Treatment*Age*LHSS*Spl | Control | SY | Base | | Mid Inc | -0.02331 | 0.01746 | | | -0.05756 | | 0.01095 | |
| breedinglength*Treatment*Age*LHSS*Spl | Control | SY | Base | | End Inc | -0.02525 | 0.01799 | | | -0.06055 | | 0.01006 | |
| breedinglength*Treatment*Age*LHSS*Spl | Control | SY | Str | | Nestling | 0 | . | | | . | | . | |
| breedinglength*Treatment*Age*LHSS*Spl | Control | SY | Dex | | Nestling | 0 | . | | | . | | . | |
| breedinglength*Treatment*Age*LHSS*Spl | Control | SY | Base | | Nestling | 0 | . | | | . | | . | |
| breedinglength*Treatment*Age*LHSS*Spl | Low_Tape | ASY | Str | | Mid Inc | 0.01056 | 0.01769 | | | -0.02415 | | 0.04526 | |
| breedinglength*Treatment*Age*LHSS*Spl | Low_Tape | ASY | Dex | | Mid Inc | -0.01571 | 0.0178 | | | -0.05063 | | 0.0192 | |
| breedinglength*Treatment*Age*LHSS*Spl | Low_Tape | ASY | Base | | Mid Inc | 0.009161 | 0.01764 | | | -0.02545 | | 0.04377 | |
| breedinglength*Treatment*Age*LHSS*Spl | Low_Tape | ASY | Base | | End Inc | -0.01028 | 0.01816 | | | -0.04592 | | 0.02536 | |
| breedinglength*Treatment*Age*LHSS*Spl | Low_Tape | ASY | Str | | Nestling | 0 | . | | | . | | . | |
| breedinglength*Treatment*Age*LHSS*Spl | Low_Tape | ASY | Dex | | Nestling | 0 | . | | | . | | . | |
| breedinglength*Treatment*Age*LHSS*Spl | Low_Tape | ASY | Base | | Nestling | 0 | . | | | . | | . | |
| breedinglength*Treatment*Age*LHSS*Spl | Low_Tape | SY | Str | | Mid Inc | -0.02463 | 0.02042 | | | -0.06469 | | 0.01543 | |
| breedinglength*Treatment*Age*LHSS*Spl | Low_Tape | SY | Dex | | Mid Inc | -0.01543 | 0.02042 | | | -0.05549 | | 0.02463 | |
| breedinglength*Treatment*Age*LHSS*Spl | Low_Tape | SY | Base | | Mid Inc | -0.04367 | 0.02042 | | | -0.08374 | | -0.00361 | |
| breedinglength*Treatment*Age*LHSS*Spl | Low_Tape | SY | Base | | End Inc | -0.02755 | 0.02221 | | | -0.07112 | | 0.01602 | |
| breedinglength*Treatment*Age*LHSS*Spl | Low_Tape | SY | Str | | Nestling | 0 | . | | | . | | . | |
| breedinglength*Treatment*Age*LHSS*Spl | Low_Tape | SY | Dex | | Nestling | 0 | . | | | . | | . | |
| breedinglength*Treatment*Age*LHSS*Spl | Low_Tape | SY | Base | | Nestling | 0 | . | | | . | | . | |
| breedinglength*Treatment*Age*LHSS*Spl | Predator | ASY | Str | | Mid Inc | 0 | . | | | . | | . | |
| breedinglength*Treatment*Age*LHSS*Spl | Predator | ASY | Dex | | Mid Inc | 0 | . | | | . | | . | |
| breedinglength*Treatment*Age*LHSS*Spl | Predator | ASY | Base | | Mid Inc | 0 | . | | | . | | . | |
| breedinglength*Treatment*Age*LHSS*Spl | Predator | ASY | Base | | End Inc | 0 | . | | | . | | . | |
| breedinglength*Treatment*Age*LHSS*Spl | Predator | ASY | Str | | Nestling | 0 | . | | | . | | . | |
| breedinglength*Treatment*Age*LHSS*Spl | Predator | ASY | Dex | | Nestling | 0 | . | | | . | | . | |
| breedinglength*Treatment*Age*LHSS*Spl | Predator | ASY | Base | | Nestling | 0 | . | | | . | | . | |
| breedinglength*Treatment*Age*LHSS*Spl | Predator | SY | Str | | Mid Inc | 0 | . | | | . | | . | |
| breedinglength*Treatment*Age*LHSS*Spl | Predator | SY | Dex | | Mid Inc | 0 | . | | | . | | . | |
| breedinglength*Treatment*Age*LHSS*Spl | Predator | SY | Base | | Mid Inc | 0 | . | | | . | | . | |
| breedinglength*Treatment*Age*LHSS*Spl | Predator | SY | Base | | End Inc | 0 | . | | | . | | . | |
| breedinglength*Treatment*Age*LHSS*Spl | Predator | SY | Str | | Nestling | 0 | . | | | . | | . | |
| breedinglength*Treatment*Age*LHSS*Spl | Predator | SY | Dex | | Nestling | 0 | . | | | . | | . | |
| breedinglength*Treatment*Age*LHSS*Spl | Predator | SY | Base | | Nestling | 0 | . | | | . | | . | |

Table S17: Parameter estimates, standard error and confidence interval for the generalized linear mixed model for females stress response. The model was fitted with a gamma law and individual and experimental year specified as random factor and populations as random slope. Spl = Sample, Base = Baseline, Str = stress-induced, dex = post-dex, LHSS = Life history substage, Mid Inc = Mid Incubation, End incubation = End incubation, Nestling = Nestling provisioning, Rel_CI = relative clutch initiation date, Mass = Body mass

| Effect | Population | Treatment | Age | LHSS | Estimate | SE | Lower | Upper |
| --- | --- | --- | --- | --- | --- | --- | --- | --- |
|  |  |  |  |  |  |  |  |  |
| Intercept |  |  |  |  | 5.9173 | 0.5487 | 4.8383 | 6.9963 |
| Population | AK |  |  |  | 0.9776 | 0.3235 | 0.3409 | 1.6144 |
| Population | NY |  |  |  | -0.1005 | 0.3738 | -0.8369 | 0.6358 |
| Population | TN |  |  |  | 0.2282 | 0.3723 | -0.5045 | 0.9609 |
| Population | WY |  |  |  | 0 | . | . | . |
| Age |  |  | ASY |  | 0.3874 | 0.3701 | -0.3421 | 1.1168 |
| Age |  |  | SY |  | 0 | . | . | . |
| LHSS |  |  |  | Mid Inc | 0.958 | 0.2724 | 0.4215 | 1.4945 |
| LHSS |  |  |  | Nestling | 0 | . | . | . |
| Treatment | | Control |  |  | 0.04284 | 0.405 | -0.7556 | 0.8412 |
| Treatment | | Low_Tape |  |  | -0.03226 | 0.3725 | -0.7671 | 0.7026 |
| Treatment | | Predator |  |  | 0 | . | . | . |
| Rel_CI |  |  |  |  | -0.01244 | 0.006262 | -0.02477 | -0.00012 |
| Mass |  |  |  |  | -0.1605 | 0.0252 | -0.2101 | -0.111 |
| Population*Age | AK |  | ASY |  | -0.5771 | 0.4455 | -1.4539 | 0.2998 |
| Population*Age | AK |  | SY |  | 0 | . | . | . |
| Population*Age | NY |  | ASY |  | -0.149 | 0.5317 | -1.1969 | 0.8989 |
| Population*Age | NY |  | SY |  | 0 | . | . | . |
| Population*Age | TN |  | ASY |  | -0.4752 | 0.5011 | -1.4614 | 0.511 |
| Population*Age | TN |  | SY |  | 0 | . | . | . |
| Population*Age | WY |  | ASY |  | 0 | . | . | . |
| Population*Age | WY |  | SY |  | 0 | . | . | . |
| Population*LHSS | AK |  |  | Mid Inc | -0.9037 | 0.3499 | -1.5932 | -0.2143 |
| Population*LHSS | AK |  |  | Nestling | 0 | . | . | . |
| Population*LHSS | NY |  |  | Mid Inc | -0.1799 | 0.4163 | -1.0002 | 0.6404 |
| Population*LHSS | NY |  |  | Nestling | 0 | . | . | . |
| Population*LHSS | TN |  |  | Mid Inc | -0.3557 | 0.3904 | -1.1245 | 0.4132 |
| Population*LHSS | TN |  |  | Nestling | 0 | . | . | . |
| Population*LHSS | WY |  |  | Mid Inc | 0 | . | . | . |
| Population*LHSS | WY |  |  | Nestling | 0 | . | . | . |
| Population*Treatment | AK | Control |  |  | 0.1129 | 0.4847 | -0.8413 | 1.0672 |
| Population*Treatment | AK | Low_Tape |  |  | -0.5553 | 0.503 | -1.5454 | 0.4349 |
| Population*Treatment | AK | Predator |  |  | 0 | . | . | . |
| Population*Treatment | NY | Control |  |  | 0.5275 | 0.5635 | -0.5825 | 1.6375 |
| Population*Treatment | NY | Low_Tape |  |  | 0.4783 | 0.7251 | -0.9524 | 1.909 |
| Population*Treatment | NY | Predator |  |  | 0 | . | . | . |
| Population*Treatment | TN | Control |  |  | -0.4516 | 0.5258 | -1.4865 | 0.5833 |
| Population*Treatment | TN | Low_Tape |  |  | 0 | . | . | . |
| Population*Treatment | TN | Predator |  |  | 0 | . | . | . |
| Population*Treatment | WY | Control |  |  | 0 | . | . | . |
| Population*Treatment | WY | Predator |  |  | 0 | . | . | . |
| Age*LHSS | |  | ASY | 1 | -0.3359 | 0.3702 | -1.065 | 0.3933 |
| Age*LHSS | |  | ASY | 3 | 0 | . | . | . |
| Age*LHSS | |  | SY | 1 | 0 | . | . | . |
| Age*LHSS | |  | SY | 3 | 0 | . | . | . |
| Treatment*Age | | Control | ASY |  | -0.4846 | 0.5113 | -1.4928 | 0.5236 |
| Treatment*Age | | Control | SY |  | 0 | . | . | . |
| Treatment*Age | | Low_Tape | ASY |  | -0.109 | 0.5 | -1.0964 | 0.8784 |
| Treatment*Age | | Low_Tape | SY |  | 0 | . | . | . |
| Treatment*Age | | Predator | ASY |  | 0 | . | . | . |
| Treatment*Age | | Predator | SY |  | 0 | . | . | . |
| Treatment*LHSS | | Control |  | Mid Inc | -0.1215 | 0.4062 | -0.9216 | 0.6786 |
| Treatment*LHSS | | Control |  | Nestling | 0 | . | . | . |
| Treatment*LHSS | | Low_Tape |  | Mid Inc | -0.3749 | 0.3845 | -1.1325 | 0.3826 |
| Treatment*LHSS | | Low_Tape |  | Nestling | 0 | . | . | . |
| Treatment*LHSS | | Predator |  | Mid Inc | 0 | . | . | . |
| Treatment*LHSS | | Predator |  | Nestling | 0 | . | . | . |
| Population*Age*LHSS | AK |  | ASY | Mid Inc | 0.4432 | 0.4852 | -0.5128 | 1.3992 |
| Population*Age*LHSS | AK |  | ASY | Nestling | 0 | . | . | . |
| Population*Age*LHSS | AK |  | SY | Mid Inc | 0 | . | . | . |
| Population*Age*LHSS | AK |  | SY | Nestling | 0 | . | . | . |
| Population*Age*LHSS | NY |  | ASY | Mid Inc | -0.00871 | 0.5895 | -1.1702 | 1.1528 |
| Population*Age*LHSS | NY |  | ASY | Nestling | 0 | . | . | . |
| Population*Age*LHSS | NY |  | SY | Mid Inc | 0 | . | . | . |
| Population*Age*LHSS | NY |  | SY | Nestling | 0 | . | . | . |
| Population*Age*LHSS | TN |  | ASY | Mid Inc | 0.2182 | 0.5104 | -0.7871 | 1.2235 |
| Population*Age*LHSS | TN |  | ASY | Nestling | 0 | . | . | . |
| Population*Age*LHSS | TN |  | SY | Mid Inc | 0 | . | . | . |
| Population*Age*LHSS | TN |  | SY | Nestling | 0 | . | . | . |
| Population*Age*LHSS | WY |  | ASY | Mid Inc | 0 | . | . | . |
| Population*Age*LHSS | WY |  | ASY | Nestling | 0 | . | . | . |
| Population*Age*LHSS | WY |  | SY | Mid Inc | 0 | . | . | . |
| Population*Age*LHSS | WY |  | SY | Nestling | 0 | . | . | . |
| Population*Treatment*Age | AK | Control | ASY |  | 0.4542 | 0.635 | -0.7957 | 1.7042 |
| Population*Treatment*Age | AK | Control | SY |  | 0 | . | . | . |
| Population*Treatment*Age | AK | Low_Tape | ASY |  | 0.2258 | 0.6604 | -1.0746 | 1.5261 |
| Population*Treatment*Age | AK | Low_Tape | SY |  | 0 | . | . | . |
| Population*Treatment*Age | AK | Predator | ASY |  | 0 | . | . | . |
| Population*Treatment*Age | AK | Predator | SY |  | 0 | . | . | . |
| Population*Treatment*Age | NY | Control | ASY |  | 0.08955 | 0.7595 | -1.4071 | 1.5862 |
| Population*Treatment*Age | NY | Control | SY |  | 0 | . | . | . |
| Population*Treatment*Age | NY | Low_Tape | ASY |  | -0.02598 | 0.8786 | -1.7587 | 1.7068 |
| Population*Treatment*Age | NY | Low_Tape | SY |  | 0 | . | . | . |
| Population*Treatment*Age | NY | Predator | ASY |  | 0 | . | . | . |
| Population*Treatment*Age | NY | Predator | SY |  | 0 | . | . | . |
| Population*Treatment*Age | TN | Control | ASY |  | 0.8868 | 0.692 | -0.4753 | 2.2489 |
| Population*Treatment*Age | TN | Control | SY |  | 0 | . | . | . |
| Population*Treatment*Age | TN | Low_Tape | ASY |  | 0 | . | . | . |
| Population*Treatment*Age | TN | Low_Tape | SY |  | 0 | . | . | . |
| Population*Treatment*Age | TN | Predator | ASY |  | 0 | . | . | . |
| Population*Treatment*Age | TN | Predator | SY |  | 0 | . | . | . |
| Population*Treatment*Age | WY | Control | ASY |  | 0 | . | . | . |
| Population*Treatment*Age | WY | Control | SY |  | 0 | . | . | . |
| Population*Treatment*Age | WY | Predator | ASY |  | 0 | . | . | . |
| Population*Treatment*Age | WY | Predator | SY |  | 0 | . | . | . |
| Treatment*Age*LHSS | | Control | ASY | Mid Inc | 0.657 | 0.5148 | -0.3571 | 1.671 |
| Treatment*Age*LHSS | | Control | ASY | Nestling | 0 | . | . | . |
| Treatment*Age*LHSS | | Control | SY | Mid Inc | 0 | . | . | . |
| Treatment*Age*LHSS | | Control | SY | Nestling | 0 | . | . | . |
| Treatment*Age*LHSS | | Low_Tape | ASY | Mid Inc | 0.8544 | 0.5134 | -0.1575 | 1.8662 |
| Treatment*Age*LHSS | | Low_Tape | ASY | Nestling | 0 | . | . | . |
| Treatment*Age*LHSS | | Low_Tape | SY | Mid Inc | 0 | . | . | . |
| Treatment*Age*LHSS | | Low_Tape | SY | Nestling | 0 | . | . | . |
| Treatment*Age*LHSS | | Predator | ASY | Mid Inc | 0 | . | . | . |
| Treatment*Age*LHSS | | Predator | ASY | Nestling | 0 | . | . | . |
| Treatment*Age*LHSS | | Predator | SY | Mid Inc | 0 | . | . | . |
| Treatment*Age*LHSS | | Predator | SY | Nestling | 0 | . | . | . |
| Population*Treatment*Age*LHSS | AK | Control | ASY | Mid Inc | -0.4405 | 0.4596 | -1.3462 | 0.4651 |
| Population*Treatment*Age*LHSS | AK | Control | ASY | Nestling | 0 | . | . | . |
| Population*Treatment*Age*LHSS | AK | Control | SY | Mid Inc | -0.2507 | 0.5217 | -1.2786 | 0.7772 |
| Population*Treatment*Age*LHSS | AK | Control | SY | Nestling | 0 | . | . | . |
| Population*Treatment*Age*LHSS | AK | Low_Tape | ASY | Mid Inc | 0.184 | 0.4694 | -0.7414 | 1.1094 |
| Population*Treatment*Age*LHSS | AK | Low_Tape | ASY | Nestling | 0 | . | . | . |
| Population*Treatment*Age*LHSS | AK | Low_Tape | SY | Mid Inc | 1.0383 | 0.5442 | -0.03383 | 2.1103 |
| Population*Treatment*Age*LHSS | AK | Low_Tape | SY | Nestling | 0 | . | . | . |
| Population*Treatment*Age*LHSS | AK | Predator | ASY | Mid Inc | 0 | . | . | . |
| Population*Treatment*Age*LHSS | AK | Predator | ASY | Nestling | 0 | . | . | . |
| Population*Treatment*Age*LHSS | AK | Predator | SY | Mid Inc | 0 | . | . | . |
| Population*Treatment*Age*LHSS | AK | Predator | SY | Nestling | 0 | . | . | . |
| Population*Treatment*Age*LHSS | NY | Control | ASY | Mid Inc | -0.5201 | 0.5734 | -1.65 | 0.6099 |
| Population*Treatment*Age*LHSS | NY | Control | ASY | Nestling | 0 | . | . | . |
| Population*Treatment*Age*LHSS | NY | Control | SY | Mid Inc | -0.1245 | 0.6049 | -1.3162 | 1.0673 |
| Population*Treatment*Age*LHSS | NY | Control | SY | Nestling | 0 | . | . | . |
| Population*Treatment*Age*LHSS | NY | Low_Tape | ASY | Mid Inc | -0.4431 | 0.5623 | -1.5514 | 0.6652 |
| Population*Treatment*Age*LHSS | NY | Low_Tape | ASY | Nestling | 0 | . | . | . |
| Population*Treatment*Age*LHSS | NY | Low_Tape | SY | Mid Inc | -0.03251 | 0.7859 | -1.5812 | 1.5161 |
| Population*Treatment*Age*LHSS | NY | Low_Tape | SY | Nestling | 0 | . | . | . |
| Population*Treatment*Age*LHSS | NY | Predator | ASY | Mid Inc | 0 | . | . | . |
| Population*Treatment*Age*LHSS | NY | Predator | ASY | Nestling | 0 | . | . | . |
| Population*Treatment*Age*LHSS | NY | Predator | SY | Mid Inc | 0 | . | . | . |
| Population*Treatment*Age*LHSS | NY | Predator | SY | Nestling | 0 | . | . | . |
| Population*Treatment*Age*LHSS | TN | Control | ASY | Mid Inc | -0.07567 | 0.456 | -0.9742 | 0.8229 |
| Population*Treatment*Age*LHSS | TN | Control | ASY | Nestling | 0 | . | . | . |
| Population*Treatment*Age*LHSS | TN | Control | SY | Mid Inc | -0.03193 | 0.5344 | -1.0847 | 1.0208 |
| Population*Treatment*Age*LHSS | TN | Control | SY | Nestling | 0 | . | . | . |
| Population*Treatment*Age*LHSS | TN | Low_Tape | ASY | Mid Inc | 0 | . | . | . |
| Population*Treatment*Age*LHSS | TN | Low_Tape | ASY | Nestling | 0 | . | . | . |
| Population*Treatment*Age*LHSS | TN | Low_Tape | SY | Mid Inc | 0 | . | . | . |
| Population*Treatment*Age*LHSS | TN | Low_Tape | SY | Nestling | 0 | . | . | . |
| Population*Treatment*Age*LHSS | TN | Predator | ASY | Mid Inc | 0 | . | . | . |
| Population*Treatment*Age*LHSS | TN | Predator | ASY | Nestling | 0 | . | . | . |
| Population*Treatment*Age*LHSS | TN | Predator | SY | Mid Inc | 0 | . | . | . |
| Population*Treatment*Age*LHSS | TN | Predator | SY | Nestling | 0 | . | . | . |
| Population*Treatment*Age*LHSS | WY | Control | ASY | Mid Inc | 0 | . | . | . |
| Population*Treatment*Age*LHSS | WY | Control | ASY | Nestling | 0 | . | . | . |
| Population*Treatment*Age*LHSS | WY | Control | SY | Mid Inc | 0 | . | . | . |
| Population*Treatment*Age*LHSS | WY | Control | SY | Nestling | 0 | . | . | . |
| Population*Treatment*Age*LHSS | WY | Predator | ASY | Mid Inc | 0 | . | . | . |
| Population*Treatment*Age*LHSS | WY | Predator | ASY | Nestling | 0 | . | . | . |
| Population*Treatment*Age*LHSS | WY | Predator | SY | Mid Inc | 0 | . | . | . |
| Population*Treatment*Age*LHSS | WY | Predator | SY | Nestling | 0 | . | . | . |

Table S18: Parameter estimates, standard error and confidence interval for the generalized linear mixed model for females stress response with total average temperature unpredictability as continuous predictor. The model was fitted with a gamma law and individual and experimental year specified as random factor. Unpred = average temperature unpredictability, Spl = Sample, Base = Baseline, Str = stress-induced, dex = post-dex, LHSS = Life history substage, Mid Inc = Mid Incubation, End incubation = End incubation, Nestling = Nestling provisioning, Rel_CI = relative clutch initiation date, Mass = Body mass

| Effect | Treatment | Age | LHSS | Estimate | SE | Lower | Upper |
| --- | --- | --- | --- | --- | --- | --- | --- |
|  |  |  |  |  |  |  |  |
| Intercept |  |  |  | 5.5352 | 0.5995 | 4.3561 | 6.7143 |
| Age |  | ASY |  | -0.2813 | 0.5304 | -1.326 | 0.7634 |
| Age |  | SY |  | 0 | . | . | . |
| LHSS |  |  | Mid Inc | 0.5107 |  | -0.1633 | 1.1846 |
| LHSS |  |  | Nestling | 0 | . | . | . |
| Treatment | Control |  |  | -0.6285 | 0.4777 | -1.5715 | 0.3144 |
| Treatment | Low_Tape |  |  | -0.4419 | 0.5093 | -1.4453 | 0.5615 |
| Treatment | Predator |  |  | 0 | . | . | . |
| Rel_CI |  |  |  | -0.01303 | 0.006235 | -0.02531 | -0.00075 |
| Mass |  |  |  | -0.1407 | 0.02325 | -0.1864 | -0.09497 |
| unpred |  |  |  | 1.4914 | 0.4276 | 0.6493 | 2.3336 |
| unpred*Age | | ASY |  | 0.464 | 1.5069 | -2.4988 | 3.4267 |
| unpred*Age | | SY |  | 0 | . | . | . |
| unpred*LHSS | |  | Mid Inc | -0.4841 | 1.2446 | -2.9355 | 1.9673 |
| unpred*LHSS | |  | Nestling | 0 | . | . | . |
| unpred*Treatment | Control |  |  | 2.0343 | 1.524 | -0.962 | 5.0305 |
| unpred*Treatment | Low_Tape |  |  | -0.8001 | 2.0118 | -4.7562 | 3.156 |
| unpred*Treatment | Predator |  |  | 0 | . | . | . |
| Age*LHSS | | ASY | Mid Inc | -0.1732 | 0.6517 | -1.4573 | 1.1109 |
| Age*LHSS | | ASY | Nestling | 0 | . | . | . |
| Age*LHSS | | SY | Mid Inc | 0 | . | . | . |
| Age*LHSS | | SY | Nestling | 0 | . | . | . |
| Treatment*Age | Control | ASY |  | 1.066 | 0.8136 | -0.5336 | 2.6657 |
| Treatment*Age | Control | SY |  | 0 | . | . | . |
| Treatment*Age | Low_Tape | ASY |  | -0.1893 | 0.9363 | -2.0301 | 1.6516 |
| Treatment*Age | Low_Tape | SY |  | 0 | . | . | . |
| Treatment*Age | Predator | ASY |  | 0 | . | . | . |
| Treatment*Age | Predator | SY |  | 0 | . | . | . |
| Treatment*LHSS | Control |  | Mid Inc | 0.06348 | 0.6514 | -1.2202 | 1.3472 |
| Treatment*LHSS | Control |  | Nestling | 0 | . | . | . |
| Treatment*LHSS | Low_Tape |  | Mid Inc | -0.8205 | 0.758 | -2.3135 | 0.6724 |
| Treatment*LHSS | Low_Tape |  | Nestling | 0 | . | . | . |
| Treatment*LHSS | Predator |  | Mid Inc | 0 | . | . | . |
| Treatment*LHSS | Predator |  | Nestling | 0 | . | . | . |
| unpred*Treatment*LHSS | Control |  | Mid Inc | -0.5642 | 1.6644 | -3.8441 | 2.7156 |
| unpred*Treatment*LHSS | Control |  | Nestling | 0 | . | . | . |
| unpred*Treatment*LHSS | Low_Tape |  | Mid Inc | 2.4595 | 2.1308 | -1.7373 | 6.6563 |
| unpred*Treatment*LHSS | Low_Tape |  | Nestling | 0 | . | . | . |
| unpred*Treatment*LHSS | Predator |  | Mid Inc | 0 | . | . | . |
| unpred*Treatment*LHSS | Predator |  | Nestling | 0 | . | . | . |
| unpred*Age*LHSS | | ASY | Mid Inc | 0.3966 | 1.6357 | -2.8263 | 3.6195 |
| unpred*Age*LHSS | | ASY | Nestling | 0 | . | . | . |
| unpred*Age*LHSS | | SY | Mid Inc | 0 | . | . | . |
| unpred*Age*LHSS | | SY | Nestling | 0 | . | . | . |
| unpred*Treatment*Age | Control | ASY |  | -3.1894 | 2.0689 | -7.2571 | 0.8783 |
| unpred*Treatment*Age | Control | SY |  | 0 | . | . | . |
| unpred*Treatment*Age | Low_Tape | ASY |  | 0.4303 | 2.5959 | -4.6738 | 5.5345 |
| unpred*Treatment*Age | Low_Tape | SY |  | 0 | . | . | . |
| unpred*Treatment*Age | Predator | ASY |  | 0 | . | . | . |
| unpred*Treatment*Age | Predator | SY |  | 0 | . | . | . |
| Treatment*Age*LHSS | Control | ASY | Mid Inc | 0.2855 | 0.8934 | -1.4756 | 2.0467 |
| Treatment*Age*LHSS | Control | ASY | Nestling | 0 | . | . | . |
| Treatment*Age*LHSS | Control | SY | Mid Inc | 0 | . | . | . |
| Treatment*Age*LHSS | Control | SY | Nestling | 0 | . | . | . |
| Treatment*Age*LHSS | Low_Tape | ASY | Mid Inc | 1.4519 | 1.0099 | -0.5383 | 3.442 |
| Treatment*Age*LHSS | Low_Tape | ASY | Nestling | 0 | . | . | . |
| Treatment*Age*LHSS | Low_Tape | SY | Mid Inc | 0 | . | . | . |
| Treatment*Age*LHSS | Low_Tape | SY | Nestling | 0 | . | . | . |
| Treatment*Age*LHSS | Predator | ASY | Mid Inc | 0 | . | . | . |
| Treatment*Age*LHSS | Predator | ASY | Nestling | 0 | . | . | . |
| Treatment*Age*LHSS | Predator | SY | Mid Inc | 0 | . | . | . |
| Treatment*Age*LHSS | Predator | SY | Nestling | 0 | . | . | . |
| unpred*Treatment*Age*LHSS | Control | ASY | Mid Inc | 0.6265 | 2.259 | -3.826 | 5.079 |
| unpred*Treatment*Age*LHSS | Control | ASY | Nestling | 0 | . | . | . |
| unpred*Treatment*Age*LHSS | Control | SY | Mid Inc | 0 | . | . | . |
| unpred*Treatment*Age*LHSS | Control | SY | Nestling | 0 | . | . | . |
| unpred*Treatment*Age*LHSS | Low_Tape | ASY | Mid Inc | -3.2287 | 2.782 | -8.7103 | 2.2529 |
| unpred*Treatment*Age*LHSS | Low_Tape | ASY | Nestling | 0 | . | . | . |
| unpred*Treatment*Age*LHSS | Low_Tape | SY | Mid Inc | 0 | . | . | . |
| unpred*Treatment*Age*LHSS | Low_Tape | SY | Nestling | 0 | . | . | . |
| unpred*Treatment*Age*LHSS | Predator | ASY | Mid Inc | 0 | . | . | . |
| unpred*Treatment*Age*LHSS | Predator | ASY | Nestling | 0 | . | . | . |
| unpred*Treatment*Ag*LHSS | Predator | SY | Mid Inc | 0 | . | . | . |
| unpred*Treatment*Age*LHSS | Predator | SY | Nestling | 0 | . | . | . |

Table S19: Parameter estimates, standard error and confidence interval for the generalized linear mixed model for females stress response with total breeding season length as continuous predictor. The model was fitted with a gamma law and individual and experimental year specified as random factor. Breedinglength = Total breeding season length, Spl = Sample, Base = Baseline, Str = stress-induced, dex = post-dex, LHSS = Life history substage, Mid Inc = Mid Incubation, End incubation = End incubation, Nestling = Nestling provisioning, Rel_CI = relative clutch initiation date, Mass = Body mass

| Effect | Treatment | Age | LHSS | Estimate | SE | Lower | Upper |
| --- | --- | --- | --- | --- | --- | --- | --- |
|  |  |  |  |  |  |  |  |
| Intercept |  |  |  | 7.317 | 0.7306 | 5.8801 | 8.7538 |
| Age |  | ASY |  | 0.2414 | 0.8469 | -1.4257 | 1.9084 |
| Age |  | SY |  | 0 | . | . | . |
| LHSS |  |  | Mid Inc | -0.00078 | 0.4859 | -0.9584 | 0.9568 |
| LHSS |  |  | Nestling | 0 | . | . | . |
| Treatment | Control |  |  | 1.2257 | 0.7733 | -0.2975 | 2.749 |
| Treatment | Low_Tape |  |  | 0.5133 | 0.8375 | -1.1354 | 2.1621 |
| Treatment | Predator |  |  | 0 | . | . | . |
| Rel_CI |  |  |  | -0.01232 | 0.006237 | -0.02461 | -0.00004 |
| Mass |  |  |  | -0.1554 | 0.02146 | -0.1976 | -0.1132 |
| breedinglenght | |  |  | -0.01355 | 0.008037 | -0.02937 | -0.00226 |
| breedinglength*Age | | ASY |  | 0.000132 | 0.01253 | -0.02451 | 0.02478 |
| breedinglength*Age | | SY |  | 0 | . | . | . |
| breedinglength*LHSS | | | Mid Inc | 0.01046 | 0.01031 | -0.00986 | 0.03078 |
| breedinglength*LHSS | | | Nestling | 0 | . | . | . |
| breedinglength*Treatment | Control |  |  | -0.01573 | 0.01256 | -0.04043 | 0.008973 |
| breedinglength*Treatment | Low_Tape |  |  | 0.01149 | 0.01559 | -0.01917 | 0.04215 |
| breedinglength*Treatment | Predator |  |  | 0 | . | . | . |
| Age*LHSS | | ASY | Mid Inc | 0.4699 | 1.072 | -1.6428 | 2.5826 |
| Age*LHSS | | ASY | Nestling | 0 | . | . | . |
| Age*LHSS | | SY | Mid Inc | 0 | . | . | . |
| Age*LHSS | | SY | Nestling | 0 | . | . | . |
| Treatment*Age | Control | ASY |  | -1.7033 | 1.3733 | -4.4039 | 0.9973 |
| Treatment*Age | Control | SY |  | 0 | . | . | . |
| Treatment*Age | Low_Tape | ASY |  | 0.5306 | 1.6568 | -2.7271 | 3.7883 |
| Treatment*Age | Low_Tape | SY |  | 0 | . | . | . |
| Treatment*Age | Predator | ASY |  | 0 | . | . | . |
| Treatment*Age | Predator | SY |  | 0 | . | . | . |
| Treatment*LHSS | Control |  | Mid Inc | -0.246 | 1.0931 | -2.4004 | 1.9085 |
| Treatment*LHSS | Control |  | Nestling | 0 | . | . | . |
| Treatment*LHSS | Low_Tape |  | Mid Inc | 1.9786 | 1.3727 | -0.7252 | 4.6824 |
| Treatment*LHSS | Low_Tape |  | Nestling | 0 | . | . | . |
| Treatment*LHSS | Predator |  | Mid Inc | 0 | . | . | . |
| Treatment*LHSS | Predator |  | Nestling | 0 | . | . | . |
| breedinglength*Treatment*LHSS | Control |  | Mid Inc | 0.001062 | 0.01386 | -0.02626 | 0.02839 |
| breedinglength*Treatment*LHSS | Control |  | Nestling | 0 | . | . | . |
| breedinglength*Treatment*LHSS | Low_Tape |  | Mid Inc | -0.02482 | 0.01658 | -0.05748 | 0.007851 |
| breedinglength*Treatment*LHSS | Low_Tape |  | Nestling | 0 | . | . | . |
| breedinglength*Treatment*LHSS | Predator |  | Mid Inc | 0 | . | . | . |
| breedinglength*Treatment*LHSS | Predator |  | Nestling | 0 | . | . | . |
| breedinglength*Age*LHSS | | ASY | Mid Inc | -0.00654 | 0.01373 | -0.0336 | 0.02053 |
| breedinglength*Age*LHSS | | ASY | Nestling | 0 | . | . | . |
| breedinglength*Age*LHSS | | SY | Mid Inc | 0 | . | . | . |
| breedinglength*Age*LHSS | | SY | Nestling | 0 | . | . | . |
| breedinglength*Treatment*Age | Control | ASY |  | 0.01993 | 0.01738 | -0.01426 | 0.05411 |
| breedinglength*Treatment*Age | Control | SY |  | 0 | . | . | . |
| breedinglength*Treatment*Age | Low_Tape | ASY |  | -0.0075 | 0.0202 | -0.04722 | 0.03221 |
| breedinglength*Treatment*Age | Low_Tape | SY |  | 0 | . | . | . |
| breedinglength*Treatment*Age | Predator | ASY |  | 0 | . | . | . |
| breedinglength*Treatment*Age | Predator | SY |  | 0 | . | . | . |
| Treatment*Age*LHSS | Control | ASY | Mid Inc | 0.2447 | 1.507 | -2.726 | 3.2153 |
| Treatment*Age*LHSS | Control | ASY | Nestling | 0 | . | . | . |
| Treatment*Age*LHSS | Control | SY | Mid Inc | 0 | . | . | . |
| Treatment*Age*LHSS | Control | SY | Nestling | 0 | . | . | . |
| Treatment*Age*LHSS | Low_Tape | ASY | Mid Inc | -1.9665 | 1.7733 | -5.4608 | 1.5278 |
| Treatment*Age*LHSS | Low_Tape | ASY | Nestling | 0 | . | . | . |
| Treatment*Age*LHSS | Low_Tape | SY | Mid Inc | 0 | . | . | . |
| Treatment*Age*LHSS | Low_Tape | SY | Nestling | 0 | . | . | . |
| Treatment*Age*LHSS | Predator | ASY | Mid Inc | 0 | . | . | . |
| Treatment*Age*LHSS | Predator | ASY | Nestling | 0 | . | . | . |
| Treatment*Age*LHSS | Predator | SY | Mid Inc | 0 | . | . | . |
| Treatment*Age*LHSS | Predator | SY | Nestling | 0 | . | . | . |
| breedinglength*Treatment*Age*LHSS | Control | ASY | Mid Inc | 0.00372 | 0.01914 | -0.03401 | 0.04145 |
| breedinglength*Treatment*Age*LHSS | Control | ASY | Nestling | 0 | . | . | . |
| breedinglength*Treatment*Age*LHSS | Control | SY | Mid Inc | 0 | . | . | . |
| breedinglength*Treatment*Age*LHSS | Control | SY | Nestling | 0 | . | . | . |
| breedinglength*Treatment*Age*LHSS | Low_Tape | ASY | Mid Inc | 0.02917 | 0.02171 | -0.01362 | 0.07196 |
| breedinglength*Treatment*Age*LHSS | Low_Tape | ASY | Nestling | 0 | . | . | . |
| breedinglength*Treatment*Age*LHSS | Low_Tape | SY | Mid Inc | 0 | . | . | . |
| breedinglength*Treatment*Age*LHSS | Low_Tape | SY | Nestling | 0 | . | . | . |
| breedinglength*Treatment*Age*LHSS | Predator | ASY | Mid Inc | 0 | . | . | . |
| breedinglength*Treatment*Age*LHSS | Predator | ASY | Nestling | 0 | . | . | . |
| breedinglength*Treatment*Age*LHSS | Predator | SY | Mid Inc | 0 | . | . | . |
| breedinglength*Treatment*Age*LHSS | Predator | SY | Nestling | 0 | . | . | . |

Table S20: Parameter estimates, standard error and confidence interval for the generalized linear mixed model for females negative feedback. The model was fitted with a normal law and individual and experimental year specified as random factor and populations as random slope. Spl = Sample, Base = Baseline, Str = stress-induced, dex = post-dex, LHSS = Life history substage, Mid Inc = Mid Incubation, End incubation = End incubation, Nestling = Nestling provisioning, Rel_CI = relative clutch initiation date, Mass = Body mass

| Effect | Location | Treatment | Age | LHSS | Estimate | SE | Lower | Upper |
| --- | --- | --- | --- | --- | --- | --- | --- | --- |
|  |  |  |  |  |  |  |  |  |
| Intercept |  |  |  |  | -75.1235 | 18.5318 | -111.57 | -38.6769 |
| Location | AK |  |  |  | -15.6154 | 12.9498 | -41.1118 | 9.8811 |
| Location | NY |  |  |  | 5.9821 | 13.7534 | -21.0982 | 33.0623 |
| Location | TN |  |  |  | 8.3428 | 13.0909 | -17.4375 | 34.1231 |
| Location | WY |  |  |  | 0 | . | . | . |
| Age |  |  | ASY |  | -4.9779 | 15.4965 | -35.5377 | 25.5819 |
| Age |  |  | SY |  | 0 | . | . | . |
| LHSS |  |  |  | Mid Inc | -22.9016 | 9.4665 | -41.5392 | -4.2641 |
| LHSS |  |  |  | Nestling | 0 | . | . | . |
| Treatment |  | Control |  |  | 12.7945 | 17.0765 | -20.8892 | 46.4782 |
| Treatment |  | Low_Tape |  |  | -1.9201 | 9.449 | -20.5342 | 16.694 |
| Treatment |  | Predator |  |  | 0 | . | . | . |
| Rel_CI |  |  |  |  | 0.4157 | 0.179 | 0.06351 | 0.768 |
| Mass |  |  |  |  | 2.7318 | 0.7856 | 1.1864 | 4.2773 |
| Location*Age | AK |  | ASY |  | 12.7589 | 17.7046 | -22.0987 | 47.6164 |
| Location*Age | AK |  | SY |  | 0 | . | . | . |
| Location*Age | NY |  | ASY |  | 3.4871 | 19.3401 | -34.595 | 41.5692 |
| Location*Age | NY |  | SY |  | 0 | . | . | . |
| Location*Age | TN |  | ASY |  | 3.3674 | 17.7115 | -31.513 | 38.2479 |
| Location*Age | TN |  | SY |  | 0 | . | . | . |
| Location*Age | WY |  | ASY |  | 0 | . | . | . |
| Location*Age | WY |  | SY |  | 0 | . | . | . |
| Location*LHSS | AK |  |  | Mid Inc | 30.3608 | 11.8771 | 6.9736 | 53.748 |
| Location*LHSS | AK |  |  | Nestling | 0 | . | . | . |
| Location*LHSS | NY |  |  | Mid Inc | 14.7742 | 13.862 | -12.5186 | 42.067 |
| Location*LHSS | NY |  |  | Nestling | 0 | . | . | . |
| Location*LHSS | TN |  |  | Mid Inc | 5.978 | 12.6009 | -18.8362 | 30.7922 |
| Location*LHSS | TN |  |  | Nestling | 0 | . | . | . |
| Location*LHSS | WY |  |  | Mid Inc | 0 | . | . | . |
| Location*LHSS | WY |  |  | Nestling | 0 | . | . | . |
| Location*Treatment | AK | Control |  |  | -16.5495 | 19.4549 | -54.8581 | 21.759 |
| Location*Treatment | AK | Low_Tape |  |  | 11.2253 | 15.0012 | -18.3048 | 40.7554 |
| Location*Treatment | AK | Predator |  |  | 0 | . | . | . |
| Location*Treatment | NY | Control |  |  | -20.8936 | 20.8 | -61.8505 | 20.0632 |
| Location*Treatment | NY | Low_Tape |  |  | -9.971 | 21.2001 | -51.8528 | 31.9109 |
| Location*Treatment | NY | Predator |  |  | 0 | . | . | . |
| Location*Treatment | TN | Control |  |  | -9.2133 | 19.0352 | -46.7121 | 28.2854 |
| Location*Treatment | TN | Low_Tape |  |  | 0 | . | . | . |
| Location*Treatment | TN | Predator |  |  | 0 | . | . | . |
| Location*Treatment | WY | Control |  |  | 0 | . | . | . |
| Location*Treatment | WY | Predator |  |  | 0 | . | . | . |
| Age*LHSS | |  | ASY | Mid Inc | 5.0783 | 12.8794 | -20.2791 | 30.4356 |
| Age*LHSS | |  | ASY | Nestling | 0 | . | . | . |
| Age*LHSS | |  | SY | Mid Inc | 0 | . | . | . |
| Age*LHSS | |  | SY | Nestling | 0 | . | . | . |
| Treatment*Age | | Control | ASY |  | 7.5187 | 21.9437 | -35.7875 | 50.8248 |
| Treatment*Age | | Control | SY |  | 0 | . | . | . |
| Treatment*Age | | Low_Tape | ASY |  | 6.773 | 12.602 | -18.0514 | 31.598 |
| Treatment*Age | | Low_Tape | SY |  | 0 | . | . | . |
| Treatment*Age | | Predator | ASY |  | 0 | . | . | . |
| Treatment*Age | | Predator | SY |  | 0 | . | . | . |
| Treatment*LHSS | | Control |  | Mid Inc | -13.4844 | 14.0634 | -41.1734 | 14.2046 |
| Treatment*LHSS | | Control |  | Nestling | 0 | . | . | . |
| Treatment*LHSS | | Low_Tape |  | Mid Inc | 12.207 | 11.5254 | -10.4974 | 34.9114 |
| Treatment*LHSS | | Low_Tape |  | Nestling | 0 | . | . | . |
| Treatment*LHSS | | Predator |  | Mid Inc | 0 | . | . | . |
| Treatment*LHSS | | Predator |  | Nestling | 0 | . | . | . |
| Location*Age*LHSS | AK |  | ASY | Mid Inc | -17.3253 | 16.5156 | -49.8461 | 15.1956 |
| Location*Age*LHSS | AK |  | ASY | Nestling | 0 | . | . | . |
| Location*Age*LHSS | AK |  | SY | Mid Inc | 0 | . | . | . |
| Location*Age*LHSS | AK |  | SY | Nestling | 0 | . | . | . |
| Location*Age*LHSS | NY |  | ASY | Mid Inc | -1.5358 | 19.6818 | -40.2859 | 37.2144 |
| Location*Age*LHSS | NY |  | ASY | Nestling | 0 | . | . | . |
| Location*Age*LHSS | NY |  | SY | Mid Inc | 0 | . | . | . |
| Location*Age*LHSS | NY |  | SY | Nestling | 0 | . | . | . |
| Location*Age*LHSS | TN |  | ASY | Mid Inc | 3.6216 | 16.9454 | -29.7481 | 36.9913 |
| Location*Age*LHSS | TN |  | ASY | Nestling | 0 | . | . | . |
| Location*Age*LHSS | TN |  | SY | Mid Inc | 0 | . | . | . |
| Location*Age*LHSS | TN |  | SY | Nestling | 0 | . | . | . |
| Location*Age*LHSS | WY |  | ASY | Mid Inc | 0 | . | . | . |
| Location*Age*LHSS | WY |  | ASY | Nestling | 0 | . | . | . |
| Location*Age*LHSS | WY |  | SY | Mid Inc | 0 | . | . | . |
| Location*Age*LHSS | WY |  | SY | Nestling | 0 | . | . | . |
| Location*Treatment*Age | AK | Control | ASY |  | -4.8956 | 25.4654 | -55.0445 | 45.2533 |
| Location*Treatment*Age | AK | Control | SY |  | 0 | . | . | . |
| Location*Treatment*Age | AK | Low_Tape | ASY |  | -3.0129 | 19.6879 | -41.7697 | 35.7439 |
| Location*Treatment*Age | AK | Low_Tape | SY |  | 0 | . | . | . |
| Location*Treatment*Age | AK | Predator | ASY |  | 0 | . | . | . |
| Location*Treatment*Age | AK | Predator | SY |  | 0 | . | . | . |
| Location*Treatment*Age | NY | Control | ASY |  | 0.1426 | 27.771 | -54.5503 | 54.8355 |
| Location*Treatment*Age | NY | Control | SY |  | 0 | . | . | . |
| Location*Treatment*Age | NY | Low_Tape | ASY |  | 7.8475 | 25.3857 | -42.2762 | 57.9712 |
| Location*Treatment*Age | NY | Low_Tape | SY |  | 0 | . | . | . |
| Location*Treatment*Age | NY | Predator | ASY |  | 0 | . | . | . |
| Location*Treatment*Age | NY | Predator | SY |  | 0 | . | . | . |
| Location*Treatment*Age | TN | Control | ASY |  | -11.7828 | 24.8563 | -60.7547 | 37.1891 |
| Location*Treatment*Age | TN | Control | SY |  | 0 | . | . | . |
| Location*Treatment*Age | TN | Low_Tape | ASY |  | 0 | . | . | . |
| Location*Treatment*Age | TN | Low_Tape | SY |  | 0 | . | . | . |
| Location*Treatment*Age | TN | Predator | ASY |  | 0 | . | . | . |
| Location*Treatment*Age | TN | Predator | SY |  | 0 | . | . | . |
| Location*Treatment*Age | WY | Control | ASY |  | 0 | . | . | . |
| Location*Treatment*Age | WY | Control | SY |  | 0 | . | . | . |
| Location*Treatment*Age | WY | Predator | ASY |  | 0 | . | . | . |
| Location*Treatment*Age | WY | Predator | SY |  | 0 | . | . | . |
| Treatment*Age*LHSS | | Control | ASY | Mid Inc | -15.4399 | 17.8118 | -50.5098 | 19.6299 |
| Treatment*Age*LHSS | | Control | ASY | Nestling | 0 | . | . | . |
| Treatment*Age*LHSS | | Control | SY | Mid Inc | 0 | . | . | . |
| Treatment*Age*LHSS | | Control | SY | Nestling | 0 | . | . | . |
| Treatment*Age*LHSS | | Low_Tape | ASY | Mid Inc | -25.6004 | 15.8984 | -56.919 | 5.7182 |
| Treatment*Age*LHSS | | Low_Tape | ASY | Nestling | 0 | . | . | . |
| Treatment*Age*LHSS | | Low_Tape | SY | Mid Inc | 0 | . | . | . |
| Treatment*Age*LHSS | | Low_Tape | SY | Nestling | 0 | . | . | . |
| Treatment*Age*LHSS | | Predator | ASY | Mid Inc | 0 | . | . | . |
| Treatment*Age*LHSS | | Predator | ASY | Nestling | 0 | . | . | . |
| Treatment*Age*LHSS | | Predator | SY | Mid Inc | 0 | . | . | . |
| Treatment*Age*LHSS | | Predator | SY | Nestling | 0 | . | . | . |
| Location*Treatment*Age*LHSS | AK | Control | ASY | Mid Inc | 31.885 | 15.4578 | 1.4468 | 62.3233 |
| Location*Treatment*Age*LHSS | AK | Control | ASY | Nestling | 0 | . | . | . |
| Location*Treatment*Age*LHSS | AK | Control | SY | Mid Inc | 30.2737 | 17.7385 | -4.6523 | 65.1997 |
| Location*Treatment*Age*LHSS | AK | Control | SY | Nestling | 0 | . | . | . |
| Location*Treatment*Age*LHSS | AK | Low_Tape | ASY | Mid Inc | 1.5966 | 15.456 | -28.8453 | 32.0385 |
| Location*Treatment*Age*LHSS | AK | Low_Tape | ASY | Nestling | 0 | . | . | . |
| Location*Treatment*Age*LHSS | AK | Low_Tape | SY | Mid Inc | -27.1955 | 17.2493 | -61.1615 | 6.7704 |
| Location*Treatment*Age*LHSS | AK | Low_Tape | SY | Nestling | 0 | . | . | . |
| Location*Treatment*Age*LHSS | AK | Predator | ASY | Mid Inc | 0 | . | . | . |
| Location*Treatment*Age*LHSS | AK | Predator | ASY | Nestling | 0 | . | . | . |
| Location*Treatment*Age*LHSS | AK | Predator | SY | Mid Inc | 0 | . | . | . |
| Location*Treatment*Age*LHSS | AK | Predator | SY | Nestling | 0 | . | . | . |
| Location*Treatment*Age*LHSS | NY | Control | ASY | Mid Inc | 26.8661 | 18.8991 | -10.3442 | 64.0765 |
| Location*Treatment*Age*LHSS | NY | Control | ASY | Nestling | 0 | . | . | . |
| Location*Treatment*Age*LHSS | NY | Control | SY | Mid Inc | 11.0536 | 20.2234 | -28.761 | 50.8683 |
| Location*Treatment*Age*LHSS | NY | Control | SY | Nestling | 0 | . | . | . |
| Location*Treatment*Age*LHSS | NY | Low_Tape | ASY | Mid Inc | 2.454 | 18.1317 | -33.2512 | 38.1593 |
| Location*Treatment*Age*LHSS | NY | Low_Tape | ASY | Nestling | 0 | . | . | . |
| Location*Treatment*Age*LHSS | NY | Low_Tape | SY | Mid Inc | -9.3992 | 24.564 | -57.7859 | 38.9875 |
| Location*Treatment*Age*LHSS | NY | Low_Tape | SY | Nestling | 0 | . | . | . |
| Location*Treatment*Age*LHSS | NY | Predator | ASY | Mid Inc | 0 | . | . | . |
| Location*Treatment*Age*LHSS | NY | Predator | ASY | Nestling | 0 | . | . | . |
| Location*Treatment*Age*LHSS | NY | Predator | SY | Mid Inc | 0 | . | . | . |
| Location*Treatment*Age*LHSS | NY | Predator | SY | Nestling | 0 | . | . | . |
| Location*Treatment*Age*LHSS | TN | Control | ASY | Mid Inc | 19.2512 | 15.296 | -10.8732 | 49.3755 |
| Location*Treatment*Age*LHSS | TN | Control | ASY | Nestling | 0 | . | . | . |
| Location*Treatment*Age*LHSS | TN | Control | SY | Mid Inc | 18.8556 | 17.8279 | -16.2516 | 53.9629 |
| Location*Treatment*Age*LHSS | TN | Control | SY | Nestling | 0 | . | . | . |
| Location*Treatment*Age*LHSS | TN | Low_Tape | ASY | Mid Inc | 0 | . | . | . |
| Location*Treatment*Age*LHSS | TN | Low_Tape | ASY | Nestling | 0 | . | . | . |
| Location*Treatment*Age*LHSS | TN | Low_Tape | SY | Mid Inc | 0 | . | . | . |
| Location*Treatment*Age*LHSS | TN | Low_Tape | SY | Nestling | 0 | . | . | . |
| Location*Treatment*Age*LHSS | TN | Predator | ASY | Mid Inc | 0 | . | . | . |
| Location*Treatment*Age*LHSS | TN | Predator | ASY | Nestling | 0 | . | . | . |
| Location*Treatment*Age*LHSS | TN | Predator | SY | Mid Inc | 0 | . | . | . |
| Location*Treatment*Age*LHSS | TN | Predator | SY | Nestling | 0 | . | . | . |
| Location*Treatment*Age*LHSS | WY | Control | ASY | Mid Inc | 0 | . | . | . |
| Location*Treatment*Age*LHSS | WY | Control | ASY | Nestling | 0 | . | . | . |
| Location*Treatment*Age*LHSS | WY | Control | SY | Mid Inc | 0 | . | . | . |
| Location*Treatment*Age*LHSS | WY | Control | SY | Nestling | 0 | . | . | . |
| Location*Treatment*Age*LHSS | WY | Predator | ASY | Mid Inc | 0 | . | . | . |
| Location*Treatment*Age*LHSS | WY | Predator | ASY | Nestling | 0 | . | . | . |
| Location*Treatment*Age*LHSS | WY | Predator | SY | Mid Inc | 0 | . | . | . |
| Location*Treatment*Age*LHSS | WY | Predator | SY | Nestling | 0 | . | . | . |

Table S21: Parameter estimates, standard error and confidence interval for the generalized linear mixed model for females negative feedback with total average temperature unpredictability as continuous predictor. The model was fitted with a normal law and individual and experimental year specified as random factor. Unpred = Average temperature unpredictability, Spl = Sample, Base = Baseline, Str = stress-induced, dex = post-dex, LHSS = Life history substage, Mid Inc = Mid Incubation, End incubation = End incubation, Nestling = Nestling provisioning, Rel_CI = relative clutch initiation date, Mass = Body mass

| Effect | Treatment | Age | LHSS | Estimate | SE | Lower | Upper |
| --- | --- | --- | --- | --- | --- | --- | --- |
|  |  |  |  |  |  |  |  |
| Intercept |  |  |  | -90.7251 | 21.6133 | -133.23 | -48.2182 |
| Age |  | ASY |  | -4.8955 | 19.996 | -44.2782 | 34.4873 |
| Age |  | SY |  | 0 | . | . | . |
| LHSS |  |  | Mid Inc | -24.1735 | 12.7442 | -49.2675 | 0.9204 |
| LHSS |  |  | Nestling | 0 | . | . | . |
| Treatment | Control |  |  | 4.2515 | 17.9072 | -31.0901 | 39.5931 |
| Treatment | Low_Tape |  |  | 3.3753 | 18.8011 | -33.6728 | 40.4233 |
| Treatment | Predator |  |  | 0 | . | . | . |
| Rel_CI |  |  |  | 0.5714 | 0.2195 | 0.1391 | 1.0036 |
| Mass |  |  |  | 4.4199 | 0.8226 | 2.8021 | 6.0377 |
| unpred |  |  |  | -31.4502 | 15.0511 | -61.0989 | -1.8015 |
| Str |  |  |  | -0.9143 | 0.01811 | -0.9499 | -0.8787 |
| unpred*Age |  | ASY |  | 24.3006 | 46.3324 | -66.859 | 115.46 |
| unpred*Age |  | SY |  | 0 | . | . | . |
| unpred*LHSS |  |  | Mid Inc | 42.7023 | 40.432 | -36.9318 | 122.34 |
| unpred*LHSS |  |  | Nestling | 0 | . | . | . |
| unpred*Treatment | Control |  |  | -18.1767 | 46.8206 | -110.29 | 73.9358 |
| unpred*Treatment | Low_Tape |  |  | 20.2822 | 60.5398 | -98.8314 | 139.4 |
| unpred*Treatment | Predator |  |  | 0 | . | . | . |
| Age*LHSS |  | ASY | Mid Inc | 19.2013 | 21.508 | -23.1671 | 61.5698 |
| Age*LHSS |  | ASY | Nestling | 0 | . | . | . |
| Age*LHSS |  | SY | Mid Inc | 0 | . | . | . |
| Age*LHSS |  | SY | Nestling | 0 | . | . | . |
| Treatment*Age | Control | ASY |  | -18.6948 | 23.5152 | -64.9947 | 27.6052 |
| Treatment*Age | Control | SY |  | 0 | . | . | . |
| Treatment*Age | Low_Tape | ASY |  | 9.7053 | 26.8511 | -43.1585 | 62.5691 |
| Treatment*Age | Low_Tape | SY |  | 0 | . | . | . |
| Treatment*Age | Predator | ASY |  | 0 | . | . | . |
| Treatment*Age | Predator | SY |  | 0 | . | . | . |
| Treatment*LHSS | Control |  | Mid Inc | 5.8812 | 21.5946 | -36.6574 | 48.4198 |
| Treatment*LHSS | Control |  | Nestling | 0 | . | . | . |
| Treatment*LHSS | Low_Tape |  | Mid Inc | 22.0335 | 24.0939 | -25.4227 | 69.4897 |
| Treatment*LHSS | Low_Tape |  | Nestling | 0 | . | . | . |
| Treatment*LHSS | Predator |  | Mid Inc | 0 | . | . | . |
| Treatment*LHSS | Predator |  | Nestling | 0 | . | . | . |
| unpred*Age*LHSS |  | ASY | Mid Inc | -52.8671 | 54.8476 | -160.89 | 55.1577 |
| unpred*Age*LHSS |  | ASY | Nestling | 0 | . | . | . |
| unpred*Age*LHSS |  | SY | Mid Inc | 0 | . | . | . |
| unpred*Age*LHSS |  | SY | Nestling | 0 | . | . | . |
| unpred*Treatment*Age | Control | ASY |  | 70.3323 | 63.8375 | -55.2545 | 195.92 |
| unpred*Treatment*Age | Control | SY |  | 0 | . | . | . |
| unpred*Treatment*Age | Low_Tape | ASY |  | -8.9817 | 78.3136 | -163.07 | 145.1 |
| unpred*Treatment*Age | Low_Tape | SY |  | 0 | . | . | . |
| unpred*Treatment*Age | Predator | ASY |  | 0 | . | . | . |
| unpred*Treatment*Age | Predator | SY |  | 0 | . | . | . |
| unpred*Treatment*LHSS | Control |  | Mid Inc | -5.6561 | 55.9849 | -115.92 | 104.61 |
| unpred*Treatment*LHSS | Control |  | Nestling | 0 | . | . | . |
| unpred*Treatment*LHSS | Low_Tape |  | Mid Inc | -56.2791 | 68.9683 | -192.07 | 79.5083 |
| unpred*Treatment*LHSS | Low_Tape |  | Nestling | 0 | . | . | . |
| unpred*Treatment*LHSS | Predator |  | Mid Inc | 0 | . | . | . |
| unpred*Treatment*LHSS | Predator |  | Nestling | 0 | . | . | . |
| Treatment*Age*LHSS | Control | ASY | Mid Inc | -2.7991 | 29.9395 | -61.7766 | 56.1785 |
| Treatment*Age*LHSS | Control | ASY | Nestling | 0 | . | . | . |
| Treatment*Age*LHSS | Control | SY | Mid Inc | 0 | . | . | . |
| Treatment*Age*LHSS | Control | SY | Nestling | 0 | . | . | . |
| Treatment*Age*LHSS | Low_Tape | ASY | Mid Inc | -45.0599 | 32.857 | -109.78 | 19.6591 |
| Treatment*Age*LHSS | Low_Tape | ASY | Nestling | 0 | . | . | . |
| Treatment*Age*LHSS | Low_Tape | SY | Mid Inc | 0 | . | . | . |
| Treatment*Age*LHSS | Low_Tape | SY | Nestling | 0 | . | . | . |
| Treatment*Age*LHSS | Predator | ASY | Mid Inc | 0 | . | . | . |
| Treatment*Age*LHSS | Predator | ASY | Nestling | 0 | . | . | . |
| Treatment*Age*LHSS | Predator | SY | Mid Inc | 0 | . | . | . |
| Treatment*Age*LHSS | Predator | SY | Nestling | 0 | . | . | . |
| unpred*Treatment*Age*LHSS | Control | ASY | Mid Inc | -35.9986 | 76.5844 | -186.84 | 114.84 |
| unpred*Treatment*Age*LHSS | Control | ASY | Nestling | 0 | . | . | . |
| unpred*Treatment*Age*LHSS | Control | SY | Mid Inc | 0 | . | . | . |
| unpred*Treatment*Age*LHSS | Control | SY | Nestling | 0 | . | . | . |
| unpred*Treatment*Age*LHSS | Low_Tape | ASY | Mid Inc | 96.5079 | 91.8008 | -84.255 | 277.27 |
| unpred*Treatment*Age*LHSS | Low_Tape | ASY | Nestling | 0 | . | . | . |
| unpred*Treatment*Age*LHSS | Low_Tape | SY | Mid Inc | 0 | . | . | . |
| unpred*Treatment*Age*LHSS | Low_Tape | SY | Nestling | 0 | . | . | . |
| unpred*Treatment*Age*LHSS | Predator | ASY | Mid Inc | 0 | . | . | . |
| unpred*Treatment*Age*LHSS | Predator | ASY | Nestling | 0 | . | . | . |
| unpred*Treatment*Age*LHSS | Predator | SY | Mid Inc | 0 | . | . | . |
| unpred*Treatment*Age*LHSS | Predator | SY | Nestling | 0 | . | . | . |

Table S22: Parameter estimates, standard error and confidence interval for the generalized linear mixed model for females negative feedback with total breeding season length as continuous. The model was fitted with a normal law and individual and experimental year specified as random factor. Breedinglength = Total breeding season length, Spl = Sample, Base = Baseline, Str = stress-induced, dex = post-dex, LHSS = Life history substage, Mid Inc = Mid Incubation, End incubation = End incubation, Nestling = Nestling provisioning, Rel_CI = relative clutch initiation date, Mass = Body mass

| Effect | Treatment | Age | LHSS | Estimate | SE | Lower | Upper |
| --- | --- | --- | --- | --- | --- | --- | --- |
|  |  |  |  |  |  |  |  |
| Intercept |  |  |  | -144.63 | 25.8879 | -195.55 | -93.7162 |
| breedinglength | |  |  | 0.557 | 0.2776 | 0.01078 | 1.1033 |
| Age |  | ASY |  | 21.8161 | 29.8816 | -36.9706 | 80.6028 |
| Age |  | SY |  | 0 | . | . | . |
| LHSS |  |  | Mid Inc | 31.3135 | 25.9363 | -19.773 | 82.4 |
| LHSS |  |  | Nestling | 0 | . | . | . |
| Treatment | Control |  |  | -17.2098 | 30.4735 | -77.1642 | 42.7446 |
| Treatment | Low_Tape |  |  | 22.302 | 39.5807 | -55.5793 | 100.18 |
| Treatment | Predator |  |  | 0 | . | . | . |
| Rel_CI |  |  |  | 0.4849 | 0.1845 | 0.122 | 0.8478 |
| Mass |  |  |  | 4.0273 | 0.7391 | 2.574 | 5.4806 |
| Str |  |  |  | -0.9097 | 0.0185 | -0.9461 | -0.8734 |
| breedinglenght*Age | | ASY |  | -0.2692 | 0.3711 | -0.9995 | 0.4611 |
| breedinglenght*Age | | SY |  | 0 | . | . | . |
| breedinglenght*LHSS | | | Mid Inc | -0.5529 | 0.3336 | -1.21 | 0.1042 |
| breedinglenght*LHSS | | | Nestling | 0 | . | . | . |
| breedinglenght*Treatment | Control |  |  | 0.1991 | 0.3727 | -0.5342 | 0.9324 |
| breedinglenght*Treatment | Low_Tape |  |  | -0.2713 | 0.4639 | -1.184 | 0.6415 |
| breedinglenght*Treatment | Predator |  |  | 0 | . | . | . |
| Age*LHSS | | ASY | Mid Inc | -41.6783 | 36.046 | -112.67 | 29.3181 |
| Age*LHSS | | ASY | Nestling | 0 | . | . | . |
| Age*LHSS | | SY | Mid Inc | 0 | . | . | . |
| Age*LHSS | | SY | Nestling | 0 | . | . | . |
| Treatment*Age | Control | ASY |  | 44.356 | 42.1016 | -38.4642 | 127.18 |
| Treatment*Age | Control | SY |  | 0 | . | . | . |
| Treatment*Age | Low_Tape | ASY |  | -8.3056 | 50.6538 | -107.97 | 91.3625 |
| Treatment*Age | Low_Tape | SY |  | 0 | . | . | . |
| Treatment*Age | Predator | ASY |  | 0 | . | . | . |
| Treatment*Age | Predator | SY |  | 0 | . | . | . |
| Treatment*LHSS | Control |  | Mid Inc | 6.297 | 36.7928 | -66.1657 | 78.7597 |
| Treatment*LHSS | Control |  | Nestling | 0 | . | . | . |
| Treatment*LHSS | Low_Tape |  | Mid Inc | -48.2163 | 44.8577 | -136.52 | 40.0904 |
| Treatment*LHSS | Low_Tape |  | Nestling | 0 | . | . | . |
| Treatment*LHSS | Predator |  | Mid Inc | 0 | . | . | . |
| Treatment*LHSS | Predator |  | Nestling | 0 | . | . | . |
| breedinglenght*Age*LHSS | | ASY | Mid Inc | 0.5309 | 0.4575 | -0.3702 | 1.432 |
| breedinglenght*Age*LHSS | | ASY | Nestling | 0 | . | . | . |
| breedinglenght*Age*LHSS | | SY | Mid Inc | 0 | . | . | . |
| breedinglenght*Age*LHSS | | SY | Nestling | 0 | . | . | . |
| breedinglenght*Treatment*Age | Control | ASY |  | -0.4654 | 0.5155 | -1.4797 | 0.5488 |
| breedinglenght*Treatment*Age | Control | SY |  | 0 | . | . | . |
| breedinglenght*Treatment*Age | Low_Tape | ASY |  | 0.186 | 0.601 | -0.9967 | 1.3687 |
| breedinglenght*Treatment*Age | Low_Tape | SY |  | 0 | . | . | . |
| breedinglenght*Treatment*Age | Predator | ASY |  | 0 | . | . | . |
| breedinglenght*Treatment*Age | Predator | SY |  | 0 | . | . | . |
| breedinglenght*Treatment*LHSS | Control |  | Mid Inc | -0.02561 | 0.4631 | -0.9377 | 0.8865 |
| breedinglenght*Treatment*LHSS | Control |  | Nestling | 0 | . | . | . |
| breedinglenght*Treatment*LHSS | Low_Tape |  | Mid Inc | 0.6382 | 0.5365 | -0.4182 | 1.6945 |
| breedinglenght*Treatment*LHSS | Low_Tape |  | Nestling | 0 | . | . | . |
| breedinglenght*Treatment*LHSS | Predator |  | Mid Inc | 0 | . | . | . |
| breedinglenght*Treatment*LHSS | Predator |  | Nestling | 0 | . | . | . |
| Treatment*Age*LHSS | Control | ASY | Mid Inc | -27.1105 | 51.0419 | -127.64 | 73.4181 |
| Treatment*Age*LHSS | Control | ASY | Nestling | 0 | . | . | . |
| Treatment*Age*LHSS | Control | SY | Mid Inc | 0 | . | . | . |
| Treatment*Age*LHSS | Control | SY | Nestling | 0 | . | . | . |
| Treatment*Age*LHSS | Low_Tape | ASY | Mid Inc | 59.3132 | 59.0089 | -56.8684 | 175.49 |
| Treatment*Age*LHSS | Low_Tape | ASY | Nestling | 0 | . | . | . |
| Treatment*Age*LHSS | Low_Tape | SY | Mid Inc | 0 | . | . | . |
| Treatment*Age*LHSS | Low_Tape | SY | Nestling | 0 | . | . | . |
| Treatment*Age*LHSS | Predator | ASY | Mid Inc | 0 | . | . | . |
| Treatment*Age*LHSS | Predator | ASY | Nestling | 0 | . | . | . |
| Treatment*Age*LHSS | Predator | SY | Mid Inc | 0 | . | . | . |
| Treatment*Age*LHSS | Predator | SY | Nestling | 0 | . | . | . |
| breedinglenght*Treatment*Age*LHSS | Control | ASY | Mid Inc | 0.1284 | 0.6448 | -1.1418 | 1.3986 |
| breedinglenght*Treatment*Age*LHSS | Control | ASY | Nestling | 0 | . | . | . |
| breedinglenght*Treatment*Age*LHSS | Control | SY | Mid Inc | 0 | . | . | . |
| breedinglenght*Treatment*Age*LHSS | Control | SY | Nestling | 0 | . | . | . |
| breedinglenght*Treatment*Age*LHSS | Low_Tape | ASY | Mid Inc | -0.8877 | 0.7167 | -2.2991 | 0.5238 |
| breedinglenght*Treatment*Age*LHSS | Low_Tape | ASY | Nestling | 0 | . | . | . |
| breedinglenght*Treatment*Age*LHSS | Low_Tape | SY | Mid Inc | 0 | . | . | . |
| breedinglenght*Treatment*Age*LHSS | Low_Tape | SY | Nestling | 0 | . | . | . |
| breedinglenght*Treatment*Age*LHSS | Predator | ASY | Mid Inc | 0 | . | . | . |
| breedinglenght*Treatment*Age*LHSS | Predator | ASY | Nestling | 0 | . | . | . |
| breedinglenght*Treatment*Age*LHSS | Predator | SY | Mid Inc | 0 | . | . | . |
| breedinglenght*Treatment*Age*LHSS | Predator | SY | Nestling | 0 | . | . | . |

**References**

Akçay, Ç., Lendvai, Á. Z., Stanback, M., Haussmann, M., Moore, I. T. & Bonier, F. 2016. Strategic adjustment of parental care in tree swallows: life-history trade-offs and the role of glucocorticoids. *Royal Society open science,* 3**,** 160740. 10.1098/rsos.160740.

Ardia, D. R. 2007. The ability to mount multiple immune responses simultaneously varies across the range of the tree swallow. *Ecography,* 30**,** 23-30. 10.1111/j.0906-7590.2007.04939.x.

Gow, E. A., Burke, L., Winkler, D. W., Knight, S. M., Bradley, D. W., Clark, R. G., Bélisle, M., Berzins, L. L., Blake, T., Bridge, E. S., Dawson, R. D., Dunn, P. O., Garant, D., Holroyd, G., Horn, A. G., Hussell, D. J. T., Lansdorp, O., Laughlin, A. J., Leonard, M. L., Pelletier, F., Shutler, D., Siefferman, L., Taylor, C. M., Trefry, H., Vleck, C. M., Vleck, D., Whittingham, L. A. & Norris, D. R. 2019. A range-wide domino effect and resetting of the annual cycle in a migratory songbird. *Proceedings of the Royal Society B: Biological Sciences,* 286**,** 20181916. doi:10.1098/rspb.2018.1916.

Peig, J. & Green, A. J. 2009. New perspectives for estimating body condition from mass/length data: the scaled mass index as an alternative method. *Oikos,* 118**,** 1883-1891. 10.1111/j.1600-0706.2009.17643.x.
